# Structural and functional consequences of aspartate/asparagine-β-hydroxylase variants causing Traboulsi syndrome

**DOI:** 10.1016/j.jbc.2025.111008

**Published:** 2025-12-05

**Authors:** Cynthia X. Hou, Amelia Brasnett, Patrick Rabe, Christopher J. Schofield, Lennart Brewitz

**Affiliations:** 1 Chemistry Research Laboratory and the Ineos Oxford Institute for Antimicrobial Research, University of Oxford, Oxford, United Kingdom; 2 Diamond Light Source, Diamond House, Harwell Science and Innovation Campus, Didcot, United Kingdom

**Keywords:** aspartate/asparagine-β-hydroxylase/AspH/BAH/HAAH, 2-oxoglutarate/α-ketoglutarate oxygenase, Traboulsi syndrome, epidermal growth factor–like domain, rare disease, Marfan syndrome, hypoxia, dioxygenase, post-translational modification, enzyme mutation

## Abstract

Traboulsi syndrome is an autosomal recessive hereditary disease associated with developmental defects, in particular of the ocular system. SNPs affecting the *ASPH* gene, which encodes for the 2-oxoglutarate (2OG)-dependent oxygenase aspartate/asparagine-β-hydroxylase (AspH), are associated with Traboulsi syndrome. AspH catalyzes hydroxylations of conserved aspartate/asparagine residues in epidermal growth factor–like domain (EGFD) proteins. We report studies on the clinically observed Traboulsi syndrome–associated R688Q, R735Q, and R735W AspH variants. The results reveal that pathogenic active site substitutions substantially reduce, though do not ablate, EGFD hydroxylase activity compared with wt AspH. They imply that efficient AspH-catalyzed EGFD hydroxylation is important during human development. Crystallographic studies reveal conservation of the overall AspH fold, but that the preferred conformations of 2OG in complex with the R735Q and R735W AspH variants differ from those with wt AspH. Screening of potential 2OG cosubstrate substitutes reveals that certain 2-oxoacids, including naturally present metabolites, manifest enhanced catalytic efficiency of Traboulsi syndrome–associated AspH variants compared with 2OG. The results thus provide proof of principle for a therapeutic strategy involving rescue of impaired activities of pathogenic active site AspH variants by use of 2-oxoacids, or 2-oxoacid precursors, other than 2OG.

Traboulsi syndrome (Online Mendelian Inheritance in Man (OMIM): 601552) is an autosomal recessive hereditary disease, which has been definitely diagnosed in ∼40 to 60 individuals worldwide (1, 2, 3), though literature analyses suggest that it is more widespread than currently perceived because of a lack of genetic profiling of potentially affected individuals (4, 5, 6, 7, 8). Traboulsi syndrome is associated with developmental defects of the ocular system (*e*.*g*., ectopia lentis) (1, 2, 9, 10, 11, 12, 13, 14, 15, 16, 17, 18, 19, 20, 21), craniofacial dysmorphism (1, 2, 9, 10, 12, 13, 14, 15, 16, 17, 19, 21, 22, 23, 24, 25), aortic dilatation (9, 21), and aortic regurgitation (2, 9, 12, 15, 21). The exact phenotype and severity of Traboulsi syndrome vary on a case-to-case basis. There is no effective treatment for Traboulsi syndrome, with current therapeutic interventions to ameliorate Traboulsi syndrome–associated ectopia lentis relying on surgery (26), the success of which is limited (11, 12, 15, 16, 17, 18).

The genetic causes for Traboulsi syndrome are pleiotropic, but all affect the *ASPH* gene that encodes for the multidomain 2-oxoglutarate (2OG)- and Fe(II)-dependent oxygenase aspartate/asparagine-β-hydroxylase (AspH), as well as for C-terminally truncated AspH splice variants lacking the C-terminal 2OG oxygenase domain, *e.g.*, junctate, junctin, and humbug (27, 28, 29, 30). Genetic analyses imply that developmental defects associated with Traboulsi syndrome are a result of impaired AspH oxygenase catalysis (1, 23). This proposal is, at least in part, supported by animal model studies: mice in which *ASPH* is deleted develop defects, which in part reflect symptoms of Traboulsi syndrome patients, although ocular phenotypes were not observed (31). The proposal that the developmental defects associated with Traboulsi syndrome are a result of impaired AspH catalysis has, however, not yet been validated at a biochemical level using purified AspH variants.

Pioneering studies on oxidative post-translational modifications to proteins revealed that AspH catalyzes stereospecific post-translational C-3 hydroxylation of aspartate/asparagine residues in epidermal growth factor–like domains (EGFDs) (Fig. 1*A*) (32, 33, 34), including human coagulation factor X (hFX) (35, 36), notch (37), and the extracellular matrix proteins, fibrillin-1, -2, and -3 (FBN1–3) (38, 39, 40). EGFDs are small protein domains (∼30–50 residues) present in secreted and cell surface–bound proteins. The EGFD fold is stabilized by three intradomain disulfide linkages (41, 42, 43), which typically manifest a C1–C3, C2–C4, and C5–C6 disulfide connectivity (Fig. S1*A*) (44, 45, 46). Biochemical and proteomic mass spectrometric (MS) studies indicate that the extent of EGFD hydroxylation varies in assigned AspH substrates, ranging from apparently none to complete hydroxylation (40, 47, 48). Unlike the function of post-translational EGFD glycosylation, which affects, *e.g.*, protein secretion (49, 50, 51), the function(s) of AspH-catalyzed EGFD hydroxylation are unknown.

**Figure 1:**
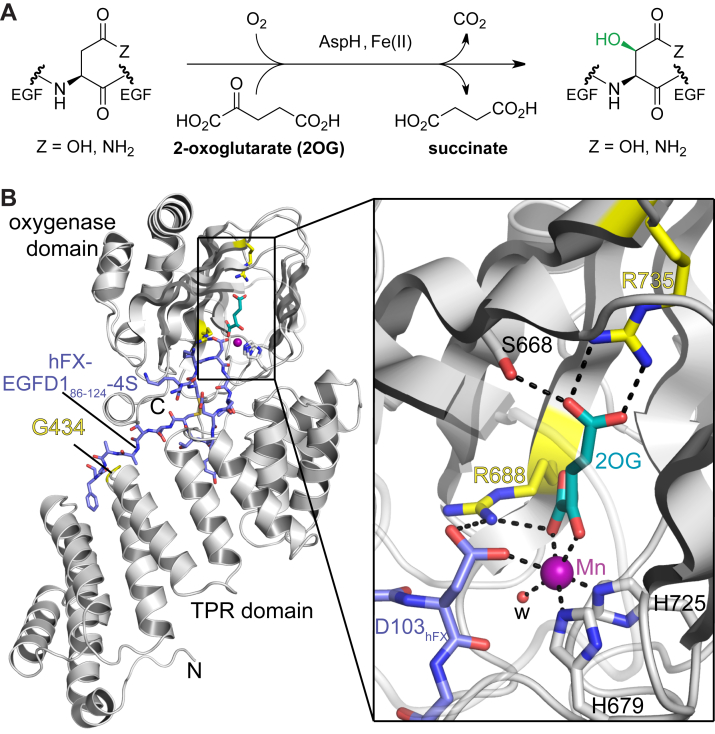
**Locations of Traboulsi syndrome–associated AspH variants**. *A*, the AspH catalyzed reaction; (*B*) view from a reported wt AspH:2OG:Mn:hFX-EGFD1_86–124_-4S structure (PDB ID: 6YYW) (65) showing positions of Traboulsi syndrome–associated active site variations (R735W/R735Q and R688Q) and tetratricopeptide repeat (TPR) domain variations (G434V) in *yellow*. Colors: *gray*: AspH_315–758_; *teal*: carbon backbone of 2OG; *violet*: Mn, substitutes for the active cofactor Fe(II); *slate blue*: carbon backbone of the hFX-derived hFX-EGFD1_86–124_-4S peptide (Fig. S1*C*). AspH, aspartate/asparagine-β-hydroxylase; EGFD, epidermal growth factor–like domain; hFX, human coagulation factor X; 2OG, 2-oxoglutarate; PDB, Protein Data Bank.

Crystallographic and kinetic studies with an N-terminally truncated form of AspH (*i*.*e*., AspH_315–758_) have revealed that recombinant AspH only accepts substrates with a noncanonical C1–C2, C3–C4, and C5–C6 EGFD disulfide connectivity (Fig. S1*B*) (40, 52). AspH may thus enable EGFD disulfide isomerization in the endoplasmic reticulum (40, 52). Biochemical and crystallographic studies have revealed an important role in substrate binding for the noncatalytic tetratricopeptide repeat (TPR) AspH domain, which is located N terminal to its 2OG oxygenase domain (Fig. 1*B*) (52); N-terminally truncated AspH constructs comprising only the 2OG oxygenase domain do not catalyze EGFD substrate oxidation *in vitro* (52).

Kinetic studies indicate that AspH has potential capacity for oxygen/hypoxia sensing (47). Two types of human 2OG oxygenases play key roles within the hypoxia-inducible factor (HIF) hypoxia sensing and response system (53, 54). Catalysis by HIF-α prolyl hydroxylase domain enzymes signals for HIF-α degradation, so limiting transcription by α,β-HIF (53). Factor inhibiting hypoxia-inducible factor-α (FIH) is a 2OG-dependent protein hydroxylase, which, like AspH, catalyzes C-3 hydroxylation of aspartate/asparagine residues, including an asparagine residue in HIF-1α (N803 in human HIF-1α) and HIF-2α isoforms (55), a modification apparently suppressing HIF-mediated transcription (53, 56, 57). FIH catalyzes aspartate/asparagine residue hydroxylation, though with a different stereospecificity to AspH (58). In addition to catalyzing hydroxylation in unstructured regions of HIF-1α and HIF-2α (59, 60), FIH modifies ankyrin repeat domains present in notch and many other proteins (61, 62).

Three Traboulsi syndrome–associated SNPs affect *ASPH* codons encoding for AspH active site residues directly involved in 2OG binding, *i.e.*, R688Q (11), R735Q (12, 23), and R735W (1, 24) (Fig. 1*B*). The Traboulsi syndrome–associated G692D AspH variant was recently reported, though analysis of its effect using the wt AspH structure suggests that it likely will not directly interfere with 2OG or Fe(II) binding (25, 63). Another SNP in the *ASPH* gene is located in the TPR domain of AspH, *i.e.*, G434V (64). The G434V AspH variant was detected in an individual who, in addition to lens subluxation, also manifested symptoms not normally associated with Traboulsi syndrome, *e.g.*, chronic kidney disease and vesicoureteral reflux (64).

Crystal structures of wt AspH reveal that the R735 and R688 side chains are directly involved in the binding of 2OG; the R688 side chain is also positioned to bind to the EGFD substrate (Fig. 1*B*) (52, 65). These observations indicate that the Traboulsi syndrome–associated AspH active site variations could, at least in principle, impair catalysis by altering 2OG binding to AspH (1, 23). It was recently reported that AspH variants with only a single protein-bound Fe(II) binding histidine residue, *i.e.*, H679A and H725A AspH, unexpectedly retain catalytic activity (66, 67). Although the H679A and H725A AspH variants have not been detected in humans, the H679A AspH variant has been frequently used in cellular studies to reduce AspH activity; consistent with our *in vitro* results (66), H679A AspH is reported to retain some catalytic activity in cells (68). This observation raises the question as to what extent Traboulsi syndrome–associated AspH active site variants are catalytically competent and to whether Traboulsi syndrome–associated developmental defects are exclusively a result of impaired AspH catalysis or of more complex interactions with (substrate) proteins.

Here, we report biochemical and crystallographic studies on clinically observed Traboulsi syndrome–associated variants, including R688Q, R735Q, and R735W AspH. These variants are substantially less active than wt AspH, despite having the same 2OG oxygenase domain fold as observed for wt AspH. Following crystallographic studies revealing that the Traboulsi syndrome–associated variations in the AspH active site can alter the nature of 2OG binding, we demonstrate proof-of-principle evidence showing the potential of synthetic and naturally occurring 2-oxoacids, including amino acid transamination products abundant in cells, to compensate for the reduced activity of Traboulsi syndrome–associated variants, highlighting the therapeutic possibility of rescuing the activity of pathogenic AspH variants.

## Results

### Traboulsi syndrome–associated active site AspH variants are less active than wt AspH

The three reported pathogenic Traboulsi syndrome–associated SNPs, corresponding to the R735W (1, 24), R735Q (12, 23), and R688Q AspH (11), were introduced by standard methods using a construct of human wt AspH encoding for His_6_-AspH_315–758_ (52). The corresponding AspH variants were recombinantly produced in *Escherichia coli* and purified to near homogeneity by Ni(II)-affinity and subsequent size-exclusion chromatography (>95% purity by SDS-PAGE and MS analysis; Figs. S2-3). We also prepared the G434V AspH variant that is located in the TPR domain (64), which is involved in substrate binding (Fig. 1*B*) (52).

**Figure 2:**
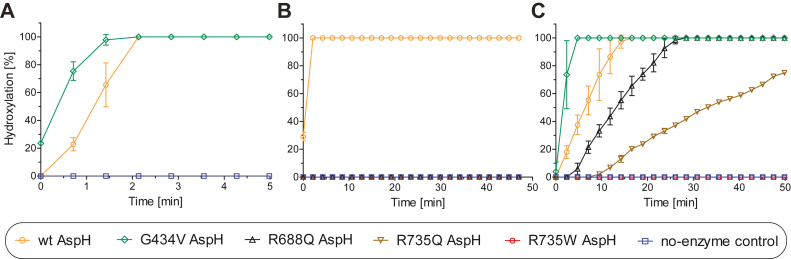
**Activities of Traboulsi syndrome–associated active site AspH variants are substantially reduced compared with wt AspH**. *A*, using wt AspH (*orange circles*; 0.1 μM) or G434V AspH (0.1 μM), hFX-EGFD1_86–124_-4S (4.0 μM), l-ascorbic acid (LAA; 100 μM), 2OG (20 μM), Fe(II) (20 μM) in buffer (25 mM Hepes, 50 mM NaCl, pH 7.5); (*B*) using wt AspH (*orange circles*; 0.1 μM) or an AspH variant (R688Q, *black triangles*; R735Q, *brown inverse triangles*; and R735W, *red hexagons*; 0.1 μM), hFX-EGFD1_86–124_-4S (4.0 μM), LAA (100 μM), 2OG (20 μM), Fe(II) (20 μM) in buffer (25 mM Hepes, 50 mM NaCl, pH 7.5); (*C*) using wt AspH (*orange circles*; 0.2 μM) or an AspH variant (G434V, *green diamonds*; R688Q, *black triangles*; R735Q, *brown inverse triangles*; and R735W, *red hexagons*; 0.2 μM), hFX-EGFD1_86–124_-4S (4.0 μM), LAA (400 μM), 2OG (30 μM or 2000 μM for R735Q and R735W AspH), Fe(II) (100 μM) in buffer (25 mM Mes, pH 6.0); and *blue boxes*: no-enzyme control. Measurement times were normalized to the first sample injection analyzed after the addition of AspH to the substrate mixture (*t* = 0 min), by which time low levels of hydroxylation were manifest; results are means of three independent runs (n = 3; mean ± SD). AspH, aspartate/asparagine-β-hydroxylase; EGFD, epidermal growth factor–like domain; hFX, human coagulation factor X; 2OG, 2-oxoglutarate.

The ability of the R735Q/W, R688Q, and G434V AspH variants to catalyze 2OG oxidation uncoupled from that of substrate oxidation was investigated by ^1^H NMR. The results for the four tested AspH variants reveal low levels of “EGFD substrate uncoupled” oxidation of 2OG to give succinate, at a similar level to that observed for wt AspH (Fig. S4) (52). The hydroxylase activities of the four AspH variants were then monitored using solid phase extraction coupled to mass spectrometry (SPE–MS)–based assays, which directly monitor the +16 Da mass shift associated with hydroxylated product formation from an EGFD-derived peptide (Fig. 2*A*) (47). The synthetic disulfide–bridged oligopeptide hFX-EGFD1_86–124_-4S (Fig. S1*C*) (52), the sequence of which is based on the EGFD1 of the reported AspH substrate hFX (35, 36), was used as a substrate. The results reveal that the activities of the G434V AspH variant and wt AspH were similar using reported assay conditions (*i*.*e*., AspH variant (0.1 μM), hFX-EGFD1_86–124_-4S (4.0 μM), l-ascorbic acid (LAA, 100 μM), 2OG (20 μM), and Fe(II) (20 μM) in buffer (25 mM Hepes, 50 mM NaCl, pH 7.5) at 20 °C) (66). By contrast, the R735Q, R735W, and R688Q AspH variants were inactive under the same conditions (Fig. 2, *A* and *B*).

Assays following individual optimization of cosubstrate/cofactor concentrations and buffer composition reveal that catalysis by R735Q and R688Q AspH is more efficient under slightly acidic conditions (25 mM Mes, pH 6.0, compared with 25 mM Hepes, 50 mM NaCl, pH 7.5 for wt AspH) in the absence of NaCl, using higher cosubstrate/cofactor concentrations than used for wt AspH (Fig. 2*C*). Even under the partially optimized conditions, catalysis by R735Q and R688Q AspH was less efficient than that of wt AspH and G434V AspH (Fig. 2*C*). Removal of the N-terminal His_6_-tag using thrombin (69) did not substantially alter the activity of R735Q AspH, and, by implication, likely of the other tested AspH variants (Fig. S3). Interestingly, R735W AspH was inactive under all the tested conditions, supporting the proposal that developmental defects associated with Traboulsi syndrome are a direct consequence of impaired AspH catalysis (Fig. 2, *B* and *C*). Levels of G434V, R688Q, and R735Q AspH catalysis under the optimized conditions were sufficient to enable kinetic studies.

### Kinetic studies on AspH variants

**Figure 3:**
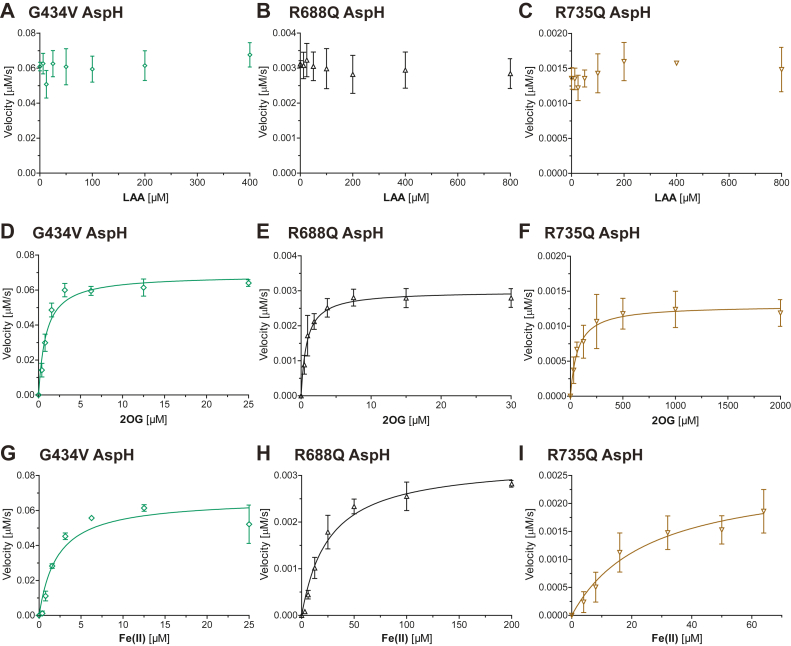
**Kinetic parameters for the AspH variant–catalyzed hydroxylation of hFX-EGFD1**_**86–124**_**-4S using SPE–MS**. *A*–*I*, determination of the G434V (*green diamonds*), R688Q (*black triangles*), and R735Q (*brown inverse triangles*) AspH variant $v_{\max}^{\text{app}}$ and $K_{m}^{\text{app}}$ values for (*A*–*C*) LAA, (*D*–*F*) 2OG, and (*G*–*I*) Fe(II). Assays employed 0.1 μM (G434V) or 0.2 μM (R688Q, R735Q) AspH variant; details are described in the Experimental procedures section. Catalysis by the tested AspH variants did not depend on LAA. The initial hydroxylation rates used to determine kinetic parameters are shown in Figures S5–S7. The results are means of three independent runs (n = 3; mean ± SD). AspH, aspartate/asparagine-β-hydroxylase; EGFD, epidermal growth factor–like domain; hFX, human coagulation factor X; LAA, l-ascorbic acid; 2OG, 2-oxoglutarate; SPE–MS, solid phase extraction coupled to mass spectrometry.

Kinetic studies employing SPE–MS assays were used to rank wt AspH and the G434V, R688Q, and R735Q AspH variants according to catalytic efficiency. Apparent turnover numbers ($k_{\text{cat}}^{\text{app}}$), Michaelis constants ($K_{m}^{\text{app}}$), and specificity constants ($k_{\text{cat}}^{\text{app}}$/$K_{m}^{\text{app}}$) of G434V, R688Q, and R735Q AspH for both 2OG and Fe(II) were determined under ambient O_2_ conditions. Although catalysis by the tested AspH variants did not depend on LAA under the tested conditions (Fig. 3, *A*–*C*), in accord with results for wt AspH (47), assays were performed with LAA because its presence increases assay robustness for wt AspH (47). Note that care should be taken in the mechanistic interpretation of kinetic parameters in the context of the complex AspH reaction, including because of the different assay conditions (see above) and because our previous results have shown that the ambient O_2_ concentration is not saturating for wt AspH (47), implying similar behavior with the AspH variants tested here. We have not investigated the O_2_ dependence of the AspH variants, including because of their relatively low reactivity (Fig. 2). Nonetheless, the results of the kinetic studies revealed clear differences, at least in some cases, for the clinically observed variants, as described below.

The results reveal that the G434V AspH $k_{\text{cat}}^{\text{app}}$ values are consistently ∼2-fold greater than for wt AspH determined under identical conditions (Table 1). By contrast, the R688Q and R735Q AspH $k_{\text{cat}}^{\text{app}}$ values, which were obtained using different conditions, are at least ∼20-fold lower than for wt AspH (Table 1). Note that $k_{\text{cat}}^{\text{app}}$ values were determined assuming that all the used AspH was similarly active, as supported by wt AspH active site titration using a tight-binding small-molecule inhibitor (47). Notably, the G434V, R688Q, R735Q, and wt AspH $k_{\text{cat}}^{\text{app}}$ values were consistent throughout all experiments (Table 1), reflecting the robustness of the SPE–MS assays.

**Table 1:** Kinetic parameters of the G434V, R688Q, and R735Q AspH variants for 2OG and Fe(II)

Entry	Cosubstrate/cofactor	AspH variant	$k_{\text{cat}}^{\text{app}}$ (s^-1^)	$K_{m}^{\text{app}}$ (μM)	$k_{\text{cat}}^{\text{app}}$/$K_{m}^{\text{app}}$ (mM^-1^ s^-1)^
i	2OG	wt (66)	0.31 ± 0.03	1.1 ± 0.4	280 ± 110
		G434V^a^	0.69 ± 0.03	0.89 ± 0.13	770 ± 110
		R688Q^b^	0.015 ± 0.001	0.81 ± 0.15	18 ± 4
		R735Q^c^	0.006 ± 0.001	70 ± 20	0.09 ± 0.03
ii	Fe(II)	wt (66)	0.27 ± 0.04	4.3 ± 1.6	63 ± 25
		G434V^a^	0.67 ± 0.05	2.2 ± 0.5	310 ± 71
		R688Q^b^	0.016 ± 0.001	26 ± 5	0.63 ± 0.11
		R735Q^c^	0.013 ± 0.003	26 ± 10	0.50 ± 0.21

SPE–MS analyses are shown in Figures S5–S7, Michaelis–Menten curves used to determine kinetic parameters are shown in Figure 3. Results are means of independent triplicates (n = 3; mean ± SD).

a G434V His_6_-AspH_315–758_ (0.1 μM), hFX-EGFD1_86–124_-4S (4.0 μM), LAA (100 μM), and varying Fe(II) or 2OG concentrations in buffer (25 mM Hepes, pH 7.5, 50 mM NaCl; 20 °C).

b R688Q His_6_-AspH_315–758_ (0.2 μM), hFX-EGFD1_86–124_-4S (4.0 μM), LAA (400 μM), and varying Fe(II) or 2OG concentrations in buffer (25 mM Mes, pH 6.0; 20 °C).

c R735Q His_6_-AspH_315–758_ (0.2 μM), hFX-EGFD1_86–124_-4S (4.0 μM), LAA (400 μM), and varying Fe(II) or 2OG concentrations in buffer (25 mM Mes, pH 6.0; 20 °C).

The G434V, R688Q, and wt AspH $K_{m}^{\text{app}}$ values for 2OG are similar despite the variations in reaction conditions (Table 1, entry i), indicating these variants may have similar 2OG affinities. By contrast, the R735Q AspH $K_{m}^{\text{app}}$ value for 2OG is >60-fold higher than that reported for wt AspH (66) (Table 1, entry i), an observation that may reflect the reduced ability of the 2OG C-5 carboxylate to interact with the side chain of Q735, likely resulting in reduced 2OG affinity for R735Q AspH. In principle, the different conditions used when monitoring catalysis of R735Q and wt AspH (0.2 μM R735Q AspH in 50 mM Mes buffer, pH 6.0 *versus* 0.1 μM wt AspH in 25 mM Hepes buffer, 50 mM NaCl, pH 7.5) may complicate comparison of the kinetic parameters; however, the kinetic parameters of the R688Q and R735Q AspH variants were determined under identical conditions, suggesting that the tested assay conditions do not substantially affect the determination of the 2OG $K_{m}^{\text{app}}$ values.

Although the R735Q AspH $K_{m}^{\text{app}}$ value for 2OG is higher than those of wt AspH and many other human 2OG oxygenases (47, 66, 70), it is lower than that for the dimeric γ-butyrobetaine hydroxylase (∼150–470 μM), for which consistently high $K_{m}^{\text{app}}$ values for 2OG have been reported (71, 72, 73). Notably, the R735Q AspH $K_{m}^{\text{app}}$ value for 2OG is lower than the approximate 2OG concentration in cells (up to >1 mM (74, 75)), indicating that 2OG may bind to R735Q AspH in cells; conversely, the R735Q AspH variant may manifest activity in cells.

The G434V $k_{\text{cat}}^{\text{app}}$/$K_{m}^{\text{app}}$ value for 2OG is >2-fold greater than that for wt AspH, indicating that catalysis of G434V AspH is more efficient than that of wt AspH, at least with hFX-EGFD1_86–124_-4S (52) (Table 1, entry i). The biological consequences of this observation are unknown, but it further highlights the importance of the TPR domain in AspH catalysis. By contrast, our results reveal that the wt AspH $k_{\text{cat}}^{\text{app}}$/$K_{m}^{\text{app}}$ value for 2OG is ∼15- and >3000-fold greater than those of R688Q and R735Q AspH, respectively. Thus, the substitution of the R735 guanidinium group has apparently a more pronounced effect on AspH catalysis than the R688Q substitution, potentially indicating why Traboulsi syndrome–associated SNPs in the *ASPH* codon for R735 are apparently more frequently detected than SNPs in other *ASPH* codons (25). The results thus raise the question as to whether the severity of Traboulsi syndrome–associated phenotypes varies among individuals bearing the R688Q or R735Q/W AspH variants.

The results reveal that the G434V AspH $K_{m}^{\text{app}}$ value for Fe(II) was ∼2-fold lower than that of wt AspH, indicating that sequence variations in the TPR domain distal to the AspH active site have the potential to alter the stability of the AspH:2OG:Fe(II):substrate quaternary complex, potentially *via* affecting substrate binding (Fig. 1; Table 1, entry ii) (52). It may be that the effect of the G434V variation on AspH catalysis varies with the substrate employed, considering that EGFDs in ∼100 human proteins fulfill the proposed requirements for productive AspH catalysis (40) and that EGFDs typically share a low overall sequence similarity (41). By contrast to G434V AspH, the R688Q and R735Q AspH $K_{m}^{\text{app}}$ values for Fe(II) were ∼6-fold higher than those of wt AspH (Table 1, entry ii), suggesting that alterations in 2OG binding may indirectly affect Fe(II) binding, potentially *via* reducing the stability of the ternary AspH:Fe(II):2OG complex.

The G434V, R688Q, R735Q, and wt AspH $k_{\text{cat}}^{\text{app}}$/$K_{m}^{\text{app}}$ values for Fe(II) vary substantially (Table 1, entry ii). The G434V AspH $k_{\text{cat}}^{\text{app}}$/$K_{m}^{\text{app}}$ values for Fe(II) are ∼5-fold higher than that of wt AspH, which itself is >100-fold higher than for R688Q and R735Q AspH (Table 1, entry ii). The observation that the R688Q and R735Q AspH $k_{\text{cat}}^{\text{app}}$/$K_{m}^{\text{app}}$ values for Fe(II) are similar, whereas those for 2OG differ by ∼200-fold, indicates that the R735 side chain is important for efficient binding of both 2OG and Fe(II). The side chain of R688 is apparently more important for Fe(II) binding than 2OG binding, although its guanidinium group is positioned to directly interact with the 2OG C-1 carboxylate, as indicated by analysis of the wt AspH:2OG complex crystal structure (52, 65). Note that, unlike R735, R688 is directly involved in substrate binding (52, 65); variations in the R688 side chain may thus affect the stability of the quaternary AspH:2OG:Fe(II):substrate complex.

### Traboulsi syndrome–associated variations of R735 affect the nature of 2OG binding with AspH

Having shown that both the R688Q and R735Q AspH variants manifest reduced activity (Table 1, Fig. 3), we investigated the consequences of the Traboulsi syndrome–associated active site variations on the AspH fold and 2OG binding using crystallography.

The R688Q, R735Q, and R735W AspH variants were crystallized in the presence of Mn(II) (substituting for the catalytically active Fe(II)), 2OG, and the synthetic disulfide–bridged hFX-EGFD1_86–124_-4S substrate (Fig. S1*C*) (52). R735Q and R735W AspH crystallized in the *P*2_1_2_1_2_1_ space group (1.95 and 1.92 Å resolution, respectively; Figs. S8-S9, Table S1), in accord with reported wt AspH structures (52, 65). The R735Q and R735W AspH:2OG:Mn:hFX-EGFD1_86–124_-4S structures were solved by molecular replacement using a reported wt AspH structure as a search model (Protein Data Bank (PDB) ID: 6YYW (65)). Notably, the R688Q AspH variant did not crystallize despite extensive investigations. To enable more precise structural comparisons, we obtained a wt AspH:2OG:Mn:hFX-EGFD1_86–124_-4S structure with an improved resolution compared with that of our reported structure (52) (1.84 *versus* 2.27 Å resolution; Fig. S10, Table S1).

**Figure 4:**
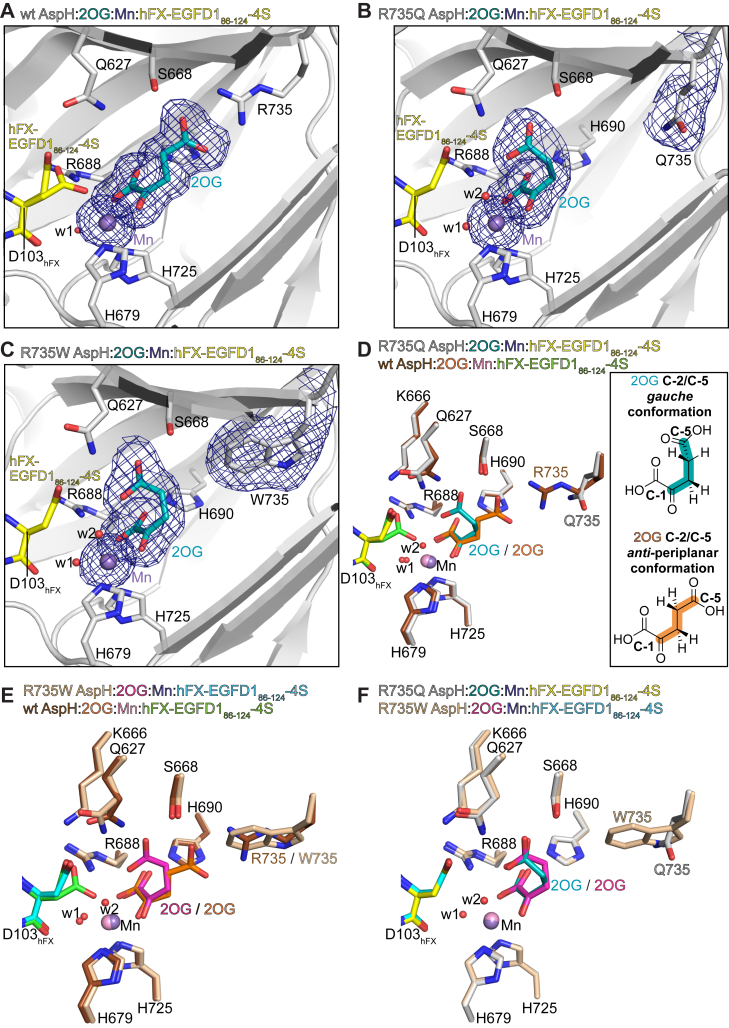
**The conformation of 2OG in complex with the Traboulsi syndrome–associated R735Q and R735W AspH variants differs from that observed in complex with wt AspH**. Colors: *gray*: wt and variant AspH_315–758_; *teal*: carbon backbone of 2OG; *lavender*: Mn; *yellow*: carbon backbone of the hFX-EGFD1_86–124_-4S peptide (Fig. S1*C*); *red*: oxygen; *blue*: nitrogen; and w: water. *A*–*C*, representative Polder omit density maps contoured to 3.0σ around (*A*) 2OG and Mn in the wt AspH:2OG:Mn:hFX-EGFD1_86–124_-4S structure, (*B*) 2OG, Mn, and Q735 in the R735Q AspH:2OG:Mn:hFX-EGFD1_86–124_-4S structure, and (*C*) 2OG, Mn, and W735 in the R735W AspH:2OG:Mn:hFX-EGFD1_86–124_-4S structure; (*D*–*F*) superimposition of active site views of (*D*) the R735Q AspH:2OG:Mn:hFX-EGFD1_86–124_-4S (colors: *gray*: R735Q AspH; *teal*: 2OG; *lavender*: Mn; *yellow*: carbon backbone of hFX-EGFD1_86–124_-4S) and wt AspH:2OG:Mn:hFX-EGFD1_86–124_-4S structures (colors: *brown*: R735Q AspH; *orange*: 2OG; *pink*: Mn; *light green*: carbon backbone of hFX-EGFD1_86–124_-4S); (*E*) the R735W AspH:2OG:Mn:hFX-EGFD1_86–124_-4S (colors: *ochre*: R735Q AspH; *magenta*: 2OG; *lavender*: Mn; *light blue*: carbon backbone of hFX-EGFD1_86–124_-4S) and wt AspH:2OG:Mn:hFX-EGFD1_86–124_-4S structures, and (*F*) the R735Q AspH:2OG:Mn:hFX-EGFD1_86–124_-4S and R735W AspH:2OG:Mn:hFX-EGFD1_86–124_-4S structures. AspH, aspartate/asparagine-β-hydroxylase; EGFD, epidermal growth factor–like domain; hFX, human coagulation factor X; 2OG, 2-oxoglutarate.

As anticipated, in the 1.84 Å resolution wt AspH:2OG:Mn:hFX-EGFD1_86–124_-4S crystal structure, the imidazole rings of both H679 (2.3 Å) and H725 (2.2 Å) coordinate to Mn, which also binds a water molecule (w1; 2.1 Å) (Fig. 4*A*). Consistent with some reported AspH:Mn:hFX-EGFD1_86–124_-4S structures (52, 65, 76, 77), the side chain of the hFX-EGFD1_86–124_-4S substrate D103_hFX_ residue, *i.e.*, the hydroxylation site, is observed in two conformations (Fig. 4*A*). In one conformation, the D103_hFX_ side chain carboxylate is positioned to interact with the side-chain carboxamide of AspH Q627 (3.1 Å), leaving a vacant Mn coordination site, possibly reflecting that to which O_2_ binds during catalysis. In the other conformation, the D103_hFX_ side-chain carboxylate coordinates the Mn *trans* to H725 (2.6 Å). In both conformations, the 2OG C-1 carboxylate coordinates the Mn *trans* to H679 (2.1 Å) and is positioned to interact with H690 (2.7 Å) and R688 (2.7 Å); the 2OG C-1 carboxylate is also positioned to interact with the side-chain carboxylate of D103_hFX_ (3.1 Å) in one of the two hFX-EGFD1_86–124_-4S conformations. The 2OG C-2 ketone oxygen atom coordinates to Mn (2.2 Å) *trans* to the complexed water molecule w1 (1.9 Å) (Fig. 4*A*). The 2OG C-5 carboxylate is positioned to form a salt bridge with the guanidinium group of R735 (2.7 Å) and to interact with the side chain of S668 (2.5 Å). Similar 2OG C-5 carboxylate binding arrangements have been observed in crystal structures of some other human 2OG oxygenases (78). Note that the 2OG conformation in the 1.84 Å resolution wt AspH:2OG:Mn:hFX-EGFD1_86–124_-4S structure differs from that in the reported wt AspH structure (52) (Fig. S11).

Analysis of the electron density maps of the R735Q and R735W AspH:2OG:Mn:hFX-EGFD1_86–124_-4S structures reveals the anticipated R735 substitutions (Fig. 4), in accord with MS and sequencing analyses (Fig. S2). Comparison with the wt AspH:2OG:Mn:hFX-EGFD1_86–124_-4S structure indicates that the R735Q and R735W substitutions do not affect the overall fold (RMSD ∼0.30 Å for both R735Q and R735W AspH; Figs. S12-S14). Likewise, the AspH R735Q and R735W substitutions did not substantially alter the conformation of hFX-EGFD1_86–124_-4S (RMSD: ∼0.24 Å and ∼0.20 Å for R735Q and R735W AspH, respectively; Figs. S12-S14).

Analysis of the electron density maps for 2OG in the R735Q and R735W AspH:2OG:Mn:hFX-EGFD1_86–124_-4S structures reveals that the conformation of the 2OG C-1 and C-2 carbon atoms and the distances between Mn and the 2OG C-1 carboxylate and C-2 ketone groups in the R735Q (2.1 and 2.2 Å) and R735W (2.1 and 2.3 Å) AspH structures are similar to those in the wt AspH (2.1 and 2.2 Å) structure (Fig. 4). The distances between Mn and H679/H725 are identical in the R735Q and R735W AspH structures (H679: 2.3 Å; H725: 2.1 Å) and similar to those of wt AspH (H679: 2.3 Å; H725: 2.2 Å). By contrast, the conformations of the 2OG C-5 carboxylate and the C-4/C-3 methylene groups in both the R735Q and the R735W AspH:2OG:Mn:hFX-EGFD1_86–124_-4S structures are similar, but not identical to each other, and differ substantially from those for 2OG in complex with wt AspH. The dihedral angles formed by the 2OG C-2/C-3/C-4/C-5 atoms are ∼61° and ∼57° in the R735Q and R735W AspH structures, respectively, indicative of a *gauche* arrangement of C-2 and C-5 around the 2OG C-3/C-4 bond (Fig. 4*D*). By contrast, the dihedral angle formed by the 2OG C-2/C-3/C-4/C-5 atoms is ∼174° in the wt AspH structure, indicative of an antiperiplanar conformation around the 2OG C-3/C-4 bond (Fig. 4*D*). Note that the 2OG conformation in complex with the R735Q and R735W AspH variants may differ when performing crystallizations in the presence of Fe(II) instead of Mn(II).

In the R735Q and R735W AspH structures, the 2OG C-5 carboxylate oxygens are positioned to interact with the side chains of S668 (2.6 Å in both structures), Q627 (3.1 and 2.8 Å, respectively), and W625 (3.1 and 2.9 Å, respectively), resulting in 2OG conformations in which the C-5 carboxylate carbon is oriented away from the Q735 carboxamide carbon (9.0 Å) or the W735 indole nitrogen (9.0 Å) (Fig. 4). Superimposition of the R735Q, R735W, and wt AspH AspH:2OG:Mn:hFX-EGFD1_86–124_-4S structures reveals that the Q627 side chain adopts a conformation that apparently enables more efficient interaction with the 2OG C-5 carboxylate in the R735Q/W AspH variants (Fig. 4). The position of the Q627 carboxamide carbon in the R735Q and R735W AspH structures differs by 0.9 Å from that observed with wt AspH. The 2OG C-5 carboxylate is also positioned to interact with water w2 in both the R735Q and R735W AspH:2OG:Mn:hFX-EGFD1_86–124_-4S structures (2.7 and 2.8 Å, respectively; Fig. 4). Water w2 binds to Mn (2.1 Å in both structures) at the position where O_2_ is predicted to bind during catalysis (52); the interaction of w2 with the 2OG C-5 carboxylate may thus stabilize its binding to the active site metal ion, potentially reducing the catalytic efficiency of the R735Q and R735W AspH variants. Notably, comparison with the wt AspH structure indicates that w2 competes with the D103_hFX_ side-chain carboxylate for binding to Mn (Fig. 4). The potentially enhanced stability of the Mn–w2 interaction in the R735Q and R735W AspH variant structures may help rationalize why the D103_hFX_ side chain only occupies one conformation in the R735Q and R735W AspH structures but is observed in two conformations in several reported wt AspH structures (52, 65, 76).

The *gauche* conformation adopted by 2OG when complexed with the R735Q and R735W AspH variants likely reflects the absence of stabilizing salt bridge interactions between the 2OG C-5 carboxylate and the R735Q/W side chains. The observation that the R735W AspH variant is inactive under the tested conditions suggests that the 2OG *gauche* conformation does not sustain productive AspH catalysis (Fig. 2). With R735Q AspH, it is possible that 2OG occupies a less stable, but catalytically productive, conformation with its C-5 carboxylate oriented toward the Q735 carboxamide group, consistent with the observed, but reduced, activity for R735Q AspH (Table 1). By contrast to R735Q AspH, the population of such an alternative, catalytically productive, 2OG conformation appears less likely for R735W AspH, because of the increased bulk of the W735 side chain compared with that of Q735, and the likely reduced ability of its indole NH to engage in polar and/or hydrogen bonding interactions with the 2OG C-5 carboxylate.

### 2OG derivatives can sustain catalysis of Traboulsi syndrome–associated AspH variants

Analysis of the R735Q and R735W AspH structures suggests that their 2OG binding pocket may be sufficiently spacious to enable productive binding of 2-oxoacids other than 2OG (Fig. 4). The distance of Mn to the Q735 side-chain carboxamide carbon in the R735Q AspH:2OG:Mn:hFX-EGFD1_86–124_-4S structure is ∼1.9 Å greater than that of Mn to the R735 side-chain guanidinium carbon in the wt AspH:2OG:Mn:hFX-EGFD1_86–124_-4S structure (Fig. 4), suggesting that 2OG derivatives with an extended carbon backbone may bind to R735Q AspH. This proposal is precedented by the observation that some human 2OG oxygenases can employ the human metabolite 2-oxoadipate (2OA) (79, 80), the carbon backbone of which bears an additional methylene group compared with 2OG, as a cosubstrate. Recent studies on the cosubstrate scope of wt AspH reveal that both natural and synthetic C3- and/or C4-substituted 2OG derivatives are efficient cosubstrates, enabling, at least in some cases, more efficient catalysis *in vitro* than 2OG itself (65, 81).

We investigated the ability of a set of 44 synthetic 2OG derivatives, including, at least, five natural products (*i*.*e*., 3-methyl-2OG (**1**) (82), 4-methyl-2OG (**3**) (83), 2OA (**10**) (84, 85), 2-oxopimelate (2OP, **11**) (86, 87), 2-oxosuberate (2OS, **12**) (86, 88, 89, 90, 91)), to sustain catalysis of isolated wt, G434V, R688Q, R735Q, and R735W AspH in the absence of 2OG using SPE–MS assays (Table 2, Table S2). The results reveal that 5 of the 44 tested 2OG derivatives retained >50% cosubstrate activity with wt AspH relative to 2OG (>95%; Table 2), including the natural products **1**, **3**, and 2OA, in accord with reported results on the cosubstrate scope of wt AspH obtained using a different hFX-derived substrate (65). Note that the absolute levels of wt AspH–catalyzed substrate oxidation with 2OG derivatives differ from those reported (65), possibly reflecting the different substrates and/or assay conditions employed.

**Table 2:** Effects of representative 2OG derivatives on catalysis by isolated recombinant human wt AspH and AspH variants (complete results are provided in Table S2)

Entry	2OG derivative^a^	wt AspH^b^^,^^c^	G434V AspH^b^	R688Q AspH^d^	R735Q AspH^e^	R735W AspH^e^
i		**>95%**	**>95%**	**35%** ± **3%**	**62%** ± **10%**	<1%
ii		**94%** ± **6%**	**90%** ± **6%**	**73%** ± **3%**	7% ± 2%	<1%
iii		10% ± 2%	<1%	<1%	<1%	<1%
iv		**44%** ± **13%**	5% ± 5%	<1%	<1%	<1%
v		<1%	<1%	<1%	<1%	<1%
vi^f^		**49%** ± **6%**	9% ± 6%	<1%	**48%** ± **3%**	<1%
vii		<1%	<1%	<1%	**49%** ± **5%**	<1%
viii		31% ± 8%	8% ± 5%	<1%	**90%** ± **13%**	<1%
ix		**61%** ± **13%**	16% ± 6%	<1%	**>95%**	**83%** ± **20%**
x		<1%	<1%	<1%	<1%	<1%
xi		**73%** ± **16%**	14% ± 6%	<1%	**>95%**	<5%
xii		22% ± 2%	<5%	<1%	**>95%**	8% ± 5%
xiii		<1%	<1%	<1%	**>95%**	<1%

Results are means of independent triplicates (n = 3; mean ± SD); 2OG derivatives showing >40% cosubstrate activity are provided in bold.

a Chiral 2OG derivatives were prepared as racemic mixtures unless noted otherwise (65).

b wt His_6_-AspH_315–758_ (0.1 μM) or G434V His_6_-AspH_315–758_ (0.1 μM) incubated with hFX-EGFD1_86–124_-4S (4.0 μM), LAA (100 μM), Fe(II) (20 μM), and 2OG derivative (500 μM) in buffer (25 mM Hepes, pH 7.5, 50 mM NaCl) for 15 min at 20 °C.

c The reactivity of wt AspH with 2OG derivatives is reported using hFX-derived substrate other than hFX-EGFD1_86–124_-4S (65).

d R688Q His_6_-AspH_315–758_ (0.2 μM) incubated with hFX-EGFD1_86–124_-4S (4.0 μM), LAA (400 μM), Fe(II) (100 μM), and 2OG derivative (50 μM) in buffer (25 mM Mes, pH 6.0) for 30 min at 20 °C.

e R735Q His_6_-AspH_315–758_ (0.2 μM) or R735W His_6_-AspH_315–758_ 0.2 μM) incubated with hFX-EGFD1_86–124_-4S (4.0 μM), LAA (400 μM), Fe(II) (100 μM), and 2OG derivative (2000 μM) in buffer (25 mM Mes, pH 6.0) for 3 h at 20 °C.

f Mixture of diastereomers, dr (*trans*:*cis*) = 1:1.

The SPE–MS assay results imply that the G434V and R688Q AspH cosubstrate scope is substantially narrower than that of wt AspH, with only the natural product 3-methyl-2OG (**1**) (82) showing turnover comparable to that observed for 2OG (Table 2, entry ii). The results indicate that **1** was a more efficient cosubstrate for R688Q AspH than 2OG, at least under the tested conditions. By contrast to **1**, neither 3-propyl-2OG (**2**) nor 4-methyl- or 4-propyl-2OG (**3** or **4**) were able to sustain catalysis of G434V and R688Q AspH, whereas both **2** and **3** sustained catalysis of wt AspH (Table 2, entries iii–v), indicating that subtle changes in the size and position of the 2OG substituent can have substantial effects on reactivity, as observed with other 2OG oxygenases (81, 92, 93). The results imply that altering both active site residues directly involved in (co)substrate binding and residues distal from the active site that likely affect substrate binding can determine the ability of the AspH cosubstrate to sustain catalysis.

The cosubstrate scope of the R735Q, R688Q, and G434V AspH variants differ substantially, including when using identical assay conditions (Table 2, Table S2). For example, 3-methyl-2OG (**1**) sustained catalysis by both G434V and R688Q AspH, but not R735Q AspH (Table 2, entry ii). This observation may reflect the reduced ability of **1** to adapt a catalytically active conformation in complex with R735Q AspH. 2OG derivatives **5** to **8**, which are conformationally more rigid than 2OG, appeared to sustain catalysis of R735Q AspH more efficiently than that of the other tested AspH variants and wt AspH, supporting the proposal that the *gauche* conformation 2OG was observed to adopt at the active site of R735Q AspH is catalytically inactive. Notably, the conformationally rigid and sterically bulky bicyclic 2OG derivative **6** only showed activity with the R735Q AspH variant (Table 2, entry vii).

Although 2OG was unable to sustain catalysis by R735W AspH even at relatively high concentrations (*i*.*e*., 2 mM), 2-bromo-4-carboxyphenylglyoxylic acid (**8**) served as a cosubstrate of R735W AspH, manifesting high levels of substrate oxidation (Table 2, entry ix). The observations that both 3-bromo-4-carboxyphenylglyoxylic acid (**9**) and 4-carboxyphenylglyoxylic acid (**7**) do not support catalysis by R735W AspH, the latter of which is a cosubstrate of wt AspH (65) that also showed activity with R735Q AspH (Table 2, entry viii), imply that the C-2 bromo substituent of **8** is important for productive catalysis with R735W AspH.

It is possible that the bromo substituent of **8** stabilizes binding at the R735W AspH active site through hydrophobic interactions with the region formed by the side chains of W625, M670, V676, and V727; this hydrophobic pocket is likely proximal to the C-2 phenyl-ring position of **8** and has been shown to accommodate the methyl group of 3-methyl-2OG (**1**) in a reported wt AspH:**1**:Mn:hFX-EGFD1_86–124_-4S structure (65). The combined results indicate that the crystallographically observed *gauche* conformation that 2OG adopts in complex with R735W AspH is likely not catalytically productive, unlike the antiperiplanar conformation adopted by 2OG in complex with wt AspH (Fig. 4). The relative arrangement of the two carboxylate groups of **8** in complex with R735W AspH mimics the likely catalytically productive antiperiplanar conformation of 2OG.

Incremental extension of the carbon backbone of 2OG by addition of methylene groups from 2OA (**10**) to 2OP (**11**) and 2OS (**12**) correlates with decreased wt AspH activity, with 2OS being inactive (Table 2, entries xi–xiii). By contrast, the levels of R735Q AspH activity with 2OG derivatives **10** to **12** were consistently high (>95%). Together with the observation that the 2OG derivatives **10** to **12** were inefficient cosubstrates for G434V, R688Q, and R735W AspH, the combined results reveal that 2OS selectively sustains R735Q AspH catalysis. This is of interest from a therapeutic perspective as the reactivity of 2OS with 2OG-utilizing enzymes other than AspH likely differs from that of 2OG. Notably, 2OS is a natural product and an intermediate in coenzyme B biosynthesis in methanogenic archaea (86, 88, 89); it has also been detected in human urine (90), possibly as a consequence of archaea in the gastrointestinal tract (94).

### 2-Oxoacids lacking the 2OG C-5 carboxylate can sustain catalysis of R735Q and R735W AspH

The observation that 2-bromo-4-carboxyphenylglyoxylic acid (**8**) efficiently sustains catalysis by R735W AspH suggested that structurally related naturally occurring hydrophobic 2-oxoacids may have the capacity to sustain catalysis of the R735W AspH variant (Table 2, entry ix). We thus extended our search for alternative cosubstrates of Traboulsi syndrome–associated AspH variants to hydrophobic 2-oxoacids bearing a single carboxylate group, including transamination products of proteinogenic amino acids abundant in cells, because they may stabilize binding to the active site of AspH variants through hydrophobic interactions and because they showed variable levels of activity with bacterial 2OG oxygenases (95, 96).

The results reveal that none of the 12 tested hydrophobic 2-oxoacids lacking the C-5 carboxylate of 2OG sustained catalysis by wt AspH as well as of the G434V and R688Q AspH variants (Table 3, Table S3). By contrast, 10 of the 12 tested 2-oxoacids lacking a second carboxylate sustained catalysis of both R735Q and R735W AspH, including **13**, **14**, **15**, and **16**, which are the transamination products of valine, methionine, isoleucine, and leucine, respectively (Table 3, entries ii–v). Notably, the levels of activity observed for R735Q and, in particular, R735W AspH with the 2-oxoacids **13**, **15**, and **16** are higher than those with the methionine transamination product **14** (Table 3), indicating that optimized 2-oxoacids might be identified that efficiently rescue the activity of Traboulsi syndrome–associated AspH variants. These results are important because amino acid transamination products are abundant in cells and because their concentrations may, at least in part, be affected by nutrition, implying that a diet rich in specific amino acids or their transamination products may possibly help mitigate Traboulsi syndrome–associated phenotypes.

**Table 3:** Effects of representative hydrophobic 2-oxoacids without a 2OG C5 carboxylate–equivalent group on catalysis by isolated recombinant human wt AspH and AspH variants (complete results are provided in Table S3)

Entry	2-Oxoacid	wt AspH^a^	G434V AspH^a^	R688Q AspH^b^	R735Q AspH^c^	R735W AspH^c^
i		**>95%**	**>95%**	**35%** ± **3%**	**62%** ± **10%**	<1%
ii		<1%	<1%	<1%	**>95%**	**>95%**
iii		<1%	<1%	<1%	**86%** ± **24%**	<5%
iv		<1%	<1%	<1%	**>95%**	**>95%**
v		<1%	<1%	<1%	**>95%**	**>95%**
vi		<1%	<1%	<1%	<1%	<1%
vii		<1%	<1%	<1%	**>95%**	**64%** ± **15%**
viii		<1%	<1%	<1%	21% ± 2%	**40%** ± **5%**
ix		<1%	<1%	<1%	**>95%**	**>95%**

Results are means of independent triplicates (n = 3; mean ± SD); 2-oxoacids showing >40% cosubstrate activity are provided in bold.

a wt His_6_-AspH_315–758_ (0.1 μM) or G434V His_6_-AspH_315–758_ (0.1 μM) incubated with hFX-EGFD1_86–124_-4S (4.0 μM), LAA (100 μM), Fe(II) (20 μM), and 2-oxoacid (500 μM) in buffer (25 mM Hepes, pH 7.5, 50 mM NaCl) for 15 min at 20 °C.

b R688Q His_6_-AspH_315–758_ (0.2 μM) incubated with hFX-EGFD1_86–124_-4S (4.0 μM), LAA (400 μM), Fe(II) (100 μM), and 2-oxoacid (50 μM) in buffer (25 mM Mes, pH 6.0) for 30 min at 20 °C.

c R735Q His_6_-AspH_315–758_ (0.2 μM) or R735W His_6_-AspH_315–758_ (0.2 μM) incubated with hFX-EGFD1_86–124_-4S (4.0 μM), LAA (400 μM), Fe(II) (100 μM), and 2-oxoacid (2000 μM) in buffer (25 mM Mes, pH 6.0) for 3 h at 20 °C.

By contrast to the amino acid transamination products **13** to **16**, neither the transamination products of phenylalanine (**17**; Table 3, entry vi) nor tyrosine (Table S3) sustained catalysis by any of the AspH variants. However, substituting the conformationally rigid phenyl group of **17** for a conformationally more flexible cyclohexyl group restored activity (Table 3; entry vii). Substituting the phenyl group of **17** for the sterically bulky *tert*-butyl group resulted in a reduced activity relative to the cyclohexyl-bearing 2-oxoacid **18** (Table 3; entry viii). By contrast with **17**, the transamination product of homophenylalanine **20** shows excellent levels of activity with both R735Q and R735W AspH (Table 3; entry ix).

The combined results with 2-oxoacids suggest that multiple factors determine whether 2-oxoacids can serve as cosubstrates of R735Q and R735W AspH, including their conformational flexibility and steric bulk (Table 3). Although the reasons why wt AspH as well as the G434V and R688Q AspH variants do not employ hydrophobic 2-oxoacids bearing a single carboxylate group as cosubstrates are unclear, their charged R735 guanidinium group may not sufficiently support the binding of hydrophobic 2-oxoacids at the active site. As for 2OG, the results indicate that the reactivity differences of AspH active site variants with other 2-oxoacids do likely not reflect variations in reaction conditions: R688Q AspH did not use 2-oxoacids with a single carboxylate although the conditions employed for testing were similar to those employed with R735Q and R735W AspH, which accept these types of 2-oxoacids (25 mM Mes, pH 6.0). R735W AspH also efficiently used multiple 2-oxoacids with a single carboxylate under conditions also employed to investigate the cosubstrate scope of wt AspH and G434V AspH (25 mM Hepes, pH 7.5, 50 mM NaCl), which both did not employ these types of 2-oxoacids (Table S3).

### Kinetic studies on the reactivity of AspH variants with 2-oxoacids

Kinetic studies with selected 2OG derivatives and hydrophobic 2-oxoacids bearing a single carboxylate group were performed using SPE–MS to quantify their efficiency in sustaining catalysis of the Traboulsi syndrome–associated AspH variants and to rank their therapeutic potential (Table 4, Table S4).

**Table 4:** Kinetic parameters of isolated recombinant human wt AspH and AspH variants for selected 2-oxoacids (complete results are provided in Table S4)

Entry	Cosubstrate^a^^,^^b^	AspH variant	$k_{\text{cat}}^{\text{app}}$ (s^-1^)	$K_{m}^{\text{app}}$ (μM)	$k_{\text{cat}}^{\text{app}}$/$K_{m}^{\text{app}}$ (mM^-1^ s^-1^)
i		wt (66)	0.31 ± 0.03	1.1 ± 0.4	280 ± 110
		G434V	0.69 ± 0.03	0.89 ± 0.13	770 ± 110
		R688Q	0.015 ± 0.001	0.81 ± 0.15	18 ± 4
		R735Q	0.006 ± 0.001	70 ± 20	0.09 ± 0.03
ii		wt	0.13 ± 0.01	1.6 ± 0.4	81 ± 17
		G434V	0.20 ± 0.01	2.3 ± 0.3	82 ± 9
		R688Q	0.03 ± 0.01	2.5 ± 0.4	11 ± 2
ciii		wt	0.07 ± 0.01	8.2 ± 0.6	8.9 ± 0.7
		R735Q	0.01 ± 0.01	170 ± 35	0.05 ± 0.02
iv		wt	0.11 ± 0.01	4.7 ± 0.7	24 ± 4
		R735Q	0.03 ± 0.01	85 ± 23	0.34 ± 0.10
v		R735Q	0.38 ± 0.03	550 ± 97	0.68 ± 0.13
vi		R735Q	0.72 ± 0.07	640 ± 160	1.1 ± 0.3
vii		R735Q	0.21 ± 0.02	300 ± 79	0.70 ± 0.20
		R735W	0.11 ± 0.01	236 ± 42	0.46 ± 0.09
viii		R735Q	0.16 ± 0.04	370 ± 110	0.44 ± 0.16
		R735W	0.10 ± 0.01	250 ± 37	0.42 ± 0.07
ix		R735Q	0.03 ± 0.01	270 ± 150	0.09 ± 0.06
		R735W	0.02 ± 0.01	210 ± 46	0.10 ± 0.03
x		R735Q	0.07 ± 0.01	87 ± 15	0.78 ± 0.14
		R735W	0.05 ± 0.01	390 ± 120	0.12 ± 0.04

Results are means of independent triplicates (n = 3; mean ± SD).

a Chiral 2OG derivatives were prepared as racemic mixtures as reported, unless noted otherwise (65).

b Kinetic parameters were determined using SPE–MS as described in the *Experimental procedures* section; SPE–MS analyses and Michaelis–Menten curves used to determine steady-state kinetic parameters are shown in Figures S15–S20.

c Mixture of diastereomers, dr (*trans*:*cis*) = 1:1.

Analysis of the wt AspH $k_{\text{cat}}^{\text{app}}$/$K_{m}^{\text{app}}$ values indicates that none of the 2OG derivatives are as efficient as 2OG: the wt AspH $k_{\text{cat}}^{\text{app}}$/$K_{m}^{\text{app}}$ value for 2OG was ∼3-fold greater than that for 3-methyl-2OG (**1**) (Table 4, entries i and ii). Previous results obtained using a different substrate and different conditions reported that the wt AspH $k_{\text{cat}}^{\text{app}}$/$K_{m}^{\text{app}}$ value for **1** was >2-fold greater than that for 2OG (65); note, however, that the $k_{\text{cat}}^{\text{app}}$/$K_{m}^{\text{app}}$ values are in the range of those reported (65). The wt AspH $k_{\text{cat}}^{\text{app}}$/$K_{m}^{\text{app}}$ value for the 2OG derivative **5** indicates that **5** was ∼30-fold less efficient in sustaining catalysis of wt AspH than 2OG; nonetheless, the observation that **5** acts as a cosubstrate is notable given its relatively conformationally rigid cyclic scaffold (Table 4, entry iii). The wt AspH $k_{\text{cat}}^{\text{app}}$/$K_{m}^{\text{app}}$ value for 2-bromo-4-carboxyphenylglyoxylic acid (**8**) was >350-fold less than that for 2OG, suggesting that wt AspH catalysis with 2OG derivatives bearing an aromatic scaffold is relatively inefficient (Table S4), in accord with reported results (65); note that the kinetic parameters of **8** were only determined for wt AspH, as variable results were obtained with R735Q and R735W AspH.

Analysis of the G434V AspH $k_{\text{cat}}^{\text{app}}$/$K_{m}^{\text{app}}$ values for 2OG and 3-methyl-2OG (**1**) suggests **1** is ∼9-fold less efficient in sustaining catalysis of G434V AspH than 2OG (Table 4, entries i and ii); the ninefold difference in the G434V AspH $k_{\text{cat}}^{\text{app}}$/$K_{m}^{\text{app}}$ values for 2OG and **1** was greater than that observed with wt AspH (Table 4, entries i and ii). By contrast, the R688Q AspH $k_{\text{cat}}^{\text{app}}$/$K_{m}^{\text{app}}$ values for 2OG and **1** were similar within experimental error and ∼8-fold lower than that observed for wt AspH with 2OG. The combined results indicate that the effects of substitutions in the AspH active site and TPR domain on the reactivity with 2OG derivatives vary with the 2OG substitution pattern; thus, there is likely scope to design improved 2OG derivatives that may fully rescue the activity of Traboulsi syndrome–associated AspH variants.

The R735Q AspH $k_{\text{cat}}^{\text{app}}$/$K_{m}^{\text{app}}$ values for 2OG and the cyclic 2OG derivative **5** were similar within experimental error; however, they were >3000- and ∼175-fold lower than those of wt AspH (Table 4, entries i and iii). Interestingly, the R735Q AspH $k_{\text{cat}}^{\text{app}}$/$K_{m}^{\text{app}}$ value for the lysine catabolite 2OA (**10**), which is present in human cells (84, 85), was ∼3-fold higher than that of 2OG, indicating a preference of R735Q AspH to employ 2OA as a cosubstrate rather than 2OG (Table 4, entry iv); however, the R735Q AspH $k_{\text{cat}}^{\text{app}}$/$K_{m}^{\text{app}}$ value for 2OA was ∼70-fold lower than that of wt AspH. Incrementally extending the 2OA carbon scaffold by methylene groups to give 2OP (**11**) and 2OS (**12**) resulted in ∼2- and ∼3-fold increases in the R735Q AspH $k_{\text{cat}}^{\text{app}}$/$K_{m}^{\text{app}}$ values, respectively, with the R735Q AspH $k_{\text{cat}}^{\text{app}}$/$K_{m}^{\text{app}}$ value for 2OS being ∼12-fold greater than that for 2OG (Table 4, entries v and vi). The observed increase in the R735Q AspH $k_{\text{cat}}^{\text{app}}$/$K_{m}^{\text{app}}$ value for 2OS is a result of increased $k_{\text{cat}}^{\text{app}}$ values. Although the R735Q AspH $k_{\text{cat}}^{\text{app}}$/$K_{m}^{\text{app}}$ value for 2OS was ∼250-fold lower than that of wt AspH for 2OG, the results clearly reveal the potential of (naturally occurring) 2OG derivatives for, at least, partially rescuing the activity of Traboulsi syndrome–associated AspH variants.

Both the R735Q and R735W AspH $k_{\text{cat}}^{\text{app}}$/$K_{m}^{\text{app}}$ values for the tested 2-oxoacids with a single carboxylate are in the range of those obtained with R735Q AspH for 2OA and 2OS (Table 4, entries vii–x; Table S4). Comparison of the R735Q AspH $k_{\text{cat}}^{\text{app}}$/$K_{m}^{\text{app}}$ values for the 2-oxoacids **18** and **20** indicates that increasing the steric bulk and/or conformational rigidity of the hydrophobic 2-oxoacid substituent reduces the catalytic efficiency by ∼10-fold (Table 4, entries ix and x). The combined observations indicate that the reactivity of abundant hydrophobic amino acid transamination products with pathogenic AspH variants differs from that of 2OG derivatives and that these types of 2-oxoacids have the potential to sustain catalysis of Traboulsi syndrome–associated AspH variants in cells.

### Conformations of 2OG derivatives in complex with pathogenic AspH variants differ from those of 2OG

The R688Q and R735Q AspH variants were crystallized in the presence of selected 2OG derivatives, Mn(II), and hFX-EGFD1_86–124_-4S (Fig. S1*C*) (52) to inform on the conformation of the 2OG derivatives in complex with them. Crystal structures of the R688Q AspH:3-methyl-2OG (**1**):Mn:hFX-EGFD1_86–124_-4S and the R735Q AspH:2OS (**12**):Mn:hFX-EGFD1_86–124_-4S complexes were obtained (*P*2_1_2_1_2_1_ space group; 1.85 and 1.70 Å resolution, respectively; Figs. S21-S27, Table S5), and solved by molecular replacement using a reported structure as a search model (PDB ID: 6YYW (65)).

**Figure 5:**
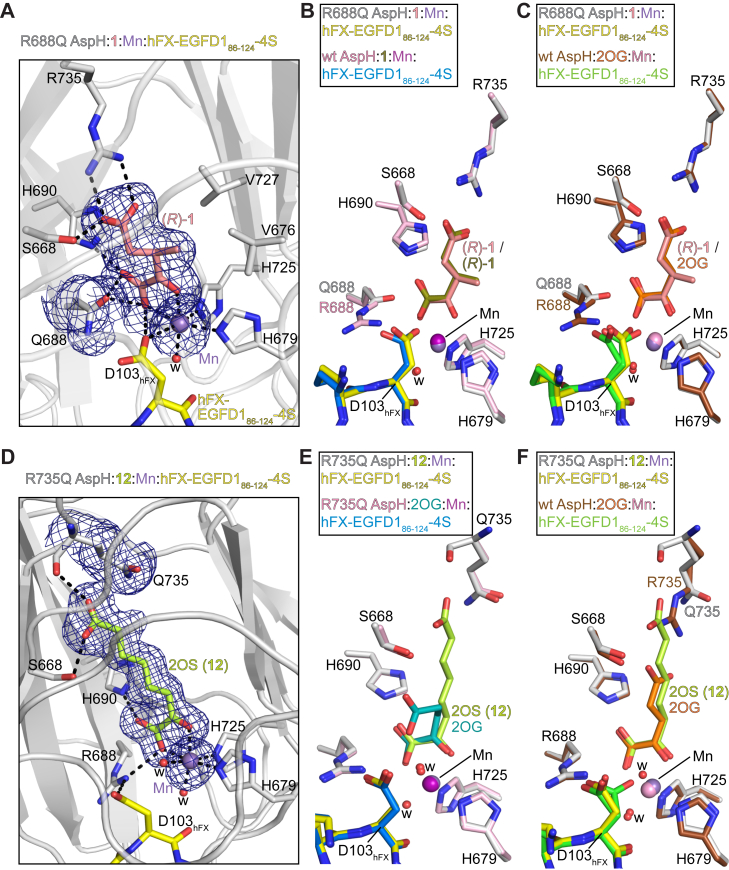
**3-Methyl-2OG and 2OS bind to the Traboulsi syndrome–associated R688Q and R735QAspH variants in a similar manner as 2OG binds to wt AspH**. Colors: *gray*: R688Q and R735Q AspH_315–758_; *lavender*: Mn; *yellow*: carbon backbone of the hFX-EGFD1_86–124_-4S peptide (Fig. S1*C*); *red*: oxygen; *blue*: nitrogen; and w: water. *A*, representative Polder omit electron density maps contoured to 3.0σ around (3*R*)-3-methyl-2OG (**1**; *salmon*), Mn (*lavender*), and Q688 in the R688Q AspH:**1**:Mn:hFX-EGFD1_86–124_-4S structure; (*B* and *C*) superimposition of active site views of the R688Q AspH:**1**:Mn:hFX-EGFD1_86–124_-4S structure with (*B*) the reported wt AspH:**1**:Mn:hFX-EGFD1_86–124_-4S (colors: *light pink*: wt AspH; *olive*: (*R*)-**1**; *purple*: Mn; *blue*: carbon backbone of hFX-EGFD1_86–124_-4S; PDB ID: 6YYX (65)) and (*C*) the improved wt AspH:2OG:Mn:hFX-EGFD1_86–124_-4S (colors: *brown*: wt AspH; *orange*: 2OG; *pink*: Mn; *light green*: carbon backbone of hFX-EGFD1_86–124_-4S) structures. *D*, representative Polder omit electron density maps contoured to 3.0σ around 2-oxosuberate (2OS; **12**; *lemon green*), Mn (*lavender*), and Q735 in the R735Q AspH:2OS:Mn:hFX-EGFD1_86–124_-4S structure; (*E* and *F*) superimposition of active site views of the R735Q AspH:2OS:Mn:hFX-EGFD1_86–124_-4S structure with (*E*) the R735Q AspH:2OG:Mn:hFX-EGFD1_86–124_-4S (colors: *light pink*: R735Q AspH; *teal*: 2OG; *purple*: Mn; and *blue*: carbon backbone of hFX-EGFD1_86–124_-4S) and (*F*) the improved wt AspH:2OG:Mn:hFX-EGFD1_86–124_-4S (colors: *brown*: wt AspH; *orange*: 2OG; *pink*: Mn; *light green*: carbon backbone of hFX-EGFD1_86–124_-4S) structures. AspH, aspartate/asparagine-β-hydroxylase; EGFD, epidermal growth factor–like domain; hFX, human coagulation factor X; 2OG, 2-oxoglutarate; 2OS, 2-oxosuberate.

Analysis of the electron density maps of the R688Q AspH:**1**:Mn:hFX-EGFD1_86–124_-4S structure validates substitution of R688 for a glutamine residue (Fig. 5*A*), consistent with MS and sequencing analyses (Fig. S2). Superimposition of the R688Q AspH:**1**:Mn:hFX-EGFD1_86–124_-4S and the reported wt AspH:**1**:Mn:hFX-EGFD1_86–124_-4S (PDB ID: 6YYX (65)) structures shows that the R688Q variation neither affects the overall fold (RMSD ∼0.18 Å), nor the conformation of hFX-EGFD1_86–124_-4S (RMSD ∼0.20 Å; Fig. S22). Similarly, comparison with the wt AspH:2OG:Mn:hFX-EGFD1_86–124_-4S structure reveals that the substitution of 2OG for **1** together with the R688Q substitution does not affect the overall fold (RMSD ∼0.18 Å) and the conformation of hFX-EGFD1_86–124_-4S (RMSD ∼0.16 Å; Fig. S23).

Consistent with the reported wt AspH:**1**:Mn:hFX-EGFD1_86–124_-4S (PDB ID: 6YYX (65)) structure, analysis of the electron density map reveals that the (3*R*)-enantiomer of 3-methyl-2OG (**1**) selectively crystallizes from a racemic mixture of **1** with R688Q AspH, Mn, and hFX-EGFD1_86–124_-4S, suggesting that hydrophobic interactions of the methyl group of **1** with proximate hydrophobic side chains (*e*.*g*., W625, M670, V676, V727) stabilize its binding (Fig. S24) (65). The conformation of **1** in both the R688Q AspH:**1**:Mn:hFX-EGFD1_86–124_-4S and the reported wt AspH:**1**:Mn:hFX-EGFD1_86–124_-4S (PDB ID: 6YYX (65)) structures is apparently identical to that of 2OG observed in the wt AspH:2OG:Mn:hFX-EGFD1_86–124_-4S structure (Fig. 5, *A*–*C*); **1** is positioned to interact with R688Q AspH and Mn in an identical manner as 2OG/**1** with wt AspH and Mn.

The side-chain primary amide of Q688 is positioned to interact with the C-1 carboxylate of **1** in the R688Q AspH:**1**:Mn:hFX-EGFD1_86–124_-4S structure (2.6 and 2.7 Å resolution), but not with the substrate D103_hFX_ side-chain carboxylate, as observed for the guanidinium group of R688 in the reported wt AspH:**1**:Mn:hFX-EGFD1_86–124_-4S (PDB ID: 6YYX (65)) structure (Fig. 5, *A*–*C*). The combined observations suggest that the R688Q substitution does not compromise 2OG or 2OG derivative binding to the active site, a proposal consistent with kinetic studies revealing that the R688Q AspH $K_{m}^{\text{app}}$ values for both 2OG and **1** are in the range of those obtained with wt AspH (Tables 1 and 4).

Interestingly, the D103_hFX_ side-chain carboxylate of hFX-EGFD1_86–124_-4S adopts a single conformation in the R688Q AspH:**1**:Mn:hFX-EGFD1_86–124_-4S structure, in which it coordinates to Mn (3.0 Å; Fig. 5, *A*–*C*), as observed in the reported wt AspH:**1**:Mn:hFX-EGFD1_86–124_-4S structure (PDB ID: 6YYX (65)). Hence, the reduced catalytic efficiency of the R688Q AspH variant compared with wt AspH (Fig. 1; Tables 1 and 4) may in part be a result of the hFX-EGFD1_86–124_-4S D103_hFX_ side-chain carboxylate adopting a catalytically unproductive conformation by coordinating to the active site metal, thus possibly impairing O_2_ binding to this coordination site of the metal cofactor. R688 may contribute toward stabilizing the catalytically active conformation of the D103_hFX_ side chain by interacting with its carboxylate group, as observed in the wt AspH:2OG:Mn:hFX-EGFD1_86–124_-4S structure (Fig. 5, *A*–*C*), enhancing the probability of O_2_ to bind to the active site metal and positioning D103_hFX_ in a manner to undergo β-oxidation. Note that the conformation of the D103_hFX_ side chain may differ when using full-length AspH substrates and/or full-length AspH and when performing crystallizations in the presence of Fe(II) instead of Mn(II).

Superimposition of the R735Q AspH:2OS:Mn:hFX-EGFD1_86–124_-4S and the R735Q AspH:2OG:Mn:hFX-EGFD1_86–124_-4S structures reveals very similar overall AspH fold (RMSD ∼0.17 Å) and hFX-EGFD1_86–124_-4S conformations (RMSD ∼0.13 Å; Fig. S26). Electron density analysis indicates that the conformations of the C-3, C-4, and C-5 atoms of 2OS in the R735Q AspH:2OS:Mn:hFX-EGFD1_86–124_-4S structure substantially differ from that of the 2OG C-3, C-4, and C-5 atoms in the R735Q AspH:2OG:Mn:hFX-EGFD1_86–124_-4S structure, whereas they are similar (but not identical) to that of the 2OG C-3, C-4, and C-5 atoms in the wt AspH:2OG:Mn:hFX-EGFD1_86–124_-4S structure (Fig. 5, *E* and *F*). By contrast, the conformation of the metal-binding 2-oxoacid motif of 2OG and 2OS is identical in the three AspH structures. The dihedral angle formed by the C-2/C-3/C-4/C-5 atoms of 2OS in the R735Q AspH:2OS:Mn:hFX-EGFD1_86–124_-4S structure is ∼179°, indicative of an antiperiplanar conformation around the 2OS C-3/C-4 bond, similar to the dihedral angle of ∼174° around the 2OG C-3/C-4 bond observed in the wt AspH:2OG:Mn:hFX-EGFD1_86–124_-4S structure (Fig. 5*F*); the combined evidence implies that the antiperiplanar conformation of 2OS in complex with R735Q AspH is likely catalytically productive (Table 2).

Notably, superimposition of the R735Q AspH:2OS:Mn:hFX-EGFD1_86–124_-4S and the wt AspH:2OG:Mn:hFX-EGFD1_86–124_-4S structures reveals that the C8 carboxylate group of 2OS is positioned close to the R735 guanidinium group in the wt AspH complex structure (Fig. 5*F*), in a manner to interact with the hydroxyl group of S668 (2.5 Å) and the main-chain carboxamide group of Q735 (2.8 Å). Interestingly, a second 2OS molecule was bound on the surface of R735Q AspH, the mechanistic implications (if any) of which are unclear (Fig. S25). Overall, the combined results provide proof of concept that 2OG derivatives have the potential to alter catalysis of Traboulsi syndrome–associated AspH active site variants in a manner to, at least in parts, compensate for their reduced catalytic activity.

## Discussion

Traboulsi syndrome–linked nonsense terminations in *ASPH* resulting in C-terminally truncated AspH variants lacking the 2OG oxygenase domain imply that the associated phenotypes are a result of impaired AspH catalysis (12, 19, 21, 23, 25, 63). However, this proposal had not been validated at a biochemical level, which is important given the 2OG oxygenase domains of human 2OG oxygenases may have functions other than catalyzing oxidation reactions (97, 98, 99); for example, the AspH 2OG oxygenase and TPR domains may have a role in catalyzing EGFD disulfide isomerization (52). *ASPH* also encodes for multiple C-terminally truncated isoforms lacking the catalytic 2OG oxygenase domain, *e.g.*, junctate, junctin, and humbug, that have roles *inter alia* in Ca(II) binding (27, 28, 29, 30), dysregulated levels of which could potentially result in the phenotypes associated with Traboulsi syndrome.

Our results provide clear biochemical and crystallographic evidence that the pathogenic R688Q, R735Q, and R735W AspH variants are catalytically less active than wt AspH while retaining the same fold as wt AspH (Table 1 and Fig. 4), supporting the proposal that the phenotypes associated with Traboulsi syndrome are due to a reduction in AspH oxygenase catalysis. Note that in addition to altering catalytic efficiency, it is possible that the mutations affect thermal- and/or proteolytic-stability in cells. The observation that none of the tested AspH variants catalyzes substantial levels of substrate-uncoupled 2-oxoacid turnover *in vitro* (Fig. S4), as is the case for wt AspH (52), implies that the Traboulsi syndrome–associated substitutions may not exert pathogenicity by directly regulating (local) metabolism in cells, including in a manner related to substrate-coupled and -uncoupled turnover of 2OG as proposed for FIH (62, 100, 101, 102). This proposal is consistent with the apparent absence of Traboulsi syndrome–related phenotypes in mice lacking FIH (100, 102).

The mechanism(s) by which reduced levels of AspH catalysis cause the phenotypes associated with Traboulsi syndrome are unclear, including because >100 human proteins contain EGFDs with the consensus sequence required for productive AspH catalysis (23, 40). Given that wt AspH acts on multiple EGFDs, the specific set of EGFDs involved in Traboulsi syndrome is hard to define, with competition between substrates, as proposed for FIH, being a possible factor (61). AspH may also catalyze other reactions in addition to EGFD hydroxylations, including, *e.g.*, EGFD disulfide isomerizations (47); it is thus of interest to investigate whether Traboulsi syndrome–associated variations in the AspH active site have an impact on the EGFD disulfide connectivity, although this is challenging to do in cells.

Connecting the biochemistry of AspH with its physiological roles in health and disease is thus challenging and may require *in vivo* investigations beyond the characterization of individual reactions, as may be the case for other 2OG oxygenases acting on multiple substrates, including FIH (55, 59, 60, 61, 62). The observation that both latent-transforming growth factor β-binding proteins (LTBPs) (39, 103) and FBNs are AspH substrates is of clinical interest (38, 39, 40), because they are critical components of the ciliary zonule, the extracellular fiber system that centers and suspends the lens in the eye (104, 105). It is possible that altered EGFD hydroxylation levels in LTBPs and/or FBNs cause the ocular phenotypes associated with Traboulsi syndrome. This proposal is consistent with observations that mutations in the human *LTBP2* gene can cause *inter* *alia* ectopia lentis (106, 107, 108, 109, 110) and that LTBP2-deficient mice develop ectopia lentis (111, 112). In this regard, it is also of interest that some symptoms of Traboulsi syndrome overlap with those of the Marfan syndrome (OMIM: 154700) (19, 63, 113, 114, 115).

Marfan syndrome is a connective tissue disorder caused by SNPs in *FBN1* that encodes for FBN1 (116, 117). 43 EGFDs in FBN1 bear the consensus sequence required for productive AspH catalysis (117); note there is evidence that the levels of individual EGFD hydroxylation in FBN1 vary from none to complete (38, 39, 40). The FBN1 EGFDs enable polymerization to form microfibrils that help center the lens in the eye; disrupting FBN1 polymerization can result in ectopia lentis (51, 104, 118). Some Marfan syndrome–associated SNPs occurring in the EGFD-encoding genomic region of *FBN1* cause ectopia lentis, including those that alter the number of cysteine residues in EGFDs (119, 120, 121). Given that AspH requires an EGFD C3–C4 disulfide connectivity for catalysis *in vitro* (40, 52), altering the balance of disulfide connectivity in FBN1 EGFDs may affect AspH catalysis. Interestingly, *FBN1* SNPs that affect EGFD residues other than cysteines that are essential for AspH catalysis have also been reported to result in ectopia lentis (119). Note, however, that alterations in genes encoding for proteins that are not substrates of AspH also cause ectopia lentis (122, 123, 124).

The proposal that reduced levels of FBN1 EGFD hydroxylation can cause Traboulsi and Marfan syndrome–associated connective tissue defects raises the question of whether reported small-molecule inhibitors of AspH (65, 76, 77, 125, 126, 127, 128, 129) could trigger similar connective tissue defects, a possibility that deserves attention because increased AspH levels on the surface of cancer cells have been correlated with worse clinical prognosis, rendering AspH a potential target for cancer treatment (130, 131, 132, 133). Small-molecule AspH inhibitors have shown potential in both cellular and animal studies to suppress cancer progression related to AspH upregulation (126, 127, 132, 134, 135); however, the long-term safety of these inhibitors has not yet been investigated.

Some phenotypes associated with Traboulsi syndrome resemble those of Duane retraction syndrome 1 (DURS1; OMIM: 126800) (136, 137), a rare congenital disease linked to duplications in the 8q12 and 8q13 regions of chromosome 8. The DURS1-associated cytogenetic abnormalities typically affect multiple genes, including, at least in some cases, *ASPH*, which is located on chromosome 8 (8q12.3). DURS1-associated phenotypes include, *e.g.*, globe retraction, strabismus, craniofacial dysmorphism, and vesicoureteral reflux (138, 139, 140, 141, 142, 143). Although it is difficult to correlate the DURS1-associated phenotypes to a specific gene because of its genetic heterogeneity, the overlapping phenotypes of Traboulsi syndrome and DURS1 are striking and the role of *ASPH* in DURS1 should be investigated.

The phenotype(s) associated with the G434V AspH variant are, unlike those of the R688Q, R735Q, and R735W AspH variants, not unequivocally linked to Traboulsi syndrome (64), which may reflect knowledge that the G434V variation is not located in the active site, but in the TPR domain, which is N-terminal to the AspH oxygenase domain and which has a role in substrate binding (Fig. 1) (52). Though care should be taken in the interpretation of kinetic parameters in the context of the complex AspH reaction, including because our previous results showed that the ambient O_2_ concentration is not saturating for wt AspH (47), our studies indicate that the G434V AspH variant is catalytically more active than wt AspH (Table 1). This result is of interest because it suggests that *ASPH* mutations may be engineered to investigate the consequences of enhanced AspH activity in cells and animal model studies for functional assignment. Note, however, that we employed an N-terminally truncated AspH construct and a synthetic substrate; it is possible that the reactivity of full-length G434V AspH with folded full-length EGFDs is less efficient in cells than that of wt AspH and that variations in TPR residues may affect the ability of AspH to bind to specific subsets of substrates.

The observation that SNPs in the genomic region of *ASPH* encoding for the TPR domain can affect catalysis extends to 2OG oxygenases other than AspH. For example, X-linked Kabuki syndrome-2 (OMIM: 300867) is caused by mutations in the human *UTX* gene and is associated with severe developmental defects (144, 145, 146). *UTX* encodes for the 2OG-dependent Jumonji-C domain histone *N*^ε^-methyl lysine demethylase 6A (UTX), which catalyzes demethylation of dimethylated and trimethylated *N*^ε^-lysine residue 27 of histone H3 (147, 148, 149). Like AspH, UTX bears an N-terminal TPR domain, which is reported to be involved in protein substrate binding (150). Notably, clinically observed SNPs in *UTX*, which alter single residues in the UTX TPR domain, are reported to cause Kabuki syndrome-2 (151, 152). In addition, cancer-associated SNPs in *UTX* regions encoding for TPR residues are reported to alter the ability of the UTX TPR to bind to proteins (153, 154). Thus, the combined evidence indicates that TPR domains of 2OG oxygenases and, by implication, other proteins may be hotspots for pathogenic mutations, likely because of their importance in substrate binding (155).

Our analyses show that all the tested Traboulsi syndrome–associated AspH variants retain at least basal levels of oxygenase activity with 2-oxoacids present in human cells and reveal that both the catalytic efficiency and cosubstrate scope of the tested AspH variants vary with respect to both the location (*i*.*e*., R688 or R735) and the type of variation (*i*.*e*., R735Q *versus* R735W) (Table 1, Table 2, Table 3, Table 4). The latter observation indicates that the Traboulsi syndrome–associated phenotype(s) and their severity could differ, including because heterozygous *ASPH* mutations can cause Traboulsi syndrome (23). However, it may be difficult to establish a clear genotype–phenotype correlation, including because cellular cosubstrate availability and AspH variant cosubstrate selectivity may vary on a case-to-case basis, potentially including in a diet-dependent manner, and because AspH-related 2OG oxygenases may compensate for the associated loss in AspH activity and catalyze EGFD hydroxylation, *e.g.*, aspartate β-hydroxylase domain–containing proteins 1 and 2, the substrate(s) and biological roles of which are currently unknown (66, 156, 157).

The observation that the R735Q AspH variant employs 2OG to sustain substrate oxidation, albeit at substantially lower levels than wt AspH (Table 4), contrasts with the reported cosubstrate scope of certain pathogenic active site variants of the 2OG oxygenase phytanoyl-CoA 2-hydroxylase (PAHX), which catalyzes the α-oxidation of chlorophyll-derived phytanic acid. Clinically observed PAHX variants are associated with Refsum disease (OMIM: 266500) (158, 159, 160), including the R275Q/W PAHX variants, which correspond to the R735Q/W variations in AspH (78, 160). By contrast to R735Q AspH, the R275Q/W PAHX variants are reported to be unable to employ 2OG as a cosubstrate *in vitro* (161). Although the R735Q AspH variant is active with 2OG, its $K_{m}^{\text{app}}$ value for Fe(II) was ∼3-fold higher than that of wt AspH (Table 1, entry ii), suggesting that impaired 2OG binding reduces the stability of the ternary AspH:Fe(II):2OG complex. Thus, it is, at least in principle, possible that the Traboulsi syndrome–associated variations in the AspH active site alter the consensus mechanism for 2OG oxygenases, *i.e.*, they perturb the preferred ordered sequential binding of 2OG, then substrate, and then O_2_ to the active site (162, 163).

To date, AspH is unique amongst identified human 2OG–dependent protein hydroxylases, because its Fe(II) cofactor is coordinated by only two protein-bound histidine residues (*i*.*e*., H679 and H725 (52, 66)) that form an HXG…H motif typically associated with 2OG-dependent halogenases (164, 165), whereas all other characterized 2OG-dependent human protein hydroxylases bear an Fe(II)-binding triad (*i*.*e*., HXD/E…H) (166). Refsum disease–associated variations in Fe(II) binding ligands of PHAX (*i*.*e*., H175R and D177A/G) (160, 167) were shown to be inactive *in vitro* (161), which, together with the observations that active site variants of the human 2OG–dependent protein hydroxylase FIH with two protein-bound Fe(II) ligands retain activity (168, 169), suggests that the implications of active site variations in 2OG oxygenases on catalysis have to be considered on a case-to-case basis. For example, it will be of interest to investigate whether reported pathogenic variations in KDM6B located in the Fe(II) binding site have the potential to alter Fe(II) binding (170).

The biochemical and biophysical results on the investigated Traboulsi syndrome–associated AspH variants expand knowledge on the active site requirements for productive catalysis of 2OG oxygenases. Our combined results suggest that the *gauche* conformation, which 2OG adopts in complex with R735Q/W AspH, at least when performing crystallizations in the presence of Mn(II), does not support catalysis (Fig. 4). The conformation of 2OG at the R735Q AspH active site may be dynamic, with an antiperiplanar conformation similar to that observed for 2OG in complex with wt AspH likely enabling productive substrate hydroxylation. This proposal is of relevance with respect to 2OG oxygenases, which catalyze bifurcating reactions (171, 172, 173, 174), because it raises the possibility that varying 2OG conformations at the active site may determine the reaction pathway (93, 175, 176, 177, 178).

The combined results reveal the ability of cellular abundant 2-oxoacids other than 2OG, including amino acid transamination products, to, at least, partially rescue the activity of Traboulsi syndrome–associated AspH variants *in vitro* (Table 2, Table 3, Table 4). It is interesting that the pathogenic AspH variants behave differently both with respect to their use of 2OG and other 2-oxoacids as cosubstrates (Table 2, Table 3, Table 4) and with respect to the crystallographically observed binding modes (Figs. 4 and 5). Thus, given the common symptoms of Traboulsi syndrome, the available evidence suggests that the disease is a result of impaired catalysis/EGFD hydroxylation as catalyzed by AspH.

The observation that cellular abundant 2-oxoacids sustain catalysis of pathogenic AspH variants is precedented by work on the 2-oxoacid cosubstrate scope of the Refsum disease–associated R275Q/W PAHX variants (160), corresponding to the R735Q/W variations in AspH (78), revealing their inactivity with 2OG can be partially rescued by hydrophobic amino acid transamination products lacking a 2OG C-5 carboxylate equivalent group (179). However, as observed for amino acid transamination products and 2OG derivatives with the R735Q and R735W AspH variants (Table 2, Table 3, Table 4), 2-oxoacids did not restore the activity of pathogenic PAHX variant to the level of that observed for wt PAHX with 2OG (179). The combined evidence implies that single-residue variations in the active site of 2OG oxygenases can have a pronounced impact on the reactivity of 2OG oxygenases with 2-oxoacids, regardless as to whether the 2OG oxygenase catalyzes protein hydroxylations (as AspH; Tables 1 and 2), small-molecule oxidations (as PHAX (179)), or protein *N*^ε^-methyl lysine demethylations (as the human Jumonji-C *N*^ε^-methyl lysine demethylase 4A (180)), and raise the possibility that 2OG oxygenases may (preferentially) employ 2-oxoacids other than 2OG in cells, as precedented by 4-hydroxyphenylpyruvate dioxygenase, which belongs to the 2OG oxygenase superfamily and which catalyzes conversion of HPPA into homogentisate (181).

Although it remains to be evaluated whether 2OG derivatives (or precursors of them) are useful in a clinical setting to treat the Traboulsi syndrome–associated phenotypes, it should be noted that a diet enriched or devoid of particular ingredients is a frequently used noninvasive strategy to manage metabolic diseases in the clinic, including, *e.g.*, a diet low in phytanic acid for individuals suffering from Refsum disease (182) and a diet strictly controlled for amino acid composition for individuals suffering from maple syrup disease (OMIM: 248600) (183). Amino acid transamination products and 2OG derivatives, including 3-methyl-2OG (**1**) (82), 2OA (**10**) (84, 85), and 2OS (**12**) (86, 88, 89), are natural products present in cells and human nutrition (82, 90); thus, although 2-oxoacids can react with enzymes other than AspH, cells may tolerate their presence to a certain concentration. Although lens dislocation may not be reversible without surgical intervention in adults, it is possible that a diet rich in amino acid transamination products and/or 2OG derivatives (or precursors of them) may be of use to mitigate other phenotypes of those patients who bear *ASPH* mutations corresponding to pathogenic AspH variations, given there is no effective treatment for Traboulsi syndrome.

## Experimental procedures

### AspH production and purification

An N-terminally truncated construct of N-terminally His_6_-tagged human wt AspH (His_6_-AspH_315–758_) was produced and purified as reported (69); AspH variants were produced and purified accordingly. The N-terminal His_6_-tag of wt His_6_-AspH_315–758_ was cleaved as reported (69). Proteins were >95% pure by SDS-PAGE and MS analyses (Figs. S2 and S3); they were stored at −80 °C.

Site-directed mutagenesis to obtain plasmid DNA coding for AspH variants was carried out using the reported N-terminally His_6_-tagged human wt AspH construct (His_6_-AspH_315–758_) (52) and the Q5 Site-Directed Mutagenesis Kit (New England Biolabs) according to the manufacturer’s instructions. Primers used were G434V AspH (forward primer: 5′-GTTCATATGAGAGGTTCCCTGCTTACCCTG-3′, reverse primer: 5′-CTCATATGAACTAGAAATTGTTGCCTGTCTGAGCGACGCTTC-3′), R688Q AspH (forward primer: 5′-CTCCAGATGCACCTGGGCTTG-3′, reverse primer: 5′-GTGCATCTGGAGCCTGCAGT-3′), R735Q AspH (forward primer: 5′-CTTTCCAGCTGATATTCATCGTGG-3′, reverse primer: 5′-GAATATCAGCTGGAAAGATGAGG-3′), R735W AspH (forward primer: 5′-GCCTCATCTTTCTGGCTGATATTCATCGTGGATG-3′, reverse primer: 5′-GAATATCAGCCAGAAAGATGAGGCATCCTGCCATAC-3′).

### AspH substrate

The hFX-EGFD1_86–124_-4S peptide (Fig. S1*C*) (52), the sequence of which is based on that of EGFD1 from the characterized AspH substrate hFX (35, 36). hFX-EGFD1_86–124_-4S was used in this work as an AspH substrate for turnover studies and crystallographic analyses; it was synthesized as a C-terminal amide by solid-phase peptide synthesis and purified by GL Biochem (Shanghai) Ltd.

### AspH assays

SPE–MS AspH assays were performed as reported using human wt His_6_-AspH_315–758_ or variants thereof (40, 47, 66, 69); assay buffers and cosubstrate/cofactor stock solutions were freshly prepared from commercially sourced solids. hFX-EGFD1_86–124_-4S (Fig. S1*C*) (52) was used as the substrate. Assay conditions are given in Tables 2 and 3, Tables S2–S4, and in the Supporting Information for kinetic studies (Figs. S5–S7 and S15–S20).

### Crystallography

wt AspH_315–758_ and N-terminally His_6_-tagged AspH_315–758_ variants were used for crystallography. AspH (374 μM) was incubated with Mn(II) (1 mM), 2OG (10 mM), and hFX-EGFD1_86–124_-4S (0.8 mM) in buffer (50 mM Hepes, pH 7.5, 150 mM NaCl) at 4 °C for 15 min and centrifuged using a MicroCL 21R centrifuge (Thermo Fisher Scientific; 18,800*g*, 4 °C, 5 min) prior to setting up sitting-drop or hanging-drop crystallizations in the presence of wt AspH crystal seeds in 24-well plates (Hampton Research). Three microliter drops (1 μl of precipitant with 2 μl of protein solution) and 500 μl precipitant were used per well. Typically, the precipitant pH (0.1 M Bis–Tris propane, pH 6.5–8.5 in 0.5 pH increments, vertical axis) and PEG concentration (PEG 3350, 18–22 in 1% increments, horizontal axis) were varied in the 24-well plates. Crystallizations were performed at 4 °C. AspH crystals were soaked with a mixture containing 10 mM 2OG, 30% v/v PEG 400, and 70% v/v precipitant for 10 min immediately before flash freezing in liquid N_2_.

To obtain crystal seeds of wt AspH, a mixture of wt AspH_315–758_ (374 μM), Mn(II) (1 mM), 2OG (2 mM), and hFX-EGFD1_86–124_-4S (0.8 mM) in buffer (50 mM Hepes, pH 7.5, 150 mM NaCl) was incubated at 4 °C for 15 min prior to centrifugation using a MicroCL 21R centrifuge (Thermo Fisher Scientific; 18,800*g*, 4 °C, 5 min). Crystallizations were performed in 96-well, three-subwell, low-profile SWISSCI 3 lens crystallization plates using a Mosquito LCP (SPT Labtech) dispensing robot and the commercial PACT Premier crystallization screen (Molecular Dimensions). Crystals were grown using the sitting-drop vapor diffusion method at 4 °C in 300 nl sitting drops with 2:1, 1:1, or 1:2 sample:precipitant solution ratios. One well of the crystallization plate containing crystals was transferred into a SeedBead HR2-320 Eppendorf together with all the reservoir solutions of that well (∼22 μl). The crystal seeds were prepared by 10 iterations of vortexing (30 s) and incubations on ice (30 s).

Diffraction data for the AspH single crystals were collected at 100 K using synchrotron radiation at the Diamond Light Source (beamline: I03). The data were indexed, integrated, and scaled using the Xia2 pipeline (184). Structures were solved by molecular replacement with Phaser (185), using a wt AspH structure (PDB ID: 6YYW (65)) as a search model. Structures were iteratively refined using PHENIX (186) and manual model building using COOT (187).

## Data availability

Crystal structure data for wt AspH_315–758_ and the R688Q, R735Q, and R735W His_6_-AspH_315–758_ variants complexed to Mn, 2OG, or a 2OG derivative ((3*R*)-3-methyl-2OG, **1**; 2OS, **12**), and the hFX-derived hFX-EGFD1_86–124_-4S peptide (Fig. S1*C*) are deposited in the PDB with PDB accession codes: 8RE9 (wt AspH:2OG:Mn:hFX-EGFD1_86–124_-4S), 8RE6 (R735Q AspH:2OG:Mn:hFX-EGFD1_86–124_-4S), 8RE7 (R735W AspH:2OG:Mn:hFX-EGFD1_86–124_-4S), 8RE8 (R688Q AspH:**1**:Mn:hFX-EGFD1_86–124_-4S), and 8RE5 (R735Q AspH:2OS:Mn:hFX-EGFD1_86–124_-4S).

## Supporting information

This article contains supporting information (35, 36, 40, 47, 52, 65, 66, 69, 188, 189).

## Conflict of interests

The authors declare that they have no conflicts of interest with the contents of this article.

## References

[1] Patel N., Khan A.O., Mansour A., Mohamed J.Y., Al-Assiri A., Haddad R. (2014). Mutations in ASPH cause facial dysmorphism, lens dislocation, anterior-segment abnormalities, and spontaneous filtering blebs, or traboulsi syndrome. Am. J. Hum. Genet..

[2] Shawaf S., Noureddin B., Khouri A., Traboulsi E.I. (1995). A family with a syndrome of ectopia lentis, spontaneous filtering blebs, and craniofacial dysmorphism. Ophthalmic Genet..

[3] Chermakani P., Sundaresan P. (2023). Traboulsi Syndrome: a rare eye disease and its genetic association. TNOA J. Ophthalmic Sci. Res..

[4] Senthil S. (2018). Comment on: bilateral idiopathic spontaneous filtering bleb with ectopia lentis: a case report and review of literature. Indian J. Ophthalmol..

[5] Chandran P., Khairnar A.S., Aboobacker N., Raman G.V. (2018). Response to comment on: bilateral idiopathic spontaneous filtering bleb with ectopia lentis: a case report and review of literature. Indian J. Ophthalmol..

[6] Mansour A.M., Younis M.H., Dakroub R.H. (2013). Anterior segment imaging and treatment of a case with syndrome of ectopia lentis, spontaneous filtering blebs, and craniofacial dysmorphism. Case Rep. Ophthalmol..

[7] Awais T., Ali M., Khan S.A. (2019). Traboulsi syndrome in Pakistan. J. Coll. Physicians Surg. Pak..

[8] Khan A.O. (2024). Potential ASPH-related ectopia lentis. J. Neuroophthalmol..

[9] de Castro D., Pujol-Pocull D., Domínguez F. (2025). Traboulsi syndrome. Eur. Heart J..

[10] Abarca Barriga H.H., Caballero N., Trubnykova M., Castro-Mujica M.D.C., La Serna-Infantes J.E., Vásquez F. (2018). A novel ASPH variant extends the phenotype of Shawaf-Traboulsi syndrome. Am. J. Med. Genet. A..

[11] Shanmugam P., Sagar P., Konana V., Simakurthy S., Ramanjulu R., Sheemar A. (2020). Recurrent unintentional filtering blebs after vitrectomy: a case report. Indian J. Ophthalmol..

[12] Senthil S., Sharma S., Vishwakarma S., Kaur I. (2021). A novel mutation in the aspartate beta-hydroxylase (*ASPH*) gene is associated with a rare form of Traboulsi syndrome. Ophthalmic Genet..

[13] Van Hoorde T., Nerinckx F., Kreps E., Roels D., Huyghe P., Van Heetvelde M. (2021). Expanding the clinical spectrum and management of Traboulsi syndrome: report on two siblings homozygous for a novel pathogenic variant in ASPH. Ophthalmic Genet..

[14] Kulkarni N., Lloyd I.C., Ashworth J., Biswas S., Black G.C.M., Clayton-Smith J. (2019). Traboulsi syndrome due to ASPH mutation: an under-recognised cause of ectopia lentis. Clin. Dysmorphol..

[15] Wang L.-L., Zhang L.-Y., Zhou J.-L. (2024). Traboulsi syndrome: a case report. Asian J. Surg..

[16] Lei C., Guo T., Ding S., Liao L., Peng H., Tan Z. (2021). Whole-exome sequencing identified a novel homozygous ASPH frameshift variant causing Traboulsi syndrome in a Chinese family. Mol. Genet. Genomic Med..

[17] Ibarra-Ramírez M., Campos-Acevedo L.D., Valenzuela-Lopez A., López-Villanueva L.A., Fernandez-de-Luna M., Mohamed-Noriega J. (2024). A new case report of Traboulsi Syndrome: a literature review and insights into genotype–phenotype correlations. Genes.

[18] Beniwal A., Bafna R.K., Roop P., Lata S., Sharma N. (2024). Biological encirclage–traboulsi syndrome. Indian J. Ophthalmol..

[19] Lima F.L., Cronemberger S., Albuquerque A.L.B., Barbosa L.F., Cunha F.R., Veloso A.W. (2023). Traboulsi syndrome without features of Marfan syndrome caused by a novel homozygous *ASPH* variant associated with a heterozygous *FBN1* variant. Ophthalmic Genet..

[20] Musleh M., Bull A., Linton E., Liu J., Waller S., Hardcastle C. (2023). The role of genetic testing in children requiring surgery for ectopia lentis. Genes.

[21] Jones G., Johnson K., Eason J., Hamilton M., Osio D., Kanani F. (2022). Traboulsi syndrome caused by mutations in ASPH: an autosomal recessive disorder with overlapping features of Marfan syndrome. Eur. J. Med. Genet..

[22] Chandran P., Chermakani P., Venkataraman P., Thilagar S.P., Raman G.V., Sundaresan P. (2019). A novel 5 bp homozygous deletion mutation in ASPH gene associates with Traboulsi syndrome. Ophthalmic Genet..

[23] Siggs O.M., Souzeau E., Craig J.E. (2019). Loss of ciliary zonule protein hydroxylation and lens stability as a predicted consequence of biallelic ASPH variation. Ophthalmic Genet..

[24] Haarman A.E.G., Thiadens A.A.H.J., van Tienhoven M., Loudon S.E., de Klein J.E.M.M.A., Brosens E. (2022). Whole exome sequencing of known eye genes reveals genetic causes for high myopia. Hum. Mol. Genet..

[25] Chen Z.-X., Jia W.-N., Sun Y., Jiang Y.-X. (2024). Genotype-phenotype profile of global ASPH-associated ectopia lentis and clinical findings from a Chinese cohort. Gene.

[26] Chee S.-P., Ti S.-E., Chan N.S.-W. (2021). Management of the subluxated crystalline lens: a review. Clin. Exp. Ophthalmol.

[27] Dinchuk J.E., Henderson N.L., Burn T.C., Huber R., Ho S.P., Link J. (2000). Aspartyl β-hydroxylase (Asph) and an evolutionarily conserved isoform of Asph missing the catalytic domain share exons with Junctin. J. Biol. Chem..

[28] Treves S., Feriotto G., Moccagatta L., Gambari R., Zorzato F. (2000). Molecular cloning, expression, functional characterization, chromosomal localization, and gene structure of junctate, a novel integral calcium binding protein of sarco(endo)plasmic reticulum membrane. J. Biol. Chem..

[29] Jones L.R., Zhang L., Sanborn K., Jorgensen A.O., Kelley J. (1995). Purification, primary structure, and immunological characterization of the 26-kDa calsequestrin binding protein (Junctin) from cardiac junctional sarcoplasmic reticulum. J. Biol. Chem..

[30] Pritchard T.J., Kranias E.G. (2009). Junctin and the histidine-rich Ca2+ binding protein: potential roles in heart failure and arrhythmogenesis. J. Physiol..

[31] Dinchuk J.E., Focht R.J., Kelley J.A., Henderson N.L., Zolotarjova N.I., Wynn R. (2002). Absence of post-translational aspartyl β-hydroxylation of epidermal growth factor domains in mice leads to developmental defects and an increased incidence of intestinal neoplasia. J. Biol. Chem..

[32] Stenflo J., Holme E., Lindstedt S., Chandramouli N., Tsai Huang L.H., Tam J.P. (1989). Hydroxylation of aspartic acid in domains homologous to the epidermal growth factor precursor is catalyzed by a 2-oxoglutarate-dependent dioxygenase. Proc. Natl. Acad. Sci. U. S. A..

[33] Gronke R.S., VanDusen W.J., Garsky V.M., Jacobs J.W., Sardana M.K., Stern A.M. (1989). Aspartyl β-hydroxylase: in vitro hydroxylation of a synthetic peptide based on the structure of the first growth factor-like domain of human factor IX. Proc. Natl. Acad. Sci. U. S. A..

[34] Korioth F., Gieffers C., Frey J. (1994). Cloning and characterization of the human gene encoding aspartyl β-hydroxylase. Gene.

[35] McMullen B.A., Fujikawa K., Kisiel W., Sasagawa T., Howald W.N., Kwa E.Y. (1983). Complete amino acid sequence of the light chain of human blood coagulation factor X: evidence for identification of residue 63 as β-hydroxyaspartic acid. Biochemistry.

[36] Fernlund P., Stenflo J. (1983). β-Hydroxyaspartic acid in vitamin K-dependent proteins. J. Biol. Chem..

[37] Takeuchi H., Schneider M., Williamson D.B., Ito A., Takeuchi M., Handford P.A. (2018). Two novel protein O-glucosyltransferases that modify sites distinct from POGLUT1 and affect notch trafficking and signaling. Proc. Natl. Acad. Sci. U. S. A..

[38] Glanville R.W., Qian R.Q., McClure D.W., Maslen C.L. (1994). Calcium binding, hydroxylation, and glycosylation of the precursor epidermal growth factor-like domains of fibrillin-1, the Marfan gene protein. J. Biol. Chem..

[39] Williamson D.B., Sohn C.J., Ito A., Haltiwanger R.S. (2021). POGLUT2 and POGLUT3 O-glucosylate multiple EGF repeats in fibrillin-1, -2, and LTBP1 and promote secretion of fibrillin-1. J. Biol. Chem..

[40] Brewitz L., Onisko B.C., Schofield C.J. (2022). Combined proteomic and biochemical analyses redefine the consensus sequence requirement for epidermal growth factor-like domain hydroxylation. J. Biol. Chem..

[41] Campbell I.D., Bork P. (1993). Epidermal growth factor-like modules. Curr. Opin. Struct. Biol..

[42] Tombling B.J., Wang C.K., Craik D.J. (2020). EGF-like and other disulfide-rich microdomains as therapeutic scaffolds. Angew. Chem. Int. Ed..

[43] Wouters M.A., Rigoutsos I., Chu C.K., Feng L.L., Sparrow D.B., Dunwoodie S.L. (2005). Evolution of distinct EGF domains with specific functions. Protein Sci..

[44] Cooke R.M., Wilkinson A.J., Baron M., Pastore A., Tappin M.J., Campbell I.D. (1987). The solution structure of human epidermal growth factor. Nature.

[45] Weisshuhn P.C., Sheppard D., Taylor P., Whiteman P., Lea S.M., Handford P.A. (2016). Non-linear and flexible regions of the human notch1 extracellular domain revealed by high-resolution structural studies. Structure.

[46] Ogiso H., Ishitani R., Nureki O., Fukai S., Yamanaka M., Kim J.-H. (2002). Crystal structure of the complex of human epidermal growth factor and receptor extracellular domains. Cell.

[47] Brewitz L., Tumber A., Schofield C.J. (2020). Kinetic parameters of human aspartate/asparagine-β-hydroxylase suggest that it has a possible function in oxygen sensing. J. Biol. Chem..

[48] Stenflo J. (1991). Structure-function relationships of epidermal growth factor modules in vitamin K-dependent clotting factors. Blood.

[49] Rana N.A., Haltiwanger R.S. (2011). Fringe benefits: functional and structural impacts of O-glycosylation on the extracellular domain of notch receptors. Curr. Opin. Struct. Biol..

[50] Stanley P. (2007). Regulation of notch signaling by glycosylation. Curr. Opin. Struct. Biol..

[51] Neupane S., Williamson D.B., Roth R.A., Halabi C.M., Haltiwanger R.S., Holdener B.C. (2024). *Poglut2/3* double knockout in mice results in neonatal lethality with reduced levels of fibrillin in lung tissues. J. Biol. Chem..

[52] Pfeffer I., Brewitz L., Krojer T., Jensen S.A., Kochan G.T., Kershaw N.J. (2019). Aspartate/asparagine-β-hydroxylase crystal structures reveal an unexpected epidermal growth factor-like domain substrate disulfide pattern. Nat. Commun..

[53] Schofield C.J., Ratcliffe P.J. (2004). Oxygen sensing by HIF hydroxylases. Nat. Rev. Mol. Cell Biol..

[54] Safran M., Kaelin W.G. (2003). HIF hydroxylation and the mammalian oxygen-sensing pathway. J. Clin. Invest..

[55] Mahon P.C., Hirota K., Semenza G.L. (2001). FIH-1: a novel protein that interacts with HIF-1α and VHL to mediate repression of HIF-1 transcriptional activity. Genes Dev..

[56] Kaelin W.G., Ratcliffe P.J. (2008). Oxygen sensing by metazoans: the central role of the HIF hydroxylase pathway. Mol. Cell.

[57] Lando D., Peet D.J., Whelan D.A., Gorman J.J., Whitelaw M.L. (2002). Asparagine hydroxylation of the HIF transactivation domain: a hypoxic switch. Science.

[58] McNeill L.A., Hewitson K.S., Claridge T.D., Seibel J.F., Horsfall L.E., Schofield C.J. (2002). Hypoxia-inducible factor asparaginyl hydroxylase (FIH-1) catalyses hydroxylation at the β-carbon of asparagine-803. Biochem. J..

[59] Cockman M.E., Lancaster D.E., Stolze I.P., Hewitson K.S., McDonough M.A., Coleman M.L. (2006). Posttranslational hydroxylation of ankyrin repeats in IκB proteins by the hypoxia-inducible factor (HIF) asparaginyl hydroxylase, factor inhibiting HIF (FIH). Proc. Natl. Acad. Sci. U. S. A..

[60] Ferguson J.E., Wu Y., Smith K., Charles P., Powers K., Wang H. (2007). ASB4 is a hydroxylation substrate of FIH and promotes vascular differentiation via an oxygen-dependent mechanism. Mol. Cell. Biol..

[61] Coleman M.L., McDonough M.A., Hewitson K.S., Coles C., Mecinović J., Edelmann M. (2007). Asparaginyl hydroxylation of the notch ankyrin repeat domain by factor inhibiting hypoxia-inducible factor. J. Biol. Chem..

[62] Wu Y., Li Z., McDonough M.A., Schofield C.J., Zhang X. (2021). Inhibition of the oxygen-sensing asparaginyl hydroxylase factor inhibiting hypoxia-inducible factor: a potential hypoxia response modulating strategy. J. Med. Chem..

[63] Liu Y., Sun Y., Huo Q., Song L., Wang X., Shen X. (2025). Genetic landscape and ocular biometric correlations in microspherophakia: insights from a comprehensive patient cohort. Hum. Genomics.

[64] Vivante A., Hwang D.-Y., Kohl S., Chen J., Shril S., Schulz J. (2017). Exome sequencing discerns syndromes in patients from consanguineous families with congenital anomalies of the kidneys and urinary tract. J. Am. Soc. Nephrol..

[65] Brewitz L., Nakashima Y., Schofield C.J. (2021). Synthesis of 2-oxoglutarate derivatives and their evaluation as cosubstrates and inhibitors of human aspartate/asparagine-β-hydroxylase. Chem. Sci..

[66] Brasnett A., Pfeffer I., Brewitz L., Chowdhury R., Nakashima Y., Tumber A. (2021). Human oxygenase variants employing a single protein Fe^II^ ligand are catalytically active. Angew. Chem. Int. Ed..

[67] Greve J.M., Pinkham A.M., Thompson Z., Cowan J.A. (2021). Active site characterization and activity of the human aspartyl (asparaginyl) β-hydroxylase. Metallomics.

[68] Zou Q., Hou Y., Wang H., Wang K., Xing X., Xia Y. (2018). Hydroxylase activity of ASPH promotes hepatocellular carcinoma metastasis through epithelial-to-mesenchymal transition pathway. EBioMedicine.

[69] Brewitz L., Brasnett A., Schnaubelt L.I., Rabe P., Tumber A. (2024). Methods for production and assaying catalysis of isolated recombinant human aspartate/asparagine-β-hydroxylase. Methods Enzymol.

[70] Corner T.P., Salah E., Tumber A., Brewitz L., Schofield C.J. (2025). Biochemical investigations using mass spectrometry to monitor JMJD6-catalysed hydroxylation of multi-lysine containing bromodomain-derived substrates. RSC Chem. Biol..

[71] Rydzik A.M., Leung I.K.H., Kochan G.T., Thalhammer A., Oppermann U., Claridge T.D.W. (2012). Development and application of a fluoride-detection-based fluorescence assay for γ-butyrobetaine hydroxylase. ChemBioChem.

[72] Wehbie R.S., Punekar N.S., Lardy H.A. (1988). Rat liver γ-butyrobetaine hydroxylase catalyzed reaction: influence of potassium, substrates, and substrate analogs on hydroxylation and decarboxylation. Biochemistry.

[73] Rydzik A.M., Leung I.K.H., Kochan G.T., Loik N.D., Henry L., McDonough M.A. (2014). Comparison of the substrate selectivity and biochemical properties of human and bacterial γ-butyrobetaine hydroxylase. Org. Biomol. Chem..

[74] Siess E.A., Brocks D.G., Lattke H.K., Wieland O.H. (1977). Effect of glucagon on metabolite compartmentation in isolated rat liver cells during gluconeogenesis from lactate. Biochem. J..

[75] Thirstrup K., Christensen S., Møller H.A., Ritzén A., Bergström A.-L., Sager T.N. (2011). Endogenous 2-oxoglutarate levels impact potencies of competitive HIF prolyl hydroxylase inhibitors. Pharmacol. Res..

[76] Brewitz L., Nakashima Y., Tumber A., Salah E., Schofield C.J. (2021). Fluorinated derivatives of pyridine-2,4-dicarboxylate are potent inhibitors of human 2-oxoglutarate dependent oxygenases. J. Fluorine Chem..

[77] Brewitz L., Tumber A., Pfeffer I., McDonough M.A., Schofield C.J. (2020). Aspartate/asparagine-β-hydroxylase: a high-throughput mass spectrometric assay for discovery of small molecule inhibitors. Sci. Rep..

[78] McDonough M.A., Kavanagh K.L., Butler D., Searls T., Oppermann U., Schofield C.J. (2005). Structure of human phytanoyl-CoA 2-hydroxylase identifies molecular mechanisms of Refsum disease. J. Biol. Chem..

[79] Majamaa K., Hanauske-Abel H.M., Günzler V., Kivirikko K.I. (1984). The 2-oxoglutarate binding site of prolyl 4-hydroxylase. Eur. J. Biochem..

[80] Cunliffe C.J., Franklin T.J., Hales N.J., Hill G.B. (1992). Novel inhibitors of prolyl 4-hydroxylase. 3. Inhibition by the substrate analog N-oxaloglycine and its derivatives. J. Med. Chem..

[81] Nakashima Y., Brewitz L., Tumber A., Salah E., Schofield C.J. (2021). 2-Oxoglutarate derivatives can selectively enhance or inhibit the activity of human oxygenases. Nat. Commun..

[82] Wilkins A.L., Lu Y., Tan S.-T. (1995). Extractives from New Zealand honeys. 5. Aliphatic dicarboxylic acids in New Zealand rewarewa (*Knightea excelsa*) honey. J. Agric. Food Chem..

[83] Díaz R., Gallart-Ayala H., Sancho J.V., Nuñez O., Zamora T., Martins C.P.B. (2016). Told through the wine: a liquid chromatography–mass spectrometry interplatform comparison reveals the influence of the global approach on the final annotated metabolites in non-targeted metabolomics. J. Chromatogr. A..

[84] Danhauser K., Sauer S.W., Haack T.B., Wieland T., Staufner C., Graf E. (2012). DHTKD1 mutations cause 2-aminoadipic and 2-oxoadipic aciduria. Am. J. Hum. Genet..

[85] Matthews D.E. (2020). Review of lysine metabolism with a focus on humans. J. Nutr..

[86] Drevland R.M., Jia Y., Palmer D.R.J., Graham D.E. (2008). Methanogen homoaconitase catalyzes both hydrolyase reactions in coenzyme B biosynthesis. J. Biol. Chem..

[87] Tober I., Spener F. (1982). Biosynthesis of cyclopentenylglycine from α-ketopimelate in Idesia polycarpa callus cultures. Plant. Cell Rep..

[88] Howell D.M., Harich K., Xu H., White R.H. (1998). α-Keto acid chain elongation reactions involved in the biosynthesis of Coenzyme B (7-mercaptoheptanoyl threonine phosphate) in methanogenic archaea. Biochemistry.

[89] Graham D.E. (2011). 2-oxoacid metabolism in methanogenic CoM and CoB biosynthesis. Methods Enzymol.

[90] Dong S., Zhan Z.Y., Cao H.Y., Wu C., Bian Y.Q., Li J.Y. (2017). Urinary metabolomics analysis identifies key biomarkers of different stages of nonalcoholic fatty liver disease. World J. Gastroenterol..

[91] Kirschning A. (2022). On the evolution of coenzyme biosynthesis. Nat. Prod. Rep..

[92] Tumber A., Salah E., Brewitz L., Corner T.P., Schofield C.J. (2023). Kinetic and inhibition studies on human Jumonji-C (JmjC) domain-containing protein 5. RSC Chem. Biol..

[93] Dhingra S., Zhang Z., Lohans C.T., Brewitz L., Schofield C.J. (2024). Substitution of 2-oxoglutarate alters reaction outcomes of the *Pseudomonas savastanoi* ethylene-forming enzyme. J. Biol. Chem..

[94] Hoegenauer C., Hammer H.F., Mahnert A., Moissl-Eichinger C. (2022). Methanogenic archaea in the human gastrointestinal tract. Nat. Rev. Gastroenterol. Hepatol..

[95] Kahnert A., Kertesz M.A. (2000). Characterization of a sulfur-regulated oxygenative alkylsulfatase from Pseudomonas putida S-313. J. Biol. Chem..

[96] Fukumori F., Hausinger R.P. (1993). Purification and characterization of 2,4-dichlorophenoxyacetate/alpha-ketoglutarate dioxygenase. J. Biol. Chem..

[97] Liu H., Wang C., Lee S., Deng Y., Wither M., Oh S. (2017). Clipping of arginine-methylated histone tails by JMJD5 and JMJD7. Proc. Natl. Acad. Sci. U. S. A..

[98] Liu H., Wang C., Lee S., Ning F., Wang Y., Zhang Q. (2018). Specific recognition of arginine methylated histone tails by JMJD5 and JMJD7. Sci. Rep..

[99] Shen J., Xiang X., Chen L., Wang H., Wu L., Sun Y. (2017). JMJD5 cleaves monomethylated histone H3 N-tail under DNA damaging stress. EMBO Rep..

[100] Zhang N., Fu Z., Linke S., Chicher J., Gorman J.J., Visk D. (2010). The asparaginyl hydroxylase factor inhibiting HIF-1α is an essential regulator of metabolism. Cell Metab..

[101] Scholz C.C., Rodriguez J., Pickel C., Burr S., Fabrizio J.-A., Nolan K.A. (2016). FIH regulates cellular metabolism through hydroxylation of the deubiquitinase OTUB1. PLoS Biol..

[102] Sim J., Cowburn A.S., Palazon A., Madhu B., Tyrakis P.A., Macías D. (2018). The factor inhibiting HIF asparaginyl hydroxylase regulates oxidative metabolism and accelerates metabolic adaptation to hypoxia. Cell Metab..

[103] Kanzaki T., Olofsson A., Morén A., Wernstedt C., Hellman U., Miyazono K. (1990). TGF-β1 binding protein: a component of the large latent complex of TGF-β1 with multiple repeat sequences. Cell.

[104] Bassnett S. (2021). Zinn's zonule. Prog. Retin. Eye. Res..

[105] Robertson I.B., Horiguchi M., Zilberberg L., Dabovic B., Hadjiolova K., Rifkin D.B. (2015). Latent TGF-β-binding proteins. Matrix Biol..

[106] Haji-Seyed-Javadi R., Jelodari-Mamaghani S., Paylakhi S.H., Yazdani S., Nilforushan N., Fan J.-B. (2012). LTBP2 mutations cause Weill–Marchesani and Weill–Marchesani-like syndrome and affect disruptions in the extracellular matrix. Hum. Mutat..

[107] Désir J., Sznajer Y., Depasse F., Roulez F., Schrooyen M., Meire F. (2010). LTBP2 null mutations in an autosomal recessive ocular syndrome with megalocornea, spherophakia, and secondary glaucoma. Eur. J. Hum. Genet..

[108] Ali M., McKibbin M., Booth A., Parry D.A., Jain P., Riazuddin S.A. (2009). Null mutations in LTBP2 cause primary congenital glaucoma. Am. J. Hum. Genet..

[109] Narooie-Nejad M., Paylakhi S.H., Shojaee S., Fazlali Z., Rezaei Kanavi M., Nilforushan N. (2009). Loss of function mutations in the gene encoding latent transforming growth factor beta binding protein 2, LTBP2, cause primary congenital glaucoma. Hum. Mol. Genet..

[110] Azmanov D.N., Dimitrova S., Florez L., Cherninkova S., Draganov D., Morar B. (2011). LTBP2 and CYP1B1 mutations and associated ocular phenotypes in the Roma/Gypsy founder population. Eur. J. Hum. Genet..

[111] Shi Y., Jones W., Beatty W., Tan Q., Mecham R.P., Kumra H. (2021). Latent-transforming growth factor beta-binding protein-2 (LTBP-2) is required for longevity but not for development of zonular fibers. Matrix Biol..

[112] Inoue T., Ohbayashi T., Fujikawa Y., Yoshida H., Akama T.O., Noda K. (2014). Latent TGF-β binding protein-2 is essential for the development of ciliary zonule microfibrils. Hum. Mol. Genet..

[113] De Paepe A., Devereux R.B., Dietz H.C., Hennekam R.C.M., Pyeritz R.E. (1996). Revised diagnostic criteria for the Marfan syndrome. Am. J. Med. Genet..

[114] Nishikawa T., Yamamoto T., Honjo K.-I., Ichioka H., Yamamoto K., Kanamura N. (2013). Marfan's syndrome: clinical manifestations in the oral-craniofacial area, biophysiological roles of fibrillins and elastic extracellular microfibers, and disease control of the fibrillin gene. J. Oral. Maxillofac. Surg. Med. Pathol..

[115] Chen Z.-X., Jia W.-N., Jiang Y.-X. (2022). Genotype-phenotype correlations of Marfan syndrome and related fibrillinopathies: Phenomenon and molecular relevance. Front. Genet..

[116] Milewicz D.M., Braverman A.C., De Backer J., Morris S.A., Boileau C., Maumenee I.H. (2021). Marfan syndrome. Nat. Rev. Dis. Primers..

[117] Sakai L.Y., Keene D.R., Renard M., De Backer J. (2016). FBN1: the disease-causing gene for Marfan syndrome and other genetic disorders. Gene.

[118] Jones W., Rodriguez J., Bassnett S. (2019). Targeted deletion of fibrillin-1 in the mouse eye results in ectopia lentis and other ocular phenotypes associated with Marfan syndrome. Dis. Model. Mech..

[119] Zhang M., Chen Z., Chen T., Sun X., Jiang Y. (2022). Cysteine substitution and calcium-binding mutations in FBN1 cbEGF-like domains are associated with severe ocular involvement in patients with congenital ectopia lentis. Front. Cell Dev. Biol.

[120] Zhou Y., Guo D., Cao Q., Zhang X., Jin G., Zheng D. (2021). Genotype variant screening and phenotypic analysis of *FBN1* in Chinese patients with isolated ectopia lentis. Mol. Med. Rep..

[121] Schrijver I., Liu W., Brenn T., Furthmayr H., Francke U. (1999). Cysteine substitutions in epidermal growth factor–like domains of fibrillin-1: distinct effects on biochemical and clinical phenotypes. Am. J. Hum. Genet..

[122] Ahram D., Sato T.S., Kohilan A., Tayeh M., Chen S., Leal S. (2009). A homozygous mutation in ADAMTSL4 causes autosomal-recessive isolated ectopia lentis. Am. J. Hum. Genet..

[123] Kuang G., Xin B., Sency V., Traboulsi E.I., Cruz V., Wang H. (2024). Ectopia lentis associated with a 20-base deletion in the ADAMTSL4 gene in the old order Amish population. Ophthalmic Genet..

[124] Chandra A., Charteris D. (2014). Molecular pathogenesis and management strategies of ectopia lentis. Eye.

[125] Brewitz L., Tumber A., Thalhammer A., Salah E., Christensen K.E., Schofield C.J. (2020). Synthesis of novel pyridine-carboxylates as small-molecule inhibitors of human aspartate/asparagine-β-hydroxylase. ChemMedChem.

[126] Aihara A., Huang C.-K., Olsen M.J., Lin Q., Chung W., Tang Q. (2014). A cell-surface β-hydroxylase is a biomarker and therapeutic target for hepatocellular carcinoma. Hepatology.

[127] Dong X., Lin Q., Aihara A., Li Y., Huang C.-K., Chung W. (2014). Aspartate β-hydroxylase expression promotes a malignant pancreatic cellular phenotype. Oncotarget.

[128] Brewitz L., Tumber A., Zhang X., Schofield C.J. (2020). Small-molecule active pharmaceutical ingredients of approved cancer therapeutics inhibit human aspartate/asparagine-β-hydroxylase. Bioorg. Med. Chem..

[129] Brewitz L., Nakashima Y., Piasecka S.K., Salah E., Fletcher S.C., Tumber A. (2023). 5-Substituted pyridine-2,4-dicarboxylate derivatives have potential for selective inhibition of human Jumonji-C Domain-containing protein 5. J. Med. Chem..

[130] Lavaissiere L., Jia S., Nishiyama M., de la Monte S., Stern A.M., Wands J.R. (1996). Overexpression of human aspartyl(asparaginyl)β-hydroxylase in hepatocellular carcinoma and cholangiocarcinoma. J. Clin. Invest.

[131] Ince N., de la Monte S.M., Wands J.R. (2000). Overexpression of human aspartyl (asparaginyl) β-hydroxylase is associated with malignant transformation. Cancer Res..

[132] Kanwal M., Smahel M., Olsen M., Smahelova J., Tachezy R. (2020). Aspartate β-hydroxylase as a target for cancer therapy. J. Exp. Clin. Cancer Res..

[133] Zheng W., Wang X., Hu J., Bai B., Zhu H. (2020). Diverse molecular functions of aspartate β-hydroxylase in cancer. Oncol. Rep..

[134] Kanwal M., Polakova I., Olsen M., Kasi M.K., Tachezy R., Smahel M. (2024). Heterogeneous response of tumor cell lines to inhibition of aspartate β-hydroxylase. J. Cancer.

[135] Sun X., Hart J., Taliano R., Molino J., Schwab J.H., Nota S. (2025). ASPH is a metastatic factor and therapeutic target in chondrosarcoma. Cancers.

[136] Yüksel D., Orban de Xivry J.-J., Lefèvre P. (2010). Review of the major findings about Duane retraction syndrome (DRS) leading to an updated form of classification. Vis. Res..

[137] Kekunnaya R., Negalur M. (2017). Duane retraction syndrome: causes, effects and management strategies. Clin. Ophthalmol..

[138] Lehman A.M., Friedman J.M., Chai D., Zahir F.R., Marra M.A., Prisman L. (2009). A characteristic syndrome associated with microduplication of 8q12, inclusive of CHD7. Eur. J. Med. Genet..

[139] Amouroux C., Vincent M., Blanchet P., Puechberty J., Schneider A., Chaze A.M. (2012). Duplication 8q12: confirmation of a novel recognizable phenotype with duane retraction syndrome and developmental delay. Eur. J. Hum. Genet..

[140] Baroncini A., Bertuzzo S., Quarantini R., Ricciardelli P., Giorda R., Bonaglia M.C. (2013). 8q12 microduplication including CHD7: clinical report on a new patient with Duane retraction syndrome type 3. Mol. Cytogenet..

[141] Luo H., Xie L., Wang S.-Z., Chen J.-L., Huang C., Wang J. (2012). Duplication of 8q12 encompassing CHD7 is associated with a distinct phenotype but without duane anomaly. Eur. J. Med. Genet..

[142] Monfort S., Roselló M., Orellana C., Oltra S., Blesa D., Kok K. (2008). Detection of known and novel genomic rearrangements by array based comparative genomic hybridisation: deletion of ZNF533 and duplication of CHARGE syndrome genes. J. Med. Genet..

[143] Baris H.N., Chan W.-M., Andrews C., Behar D.M., Donovan D.J., Morton C.C. (2013). Complex cytogenetic rearrangements at the DURS1 locus in syndromic Duane retraction syndrome. Clin. Case Rep..

[144] Lederer D., Grisart B., Digilio M.C., Benoit V., Crespin M., Ghariani S.C. (2012). Deletion of KDM6A, a histone demethylase interacting with MLL2, in three patients with Kabuki syndrome. Am. J. Hum. Genet..

[145] Bögershausen N., Gatinois V., Riehmer V., Kayserili H., Becker J., Thoenes M. (2016). Mutation update for Kabuki syndrome genes KMT2D and KDM6A and further delineation of X-linked Kabuki syndrome subtype 2. Hum. Mutat..

[146] Adam M.P., Banka S., Bjornsson H.T., Bodamer O., Chudley A.E., Harris J. (2019). Kabuki syndrome: international consensus diagnostic criteria. J. Med. Genet..

[147] Lan F., Bayliss P.E., Rinn J.L., Whetstine J.R., Wang J.K., Chen S. (2007). A histone H3 lysine 27 demethylase regulates animal posterior development. Nature.

[148] Lee M.G., Villa R., Trojer P., Norman J., Yan K.-P., Reinberg D. (2007). Demethylation of H3K27 regulates polycomb recruitment and H2A ubiquitination. Science.

[149] Hong S., Cho Y.-W., Yu L.-R., Yu H., Veenstra T.D., Ge K. (2007). Identification of JmjC domain-containing UTX and JMJD3 as histone H3 lysine 27 demethylases. Proc. Natl. Acad. Sci. U. S. A..

[150] Tie F., Banerjee R., Conrad P.A., Scacheri P.C., Harte P.J. (2012). Histone demethylase UTX and chromatin remodeler BRM bind directly to CBP and modulate acetylation of histone H3 lysine 27. Mol. Cell. Biol.

[151] Banka S., Lederer D., Benoit V., Jenkins E., Howard E., Bunstone S. (2015). Novel KDM6A (UTX) mutations and a clinical and molecular review of the X-linked Kabuki syndrome (KS2). Clin. Genet..

[152] Faundes V., Goh S., Akilapa R., Bezuidenhout H., Bjornsson H.T., Bradley L. (2021). Clinical delineation, sex differences, and genotype–phenotype correlation in pathogenic KDM6A variants causing X-linked Kabuki syndrome type 2. Genet. Med..

[153] Kato H., Asamitsu K., Sun W., Kitajima S., Yoshizawa-Sugata N., Okamoto T. (2020). Cancer-derived UTX TPR mutations G137V and D336G impair interaction with MLL3/4 complexes and affect UTX subcellular localization. Oncogene.

[154] Wang L., Shilatifard A. (2019). UTX mutations in human cancer. Cancer Cell.

[155] Zeytuni N., Zarivach R. (2012). Structural and functional discussion of the tetra-trico-peptide repeat, a protein interaction module. Structure.

[156] Seidel S., Garvalov B.K., Wirta V., von Stechow L., Schänzer A., Meletis K. (2010). A hypoxic niche regulates glioblastoma stem cells through hypoxia inducible factor 2α. Brain.

[157] Sun S., Deng M., Wen J., Chen X., Xu J., Liu Y. (2023). Aspartate beta-hydroxylase domain containing 1 as a prognostic marker associated with immune infiltration in skin cutaneous melanoma. BMC Cancer.

[158] Wierzbicki A.S., Lloyd M.D., Schofield C.J., Feher M.D., Gibberd F.B. (2002). Refsum's disease: a peroxisomal disorder affecting phytanic acid α-oxidation. J. Neurochem..

[159] Schofield C.J., McDonough M.A. (2007). Structural and mechanistic studies on the peroxisomal oxygenase phytanoyl-CoA 2-hydroxylase (PhyH). Biochem. Soc. Trans..

[160] Jansen G.A., Hogenhout E.M., Ferdinandusse S., Waterham H.R., Ofman R., Jakobs C. (2000). Human phytanoyl-CoA hydroxylase: resolution of the gene structure and the molecular basis of Refsum’s disease. Hum. Mol. Genet..

[161] Mukherji M., Chien W., Kershaw N.J., Clifton I.J., Schofield C.J., Wierzbicki A.S. (2001). Structure–function analysis of phytanoyl-CoA 2-hydroxylase mutations causing Refsum’s disease. Hum. Mol. Genet..

[162] Hanauske-Abel H.M., Günzler V. (1982). A stereochemical concept for the catalytic mechanism of prolylhydroxylase: applicability to classification and design of inhibitors. J. Theor. Biol..

[163] Martinez S., Hausinger R.P. (2015). Catalytic mechanisms of Fe(II)- and 2-oxoglutarate-dependent oxygenases. J. Biol. Chem..

[164] Kal S., Que L. (2017). Dioxygen activation by nonheme iron enzymes with the 2-His-1-carboxylate facial triad that generate high-valent oxoiron oxidants. J. Biol. Inorg. Chem..

[165] Gao S.-S., Naowarojna N., Cheng R., Liu X., Liu P. (2018). Recent examples of α-ketoglutarate-dependent mononuclear non-haem iron enzymes in natural product biosyntheses. Nat. Prod. Rep..

[166] Hausinger R.P., Schofield C.J. (2015). 2-Oxoglutarate-dependent Oxygenases.

[167] Jansen G.A., Waterham H.R., Wanders R.J.A. (2004). Molecular basis of Refsum disease: sequence variations in phytanoyl-CoA hydroxylase (PHYH) and the PTS2 receptor (PEX7). Hum. Mutat..

[168] Hewitson K.S., Holmes S.L., Ehrismann D., Hardy A.P., Chowdhury R., Schofield C.J. (2008). Evidence that two enzyme-derived histidine ligands are sufficient for iron binding and catalysis by factor inhibiting HIF (FIH). J. Biol. Chem..

[169] Iyer S.R., Chaplin V.D., Knapp M.J., Solomon E.I. (2018). O_2_ activation by nonheme Fe^II^ α-ketoglutarate-dependent enzyme variants: elucidating the role of the facial triad carboxylate in FIH. J. Am. Chem. Soc..

[170] Rots D., Jakub T.E., Keung C., Jackson A., Banka S., Pfundt R. (2023). The clinical and molecular spectrum of the KDM6B-related neurodevelopmental disorder. Am. J. Hum. Genet..

[171] Nagahama K., Ogawa T., Fujii T., Tazaki M., Tanase S., Morino Y. (1991). Purification and properties of an ethylene-forming enzyme from Pseudomonas syringae pv. phaseolicola PK2. J. Gen. Microbiol..

[172] Fukuda H., Ogawa T., Ishihara K., Fujii T., Nagahama K., Omata T. (1992). Molecular cloning in Escherichia coli, expression, and nucleotide sequence of the gene for the ethylene-forming enzyme of Pseudomonas syringae pv. phaseolicola PK2. Biochem. Biophys. Res. Commun..

[173] Fukuda H., Ogawa T., Tazaki M., Nagahama K., Fujii T., Tanase S. (1992). Two reactions are simultaneously catalyzed by a single enzyme: the arginine-dependent simultaneous formation of two products, ethylene and succinate, from 2-oxoglutarate by an enzyme from Pseudomonas syringae. Biochem. Biophys. Res. Commun..

[174] Nagahama K., Ogawa T., Fujii T., Tazaki M., Goto M., Fukuda H. (1991). L-Arginine is essential for the formation *in vitro* of ethylene by an extract of *Pseudomonas syringae*. J. Gen. Microbiol..

[175] Martinez S., Fellner M., Herr C.Q., Ritchie A., Hu J., Hausinger R.P. (2017). Structures and mechanisms of the non-heme Fe(II)- and 2-oxoglutarate-dependent ethylene-forming enzyme: substrate binding creates a twist. J. Am. Chem. Soc..

[176] Zhang Z., Smart T.J., Choi H., Hardy F., Lohans C.T., Abboud M.I. (2017). Structural and stereoelectronic insights into oxygenase-catalyzed formation of ethylene from 2-oxoglutarate. Proc. Natl. Acad. Sci. U. S. A..

[177] Copeland R.A., Zhou S., Schaperdoth I., Shoda T.K.C., Bollinger J.M., Krebs C. (2021). Hybrid radical-polar pathway for excision of ethylene from 2-oxoglutarate by an iron oxygenase. Science.

[178] Burke E.J., Copeland R.A., Dixit Y., Krebs C., Bollinger J.M. (2024). Steric perturbation of the Grob-like final step of ethylene-forming enzyme enables 3-hydroxypropionate and propylene production. J. Am. Chem. Soc..

[179] Mukherji M., Kershaw N.J., MacKinnon C.H., Clifton I.J., Wierzbicki A.S., Schofield C.J. (2001). ‘Chemical co-substrate rescue’ of phytanoyl-CoA 2-hydroxylase mutants causing Refsum’s disease. Chem. Commun..

[180] Breski M., Dey D., Obringer S., Sudhamalla B., Islam K. (2016). Engineering biological C–H functionalization leads to allele-specific regulation of histone demethylases. J. Am. Chem. Soc..

[181] Moran G.R. (2014). 4-Hydroxyphenylpyruvate dioxygenase and hydroxymandelate synthase: exemplars of the α-keto acid dependent oxygenases. Arch. Biochem. Biophys..

[182] Li J.J., Kim J.J., Nausheen F. (2023). Phytanic acid intake and lifestyle modifications on quality of life in individuals with adult Refsum disease: a retrospective survey analysis. Nutrients.

[183] Morton D.H., Strauss K.A., Robinson D.L., Puffenberger E.G., Kelley R.I. (2002). Diagnosis and treatment of maple syrup disease: a study of 36 patients. Pediatrics.

[184] Winter G. (2010). xia2: an expert system for macromolecular crystallography data reduction. J. Appl. Cryst..

[185] McCoy A.J., Grosse-Kunstleve R.W., Adams P.D., Winn M.D., Storoni L.C., Read R.J. (2007). Phaser crystallographic software. J. Appl. Cryst..

[186] Adams P.D., Afonine P.V., Bunkóczi G., Chen V.B., Davis I.W., Echols N. (2010). PHENIX: a comprehensive Python-based system for macromolecular structure solution. Acta Cryst. D.

[187] Emsley P., Lohkamp B., Scott W.G., Cowtan K. (2010). Features and development of coot. Acta Cryst. D.

[188] Geoghegan K.F., Dixon H.B.F., Rosner P.J., Hoth L.R., Lanzetti A.J., Borzilleri K.A. (1999). Spontaneous α-N-6-phosphogluconoylation of a “His Tag” in Escherichia coli: the cause of extra mass of 258 or 178 Da in fusion proteins. Anal. Chem..

[189] Yan Z., Caldwell G.W., McDonell P.A. (1999). Identification of a gluconic acid derivative attached to the N-terminus of histidine-tagged proteins expressed in bacteria. Biochem. Biophys. Res. Commun..

