# Supplementary material for: Structural and functional consequences of aspartate/asparagine-β-hydroxylase variants causing Traboulsi syndrome
Source: J Biol Chem. 2025 Dec 5;302(2):111008. doi: 10.1016/j.jbc.2025.111008 (PMC12816917; doi:10.1016/j.jbc.2025.111008)
Supplement: Supplementary Material 1 [file mmc1.pdf]

Supporting Information

Structural and functional consequences of aspartate/asparagine-β-hydroxylase variants causing Traboulsi Syndrome

Cynthia X. Hou[1,#], Amelia Brasnett[1,#], Patrick Rabe[1], Christopher J. Schofield[1,*], and Lennart Brewitz[1,*]

[1]*Chemistry Research Laboratory and the Ineos Oxford Institute for Antimicrobial Research, University of Oxford, 12 Mansfield Road, OX1 3TA, Oxford, United Kingdom.*

# denotes equal contribution

*E-mail: christopher.schofield@chem.ox.ac.uk or lennart.brewitz@chem.ox.ac.uk

Table of contents

1. Supporting figures S2-S41
2. Supporting tables S42-S52
3. References S53

1. Supporting figures

Supporting Figure S1. The canonical and the non-canonical EGFD disulfide connectivity patterns and the structure of the synthetic AspH substrate used in this work. (**a**) The canonical EGFD Cys 1–3, 2–4, 5–6 disulfide pattern. EGFDs with this disulfide pattern are not AspH substrates *in vitro*, even if they bear the AspH consensus sequence (1-3). (**b**) The non-canonical EGFD Cys 1–2, 3–4, 5–6 disulfide pattern. EGFDs with this disulfide pattern, that also bear the AspH consensus sequence, are AspH substrates *in vitro* (1-3). (**c**) The hFX-EGFD1$_{86\text{-}124}$-4S peptide (1), the sequence of which is based on that of EGFD1 from the characterized AspH substrate human coagulation Factor X (hFX) (4, 5). The hFX-EGFD1$_{86\text{-}124}$-4S peptide was used in this work as an AspH substrate for turnover studies and crystallographic analyses; it was synthesized as a C-terminal amide by solid phase peptide synthesis and purified by GL Biochem (Shanghai) Ltd.
Red: residues at the positions of EGFD cysteines 1-6; yellow: cystine sulfurs; orange: the AspH hydroxylation site (1-3); green: EGFD residues binding to the tetratricopeptide repeat (TPR) domain of AspH, an interaction important for productive catalysis (1).

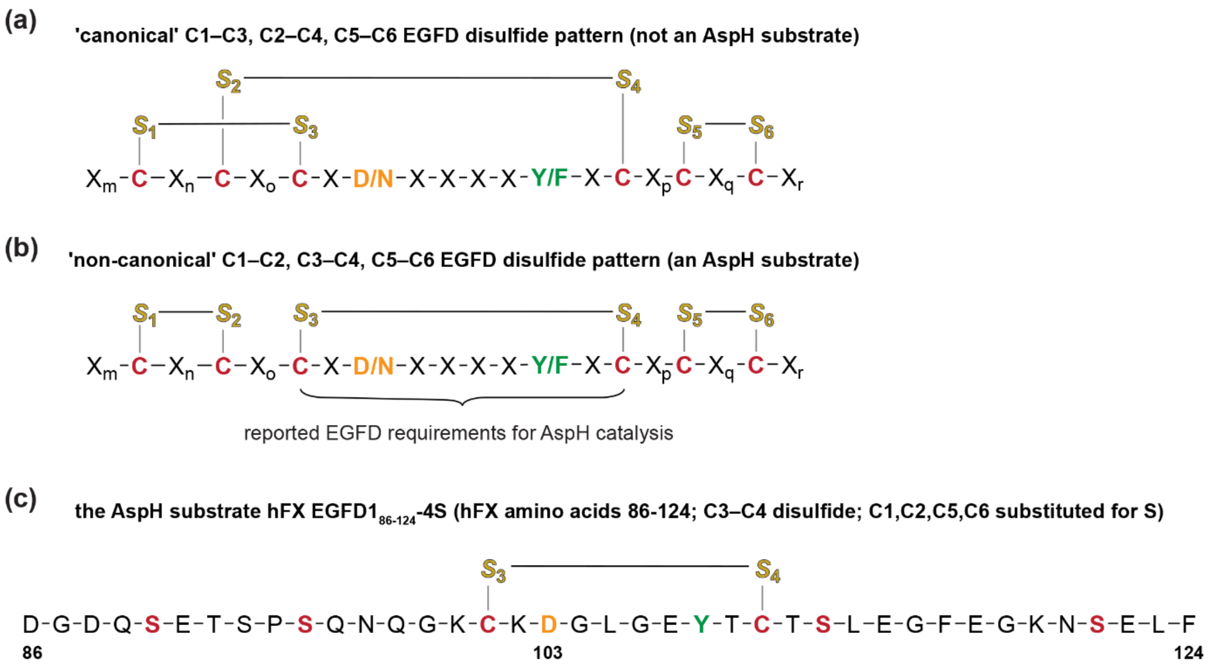

Supporting Figure S2. Traboulsi Syndrome-associated AspH variants were obtained in highly purified form. Sodium dodecyl sulfate polyacrylamide gel electrophoresis (SDS-PAGE) with Coomassie staining and mass spectrometric (MS) analyses of purified human N-terminally His$_6$-tagged: (**a**) wt His$_6$-AspH$_{315-758}$, (**b**) G434V His$_6$-AspH$_{315-758}$, (**c**) R688Q His$_6$-AspH$_{315-758}$, and (**d**) R735W His$_6$-AspH$_{315-758}$ manifest >95% purity post size-exclusion chromatography. Note that additional species (+178 Da) correspond to His$_6$-AspH$_{315-758}$ variants containing a gluconoylated His$_6$-tag, a common covalent modification of recombinant *N*-terminal His$_6$-tagged proteins produced in *E. coli* (6, 7), which does not impact substantially (if at all) on AspH-catalysis.

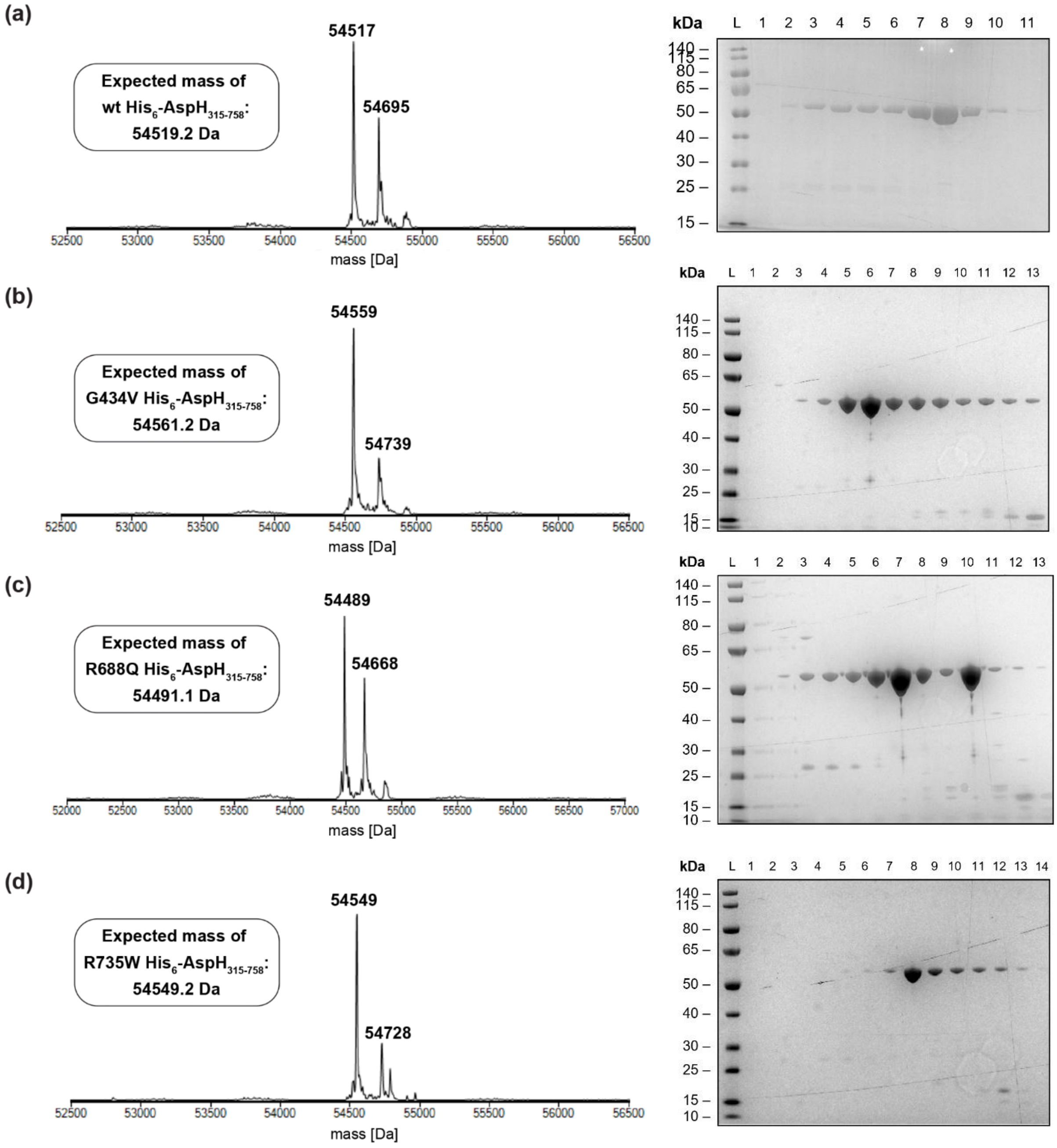

Supporting Figure S3. Presence of an N-terminal His$_6$-tag does not substantially affect catalysis of the Traboulsi Syndrome-associated R735Q AspH variant. (**a** and **b**) SDS-PAGE with Coomassie staining and MS analyses of: (**a**) purified human N-terminally His$_6$-tagged R735Q His$_6$-AspH$_{315\text{-}758}$ and (**b**) MS analyses of purified human R735Q AspH$_{315\text{-}758}$ manifest >95% purity post size-exclusion chromatography. Note that additional species (+178 Da) observed with R735Q His$_6$-AspH$_{315\text{-}758}$ corresponds to the AspH variant containing a gluconoylated His$_6$-tag, a common covalent modification of recombinant *N*-terminal His$_6$-tagged proteins produced in *E. coli* (6, 7), which does not impact substantially (if at all) on AspH-catalysis. (**c**) SPE-MS assays with R735Q His$_6$-AspH$_{315\text{-}758}$ (green boxes) and R735Q AspH$_{315\text{-}758}$ (red circles) demonstrate that removal of the N-terminal His$_6$-tag using thrombin (8) did not substantially alter the activity of R735Q AspH, and, by implication, likely of the other tested AspH variants. Conditions: R735Q His$_6$-AspH$_{315\text{-}758}$ (0.2 µM) or R735Q AspH$_{315\text{-}758}$ (0.2 µM), hFX-EGFD1$_{86\text{-}124}$-4S (4.0 µM), L-ascorbic acid (400 µM), $(NH_4)_2Fe(SO_4)_2\cdot 6H_2O$ (100 µM), and 2OG (2000 µM) in buffer (25 mM MES, pH 6.0, 20 °C).

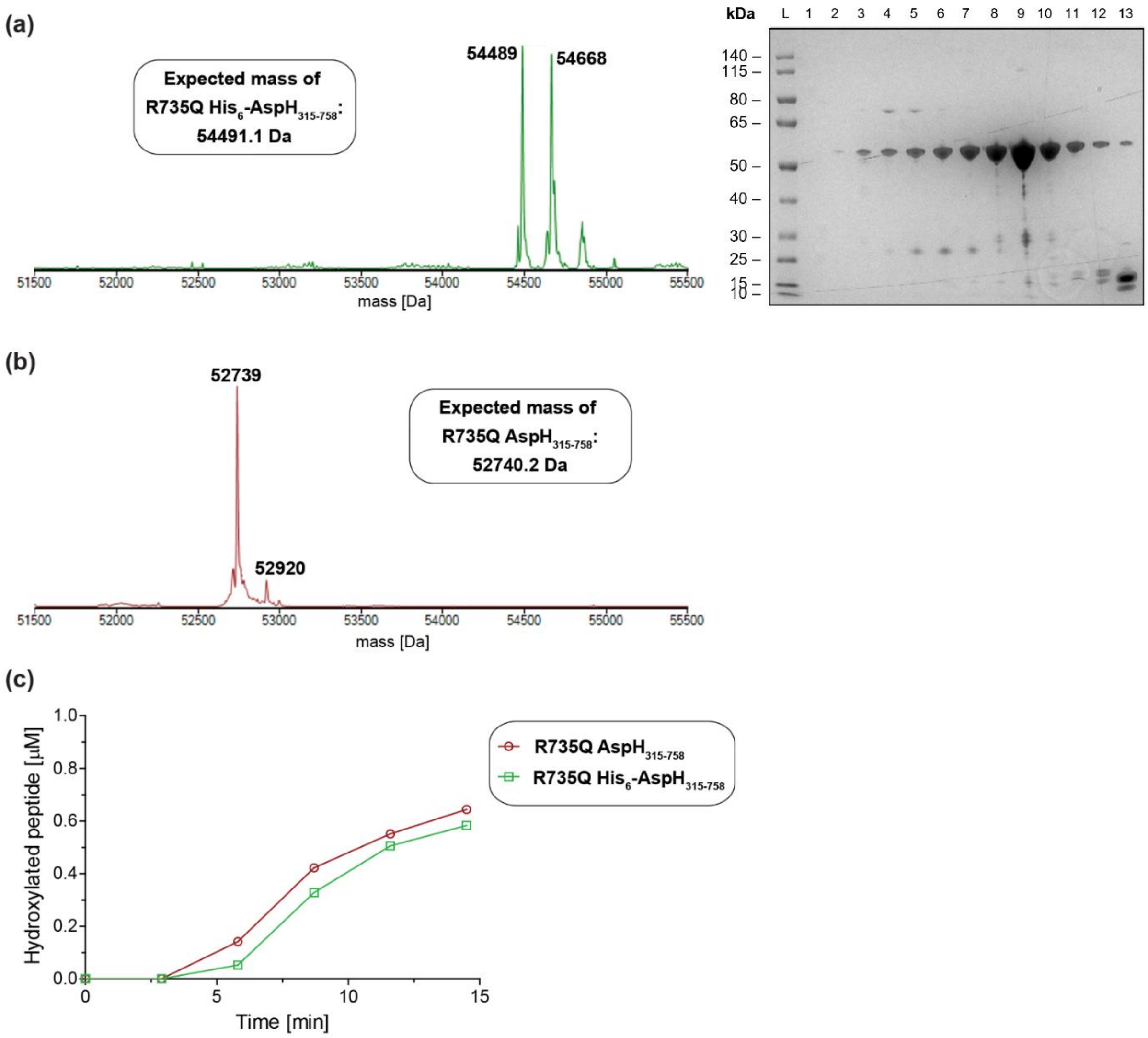

Supporting Figure S4. ^{1}H NMR turnover studies. Endpoint ^{1}H NMR assays were performed using a Bruker AVIII 700 MHz NMR machine equipped with a 5 mm ^{1}H(^{13}C/^{15}N) inverse cryoprobe to investigate 2OG depletion and succinate formation of samples containing an AspH variant (2.5 μM), $(NH_4)_2Fe(SO_4)_2{\cdot}6H_2O$ (50 μM), and equimolar amounts of 2OG and hFX-EGFD1$_{86\text{-}124}$-4S (150 μM) in buffer (25 mM phosphate buffer, 10% (v/v) D_2O, pH 6.0 for R735Q His$_6$-AspH; and: 25 mM Tris-d_{11}, 10% (v/v) D_2O, pH 7.5 for both wt AspH and G434V His$_6$-AspH), which were incubated in air for 16-18 h post AspH addition. Following ^{1}H NMR analysis (gray; right y-axis), samples were analyzed by SPE-MS to quantify substrate hydroxylation (purple; left y-axis).

Note that although R688Q AspH catalysed hydroxylation of hFX-EGFD1$_{86\text{-}124}$-4S under SPE-MS assay conditions, it was apparently inactive under the ^{1}H NMR assay conditions, an observation which, at least in part, may suggest that the buffer affects catalysis (25 mM phosphate buffer, 10% (v/v) D_2O, pH 6.0; or: 25 mM Tris-d_{11}, 10% (v/v) D_2O, pH 7.5 for ^{1}H NMR assays; 25 mM MES, pH 6.0 for SPE-MS assays) and/or that high enzyme/substrate/product concentrations may inhibit R688Q AspH.

The results for: (**a**) wt AspH, (**b**) G434V His$_6$-AspH, and (**c**) R735Q His$_6$-AspH indicate that 2OG turnover is tightly coupled to hFX-EGFD1$_{86\text{-}124}$-4S hydroxylation. In accord with the SPE-MS assay results (Figure 2), catalysis of wt AspH and G434V AspH was substantially more efficient than that of R735Q AspH. Note that the 2OG and succinate signals used for analysis are imperfectly separated (representative spectrum in: **d**), likely perturbing data accuracy at low turnover levels, as observed in the case of R735Q AspH (**c**).

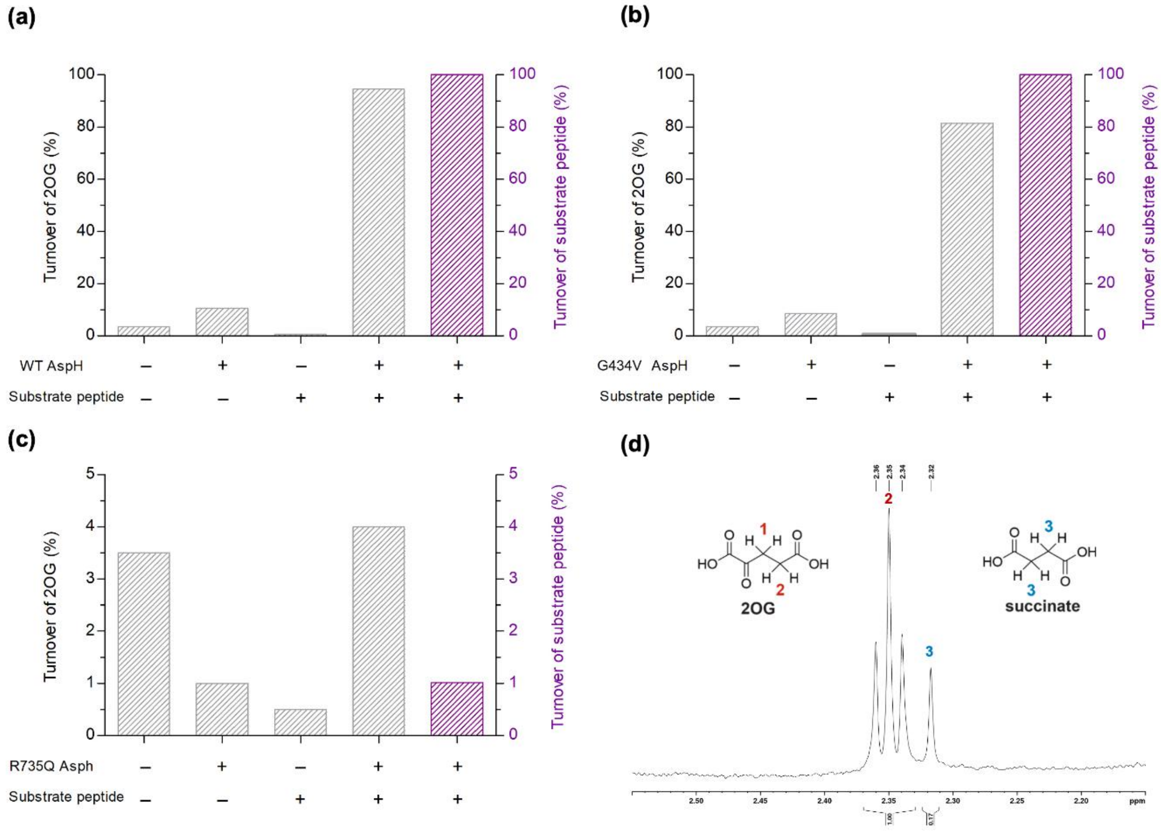

Supporting Figure S5. Reaction rates for determining kinetic parameters of G434V AspH (continues on the following page). Maximum velocities (v_{max}^{app}) and apparent Michaelis constants (K_m^{app}) of isolated recombinant G434V AspH were determined for 2OG and Fe(II), monitoring the G434V AspH-catalyzed hydroxylation of hFX-EGFD1$_{86\text{-}124}$-4S (Supporting Figure S1c) by SPE-MS as described (2, 9). Conditions: G434V His$_6$-AspH$_{315\text{-}758}$ (0.1 µM), hFX-EGFD1$_{86\text{-}124}$-4S (4.0 µM), and the shown concentrations of 2OG, L-ascorbic acid (LAA), and $(NH_4)_2Fe(SO_4)_2 \cdot 6H_2O$ in buffer (25 mM HEPES, pH 7.5, 50 mM NaCl, 20 °C). Measurement times were normalized to the first sample injection analyzed after the addition of G434V AspH to the Substrate Mixture (t = 0 s), by which time low levels of substrate oxidation were manifest. Note, the differences in y-intercept values for different conditions reflect differences in the extent of reaction during the delay between initiation of reaction by AspH addition and the first MS analysis. Data are means of independent triplicates (n = 3; mean ± SD).

(**a**) Time course of the G434V AspH-catalyzed oxidation of hFX-EGFD1$_{86\text{-}124}$-4S (Supporting Figure S1c) for the shown concentrations of LAA using 2OG (20 µM) and $(NH_4)_2Fe(SO_4)_2 \cdot 6H_2O$ (20 µM); (**b**) oxidation rates used to determine kinetic parameters of G434V AspH for LAA.

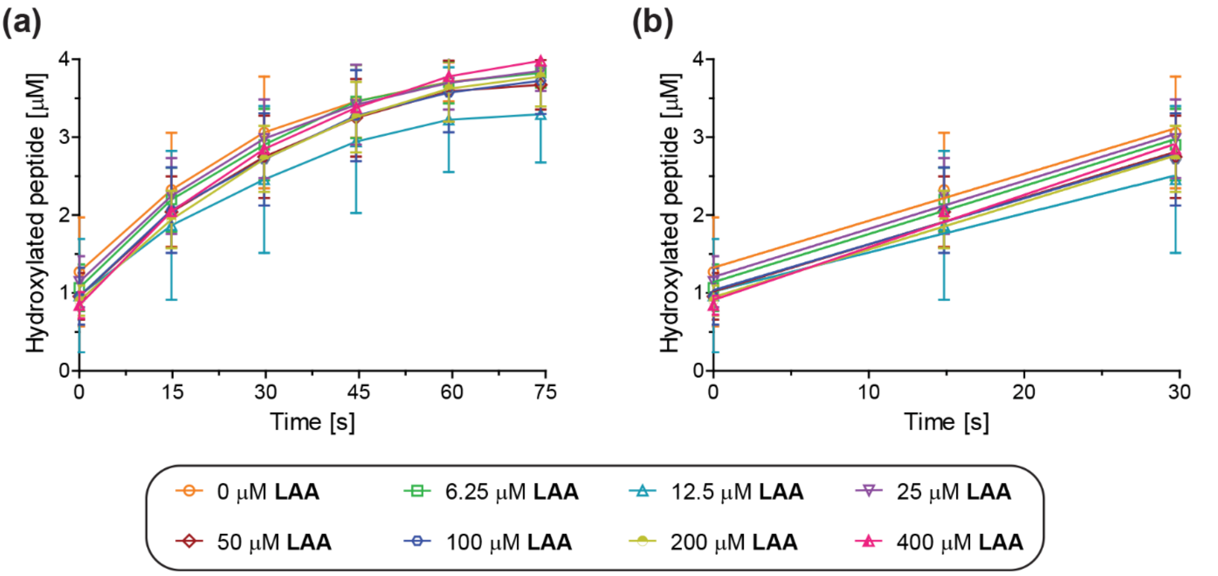

(**c**) Time course of the G434V AspH-catalyzed oxidation of hFX-EGFD1$_{86\text{-}124}$-4S (Supporting Figure S1c) for the shown concentrations of 2OG using LAA (100 µM) and $(NH_4)_2Fe(SO_4)_2 \cdot 6H_2O$ (20 µM); (**d**) oxidation rates used to determine kinetic parameters of G434V AspH for 2OG.

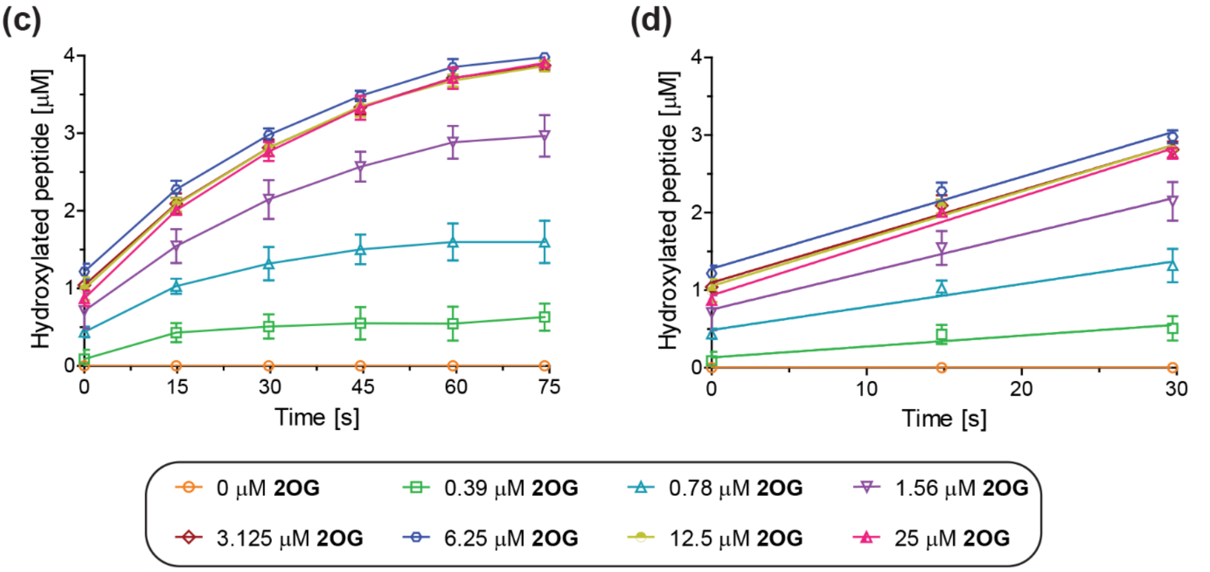

(**e**) Time course of the G434V AspH-catalyzed oxidation of hFX-EGFD1$_{86\text{-}124}$-4S (Supporting Figure S1c) for the shown concentrations of $(NH_4)_2Fe(SO_4)_2 \cdot 6H_2O$ using LAA (100 µM) and 2OG (20 µM); (**f**) oxidation rates used to determine kinetic parameters of G434V AspH for Fe(II).

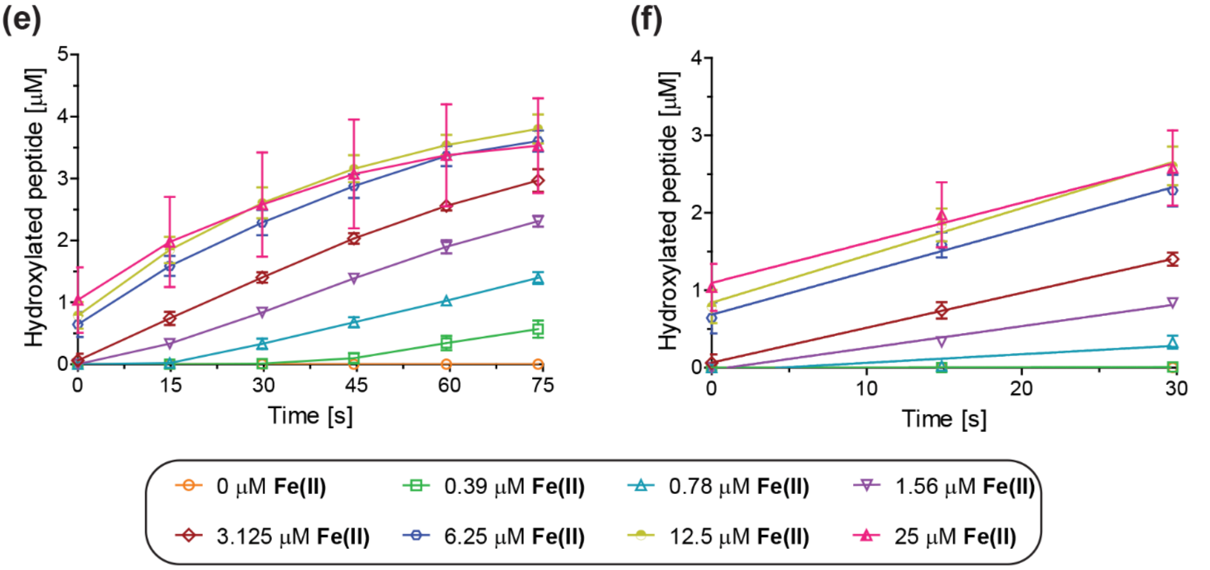

Supporting Figure S6. Reaction rates for determining kinetic parameters of R688Q AspH (continues on the following page). Maximum velocities (v_{max}^{app}) and apparent Michaelis constants (K_m^{app}) of isolated recombinant R688Q AspH were determined for 2OG and Fe(II), monitoring the R688Q AspH-catalyzed hydroxylation of hFX-EGFD1$_{86\text{-}124}$-4S (Supporting Figure S1c) by SPE-MS as described (2, 9). Conditions: R688Q His$_6$-AspH$_{315\text{-}758}$ (0.2 µM), hFX-EGFD1$_{86\text{-}124}$-4S (4.0 µM), and the shown concentrations of 2OG, L-ascorbic acid (LAA), and $(NH_4)_2Fe(SO_4)_2\cdot 6H_2O$ in buffer (25 mM MES, pH 6.0, 20 °C). Measurement times were normalized to the first sample injection analyzed after the addition of R688Q AspH to the Substrate Mixture (t = 0 s), by which time low levels of substrate oxidation were manifest. Note, the differences in y-intercept values for different conditions reflect differences in the extent of reaction during the delay between initiation of reaction by AspH addition and the first MS analysis. Data are means of independent triplicates (n = 3; mean ± SD).

(**a**) Time course of the R688Q AspH-catalyzed oxidation of hFX-EGFD1$_{86\text{-}124}$-4S (Supporting Figure S1c) for the shown concentrations of LAA using 2OG (30 µM) and $(NH_4)_2Fe(SO_4)_2\cdot 6H_2O$ (100 µM); (**b**) oxidation rates used to determine kinetic parameters of R688Q AspH for LAA.

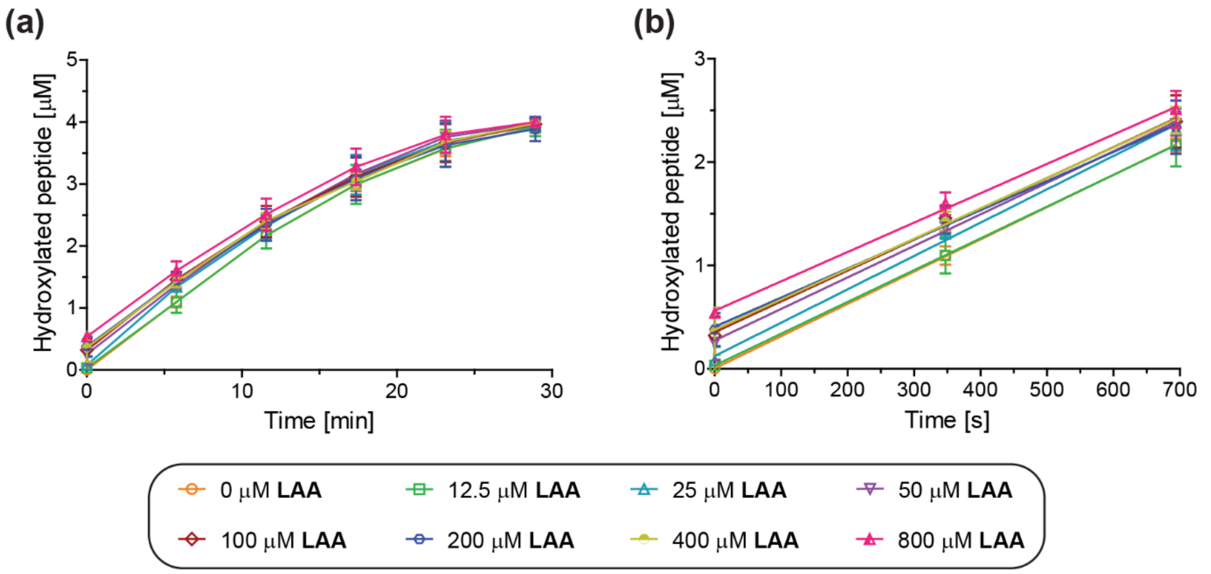

(**c**) Time course of the R688Q AspH-catalyzed oxidation of hFX-EGFD1$_{86\text{-}124}$-4S (Supporting Figure S1c) for the shown concentrations of 2OG using LAA (400 μM) and $(NH_4)_2Fe(SO_4)_2 \cdot 6H_2O$ (100 μM); (**d**) oxidation rates used to determine kinetic parameters of R688Q AspH for 2OG.

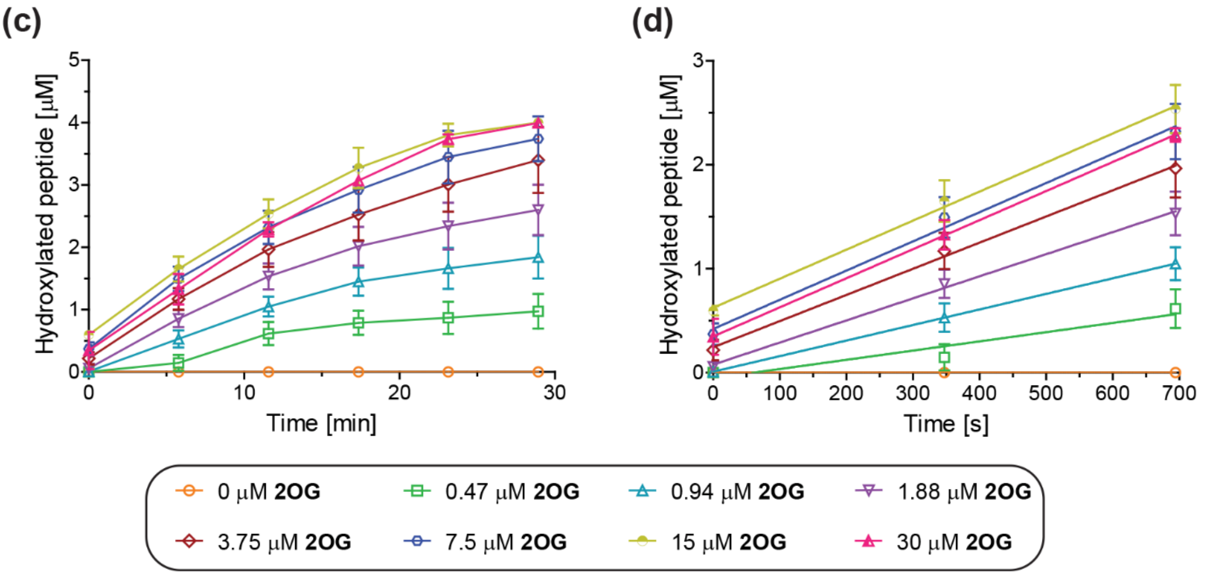

(**e**) Time course of the R688Q AspH-catalyzed oxidation of hFX-EGFD1$_{86\text{-}124}$-4S (Supporting Figure S1c) for the shown concentrations of $(NH_4)_2Fe(SO_4)_2 \cdot 6H_2O$ using LAA (400 μM) and 2OG (30 μM); (**f**) oxidation rates used to determine kinetic parameters of R688Q AspH for Fe(II).

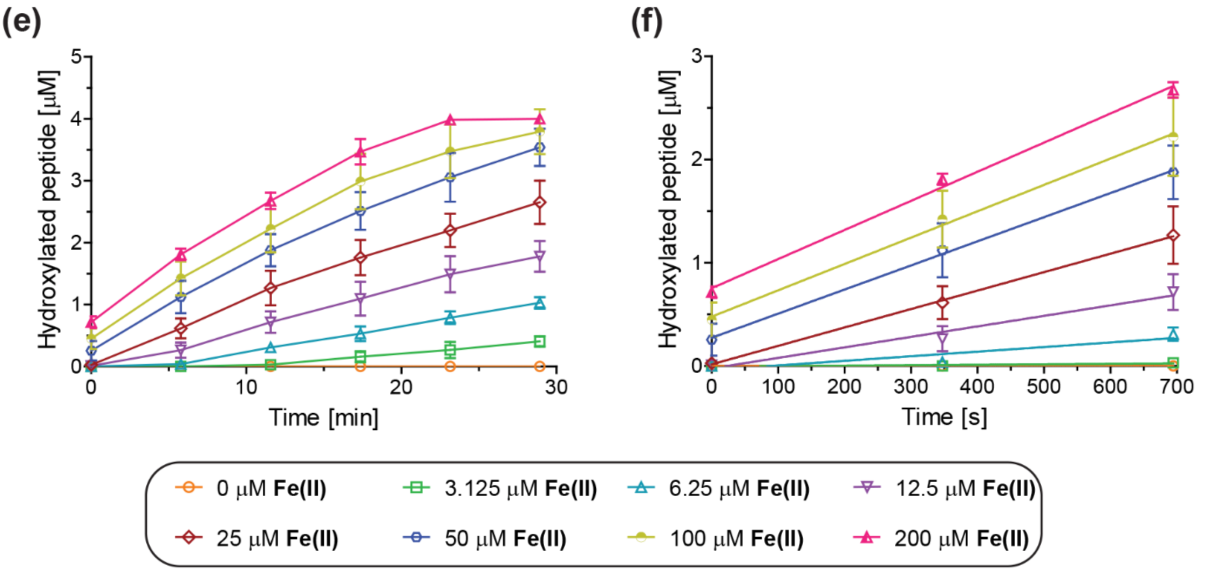

Supporting Figure S7. Reaction rates for determining kinetic parameters of R735Q AspH (continues on the following page). Maximum velocities (v_{max}^{app}) and apparent Michaelis constants (K_{m}^{app}) of isolated recombinant R735Q AspH were determined for 2OG and Fe(II), monitoring the R735Q AspH-catalyzed hydroxylation of hFX-EGFD1$_{86-124}$-4S (Supporting Figure S1c) by SPE-MS as described (2, 9). Conditions: R735Q His$_6$-AspH$_{315-758}$ (0.2 µM), hFX-EGFD1$_{86-124}$-4S (4.0 µM), and the shown concentrations of 2OG, L-ascorbic acid (LAA), and $(NH_4)_2Fe(SO_4)_2{\cdot}6H_2O$ in buffer (25 mM MES, pH 6.0, 20 °C). Measurement times were normalized to the first sample injection analyzed after the addition of R735Q AspH to the Substrate Mixture (t = 0 s), by which time low levels of substrate oxidation were manifest. Data are means of independent triplicates (n = 3; mean ± SD).

(**a**) Time course of the R735Q AspH-catalyzed oxidation of hFX-EGFD1$_{86-124}$-4S (Supporting Figure S1c) for the shown concentrations of LAA using 2OG (2000 µM) and $(NH_4)_2Fe(SO_4)_2{\cdot}6H_2O$ (100 µM); (**b**) oxidation rates used to determine kinetic parameters of R735Q AspH for LAA.

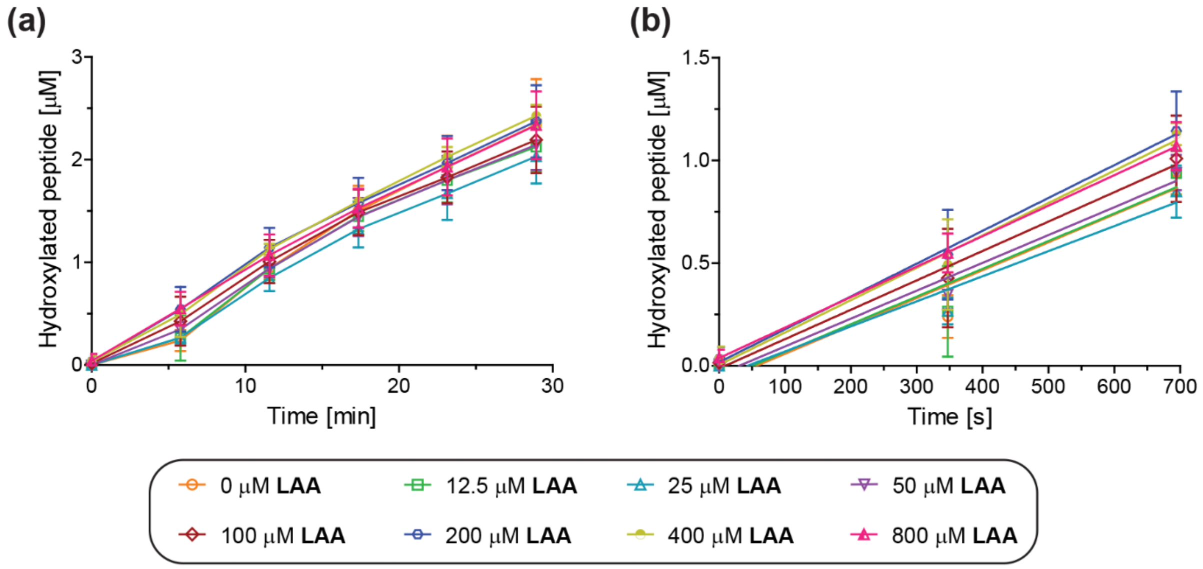

(**c**) Time course of the R735Q AspH-catalyzed oxidation of hFX-EGFD1$_{86-124}$-4S (Supporting Figure S1c) for the shown concentrations of 2OG using LAA (400 μM) and $(NH_4)_2Fe(SO_4)_2 \cdot 6H_2O$ (100 μM); (**d**) oxidation rates used to determine kinetic parameters of R735Q AspH for 2OG.

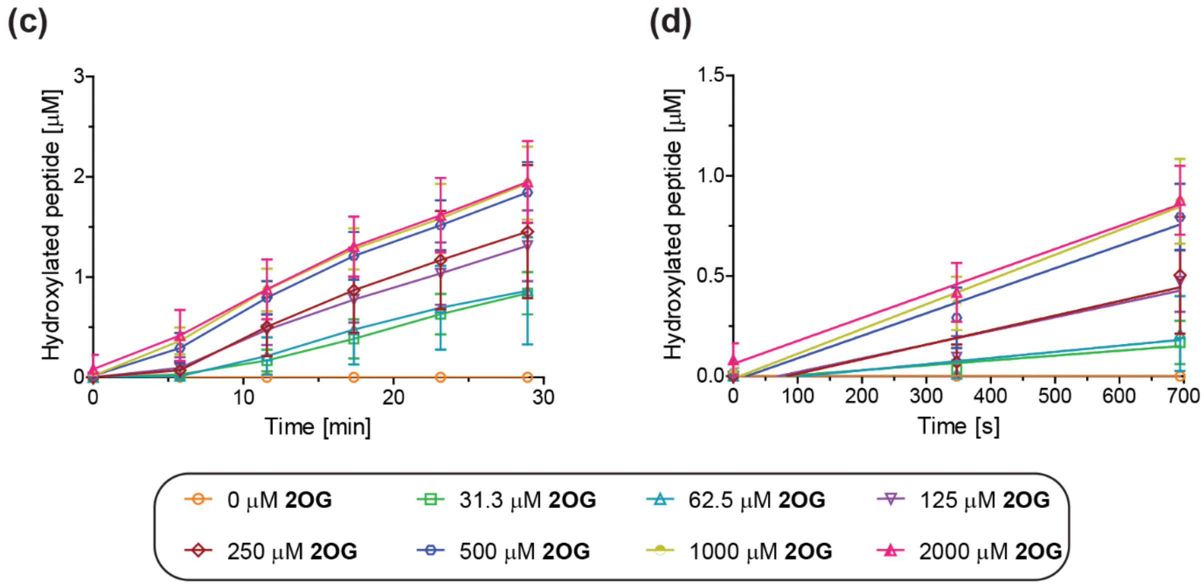

(**e**) Time course of the R735Q AspH-catalyzed oxidation of hFX-EGFD1$_{86-124}$-4S (Supporting Figure S1c) for the shown concentrations of $(NH_4)_2Fe(SO_4)_2 \cdot 6H_2O$ using LAA (400 μM) and 2OG (2000 μM); (**f**) oxidation rates used to determine kinetic parameters of R735Q AspH for Fe(II).

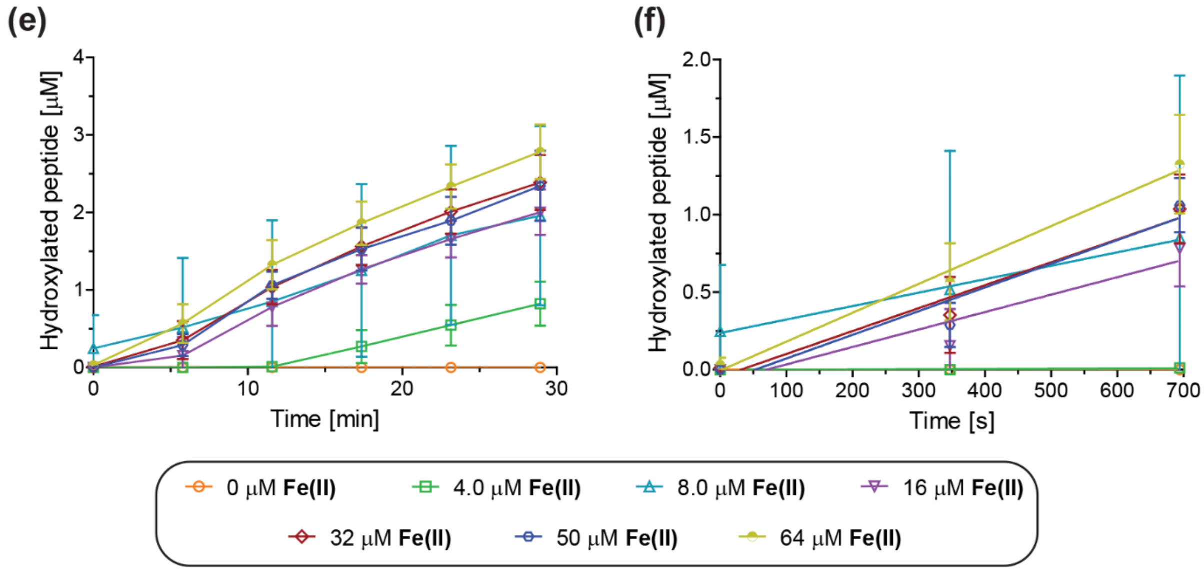

Supporting Figure S8. Views from a crystal structure of R735Q AspH complexed with 2OG, Mn, and a synthetic EGFD substrate peptide (R735Q AspH:2OG:Mn:hFX-EGFD1$_{86\text{-}124}$-4S; PDB ID: 8RE6). Colors: grey: R735Q His$_6$-AspH$_{315\text{-}758}$; teal: carbon-backbone of 2OG; lavendar: Mn; yellow: carbon-backbone of hFX-EGFD1$_{86\text{-}124}$-4S (Supporting Figure S1c); red: oxygen; blue: nitrogen; gold: sulfur.

(**a**) Overview of the R735Q AspH:2OG:Mn:hFX-EGFD1$_{86\text{-}124}$-4S crystal structure. (**b**) Representative Polder omit electron density map contoured to 3σ around the hFX-EGFD1$_{86\text{-}124}$-4S peptide from the R735Q AspH:2OG:Mn:hFX-EGFD1$_{86\text{-}124}$-4S structure reveals electron density for hFX-EGFD1$_{86\text{-}124}$-4S residues K100$_{hFX}$ to F116$_{hFX}$, including for the disulfide bridge between substrate residues C101$_{hFX}$ and C110$_{hFX}$.

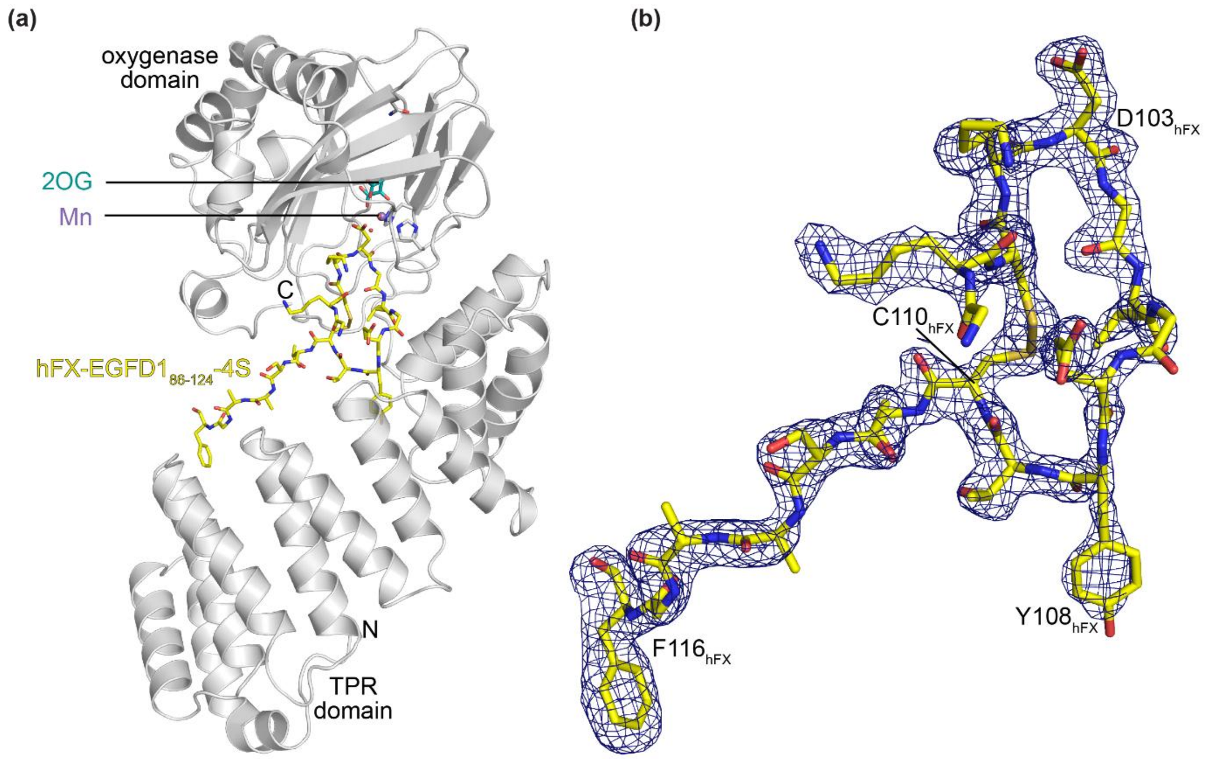

Supporting Figure S9. Views from a crystal structure of R735W AspH complexed with 2OG, Mn, and a synthetic EGFD substrate peptide (R735W AspH:2OG:Mn:hFX-EGFD1$_{86-124}$-4S; PDB ID: 8RE7). Colors: grey: R735W His$_6$-AspH$_{315-758}$; teal: carbon-backbone of 2OG; lavendar: Mn; yellow: carbon-backbone of hFX-EGFD1$_{86-124}$-4S (Supporting Figure S1c); red: oxygen; blue: nitrogen; gold: sulfur.
(**a**) Overview of the R735W AspH:2OG:Mn:hFX-EGFD1$_{86-124}$-4S crystal structure. (**b**) Representative Polder omit electron density map contoured to 3σ around the hFX-EGFD1$_{86-124}$-4S peptide from the R735W AspH:2OG:Mn:hFX-EGFD1$_{86-124}$-4S structure reveals electron density for hFX-EGFD1$_{86-124}$-4S residues G99$_{hFX}$ to F116$_{hFX}$, including for the disulfide bridge between substrate residues C101$_{hFX}$ and C110$_{hFX}$.

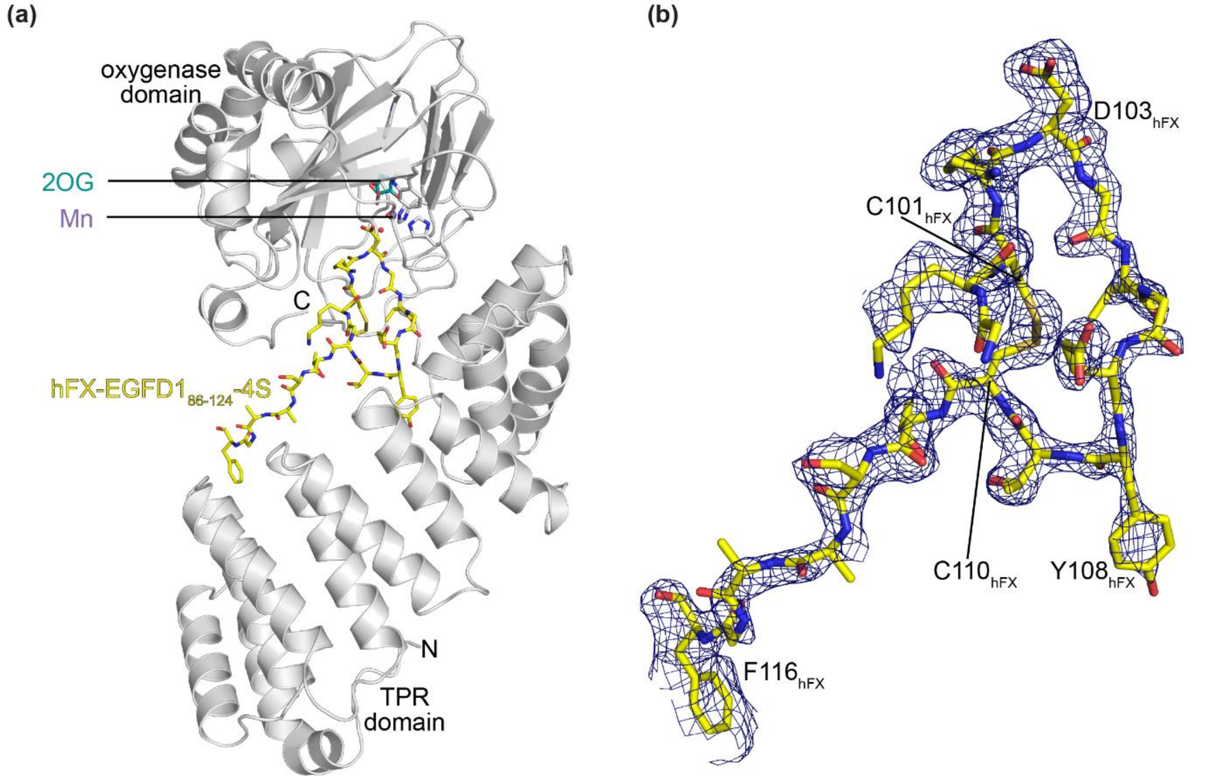

Supporting Figure S10. Views from a crystal structure of wt AspH complexed with 2OG, Mn, and a synthetic EGFD substrate peptide (wt AspH:2OG:Mn:hFX-EGFD1$_{86\text{-}124}$-4S; PDB ID: 8RE9). Colors: grey: wt AspH$_{315\text{-}758}$; teal: carbon-backbone of 2OG; lavendar: Mn; yellow: carbon-backbone of hFX-EGFD1$_{86\text{-}124}$-4S (Supporting Figure S1c); red: oxygen; blue: nitrogen; gold: sulfur.

(**a**) Overview of the wt AspH:2OG:Mn:hFX-EGFD1$_{86\text{-}124}$-4S crystal structure. (**b**) Representative Polder omit electron density map contoured to 3σ around the hFX-EGFD1$_{86\text{-}124}$-4S peptide from the wt AspH:2OG:Mn:hFX-EGFD1$_{86\text{-}124}$-4S structure reveals electron density for hFX-EGFD1$_{86\text{-}124}$-4S residues G99$_{hFX}$ to S112$_{hFX}$, including for the disulfide bridged (C101$_{hFX}$ and C110$_{hFX}$) ten-membered non-canonical EGFD macrocycle. Note that the D103$_{hFX}$ side-chain carboxylate of hFX-EGFD1$_{86\text{-}124}$-4S adopts two conformations in the improved resolution wt AspH:2OG:Mn:hFX-EGFD1$_{86\text{-}124}$-4S structure, as observed in some, but not all, reported AspH:substrate complex structures (1). The electron density observed for substrate residues L113$_{hFX}$ and E114$_{hFX}$ is relatively weak, and their modelled conformations should therefore be regarded as tentative.

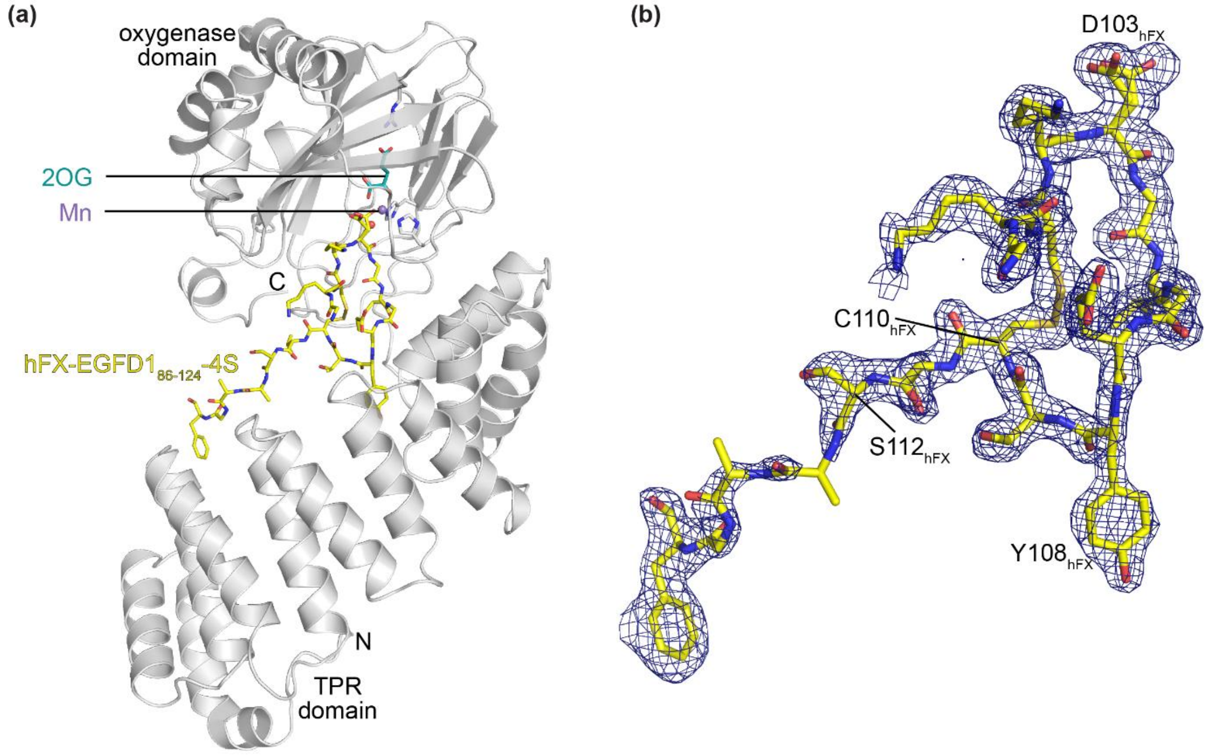

Supporting Figure S11. 2OG occupies a different conformation in the improved resolution wt AspH:2OG:Mn:hFX-EGFD1$_{86\text{-}124}$-4S complex structure than in that reported (continues on the following page). Colors: grey: wt AspH$_{315\text{-}758}$; teal: carbon-backbone of 2OG; lavendar: Mn; yellow: carbon-backbone of hFX-EGFD1$_{86\text{-}124}$-4S (Supporting Figure S1c); red: oxygen; blue: nitrogen; gold: sulfur. w: water.

(**a** and **b**) Superimposition of a view from the improved resolution wt AspH:2OG:Mn:hFX-EGFD1$_{86\text{-}124}$-4S structure (Supporting Figure S10) with one from a reported structure of wt His$_6$-AspH:2OG:Mn:hFX-EGFD1$_{86\text{-}124}$-4S (wt His$_6$-AspH: brown, Mn: pink, carbon-backbone of 2OG: orange, carbon-backbone of hFX-EGFD1$_{86\text{-}124}$-4S: green; PDB ID: 6YYW (10)) reveals similar: (**a**) AspH conformations (Cα RMSD ~ 0.27 Å) and (**b**) hFX-EGFD1$_{86\text{-}124}$-4S conformations (Cα RMSD ~ 0.20 Å), in particular of the residues forming the disulfide bridged (C101$_{hFX}$ and C110$_{hFX}$) ten-membered non-canonical EGFD macrocycle. Note that the D103$_{hFX}$ side-chain carboxylate of hFX-EGFD1$_{86\text{-}124}$-4S adopts two conformations in the improved resolution wt AspH:2OG:Mn:hFX-EGFD1$_{86\text{-}124}$-4S structure, but not in the reported wt His$_6$-AspH:2OG:Mn:hFX-EGFD1$_{86\text{-}124}$-4S structure (PDB ID: 6YYW (10)). (**c**) Superimposition of an active site view from the improved resolution wt AspH:2OG:Mn:hFX-EGFD1$_{86\text{-}124}$-4S structure (Supporting Figure S10) with one from the reported wt His$_6$-AspH:2OG:Mn:hFX-EGFD1$_{86\text{-}124}$-4S (wt His$_6$-AspH: brown, Mn: pink, carbon-backbone of 2OG: orange, carbon-backbone of hFX-EGFD1$_{86\text{-}124}$-4S: green; PDB ID: 6YYW (10)) reveals that the side chains of active site residues involved in 2OG or metal binding adopt similar conformations in both structures. (**d**) Superimposition of a view of 2OG from the improved resolution wt AspH:2OG:Mn:hFX-EGFD1$_{86\text{-}124}$-4S structure (Supporting Figure S10) with one from our reported wt His$_6$-AspH:2OG:Mn:hFX-EGFD1$_{86\text{-}124}$-4S structure (wt His$_6$-AspH: brown, Mn: pink, carbon-backbone of 2OG: orange, carbon-backbone of hFX-EGFD1$_{86\text{-}124}$-4S: green; PDB ID: 6YYW (10)) reveals that the C-1, C-2, and C-5 carbons occupy similar conformations, whereas those of the C-3 and C-4 carbons substantially differ. The dihedral angle formed by the 2OG C-1/C-2/C-3/C-4 atoms is 64º in the improved resolution wt AspH:2OG:Mn:hFX-EGFD1$_{86\text{-}124}$-4S structure, indicative of a *gauche* conformation around the 2OG C-2/C-3 bond, but is -170º in the reported wt His$_6$-AspH:2OG:Mn:hFX-EGFD1$_{86\text{-}124}$-4S structure (PDB ID: 6YYW (10)), indicative of an *anti-periplanar* conformation around the 2OG C-2/C-3 bond, an observation which may in part reflect the enhanced resolution of the improved resolution wt AspH:2OG:Mn:hFX-EGFD1$_{86\text{-}124}$-4S structure allowing to define the 2OG conformation more accurately than in the reported structure.

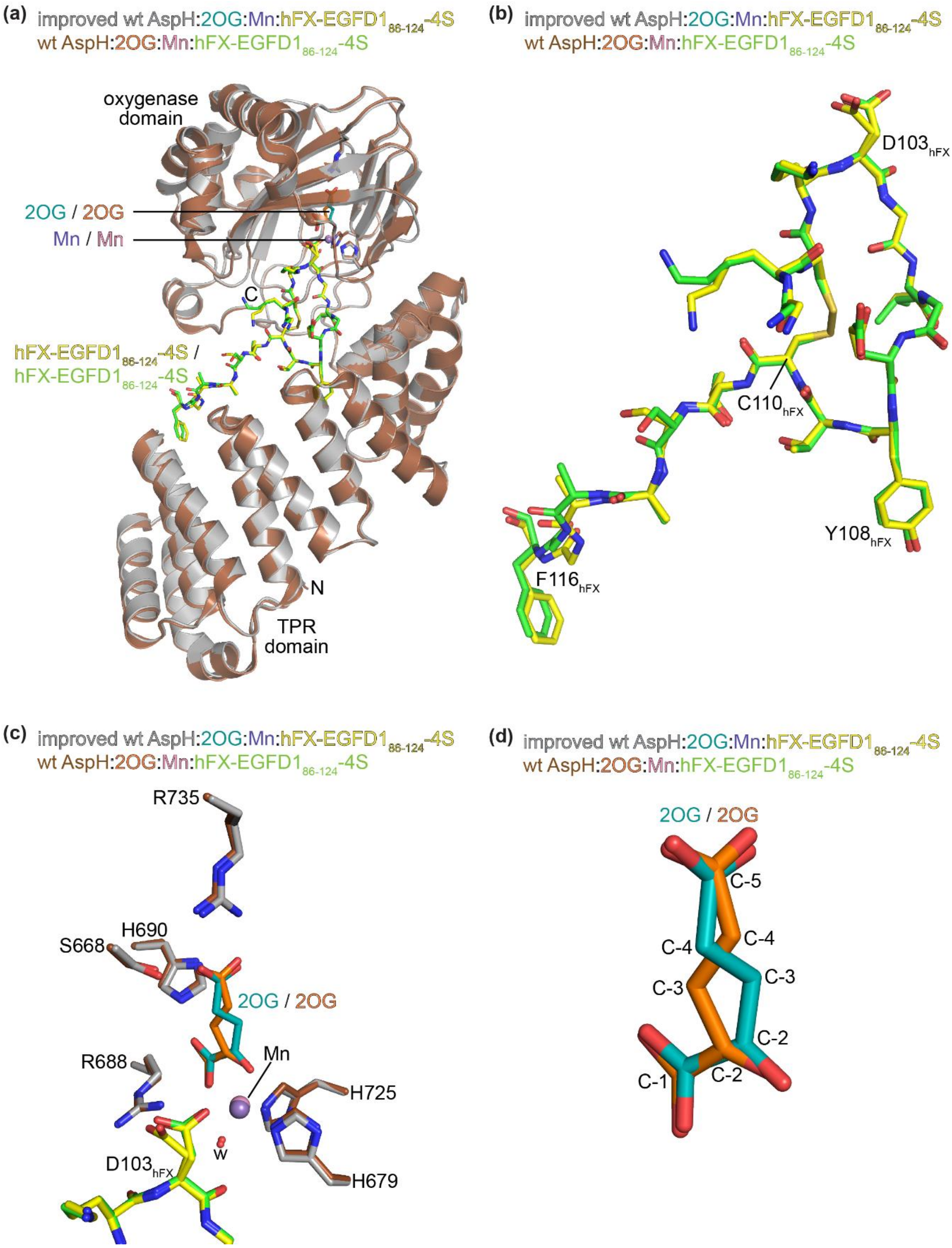

(a)
improved wt AspH:2OG:Mn:hFX-EGFD1$_{86-124}$-4S
wt AspH:2OG:Mn:hFX-EGFD1$_{86-124}$-4S
oxygenase domain
2OG / 2OG
Mn / Mn
C
hFX-EGFD1$_{86-124}$-4S /
hFX-EGFD1$_{86-124}$-4S
N
TPR domain
(b)
improved wt AspH:2OG:Mn:hFX-EGFD1$_{86-124}$-4S
wt AspH:2OG:Mn:hFX-EGFD1$_{86-124}$-4S
D103$_{hFX}$
C110$_{hFX}$
Y108$_{hFX}$
F116$_{hFX}$
(c)
improved wt AspH:2OG:Mn:hFX-EGFD1$_{86-124}$-4S
wt AspH:2OG:Mn:hFX-EGFD1$_{86-124}$-4S
R735
H690
S668
2OG / 2OG
R688
Mn
H725
w
D103$_{hFX}$
H679
(d)
improved wt AspH:2OG:Mn:hFX-EGFD1$_{86-124}$-4S
wt AspH:2OG:Mn:hFX-EGFD1$_{86-124}$-4S
2OG / 2OG
C-5
C-4
C-4
C-3
C-3
C-2
C-1
C-2

Supporting Figure S12. R735Q AspH and wt AspH adopt similar folds in complex with Mn, 2OG, and a substrate peptide. Colors: grey: R735Q His$_6$-AspH$_{315-758}$; teal: carbon-backbone of 2OG; lavendar: Mn; yellow: carbon-backbone of hFX-EGFD1$_{86-124}$-4S (Supporting Figure S1c); red: oxygen; blue: nitrogen; gold: sulfur.

(**a** and **b**) Superimposition of a view from the R735Q AspH:2OG:Mn:hFX-EGFD1$_{86-124}$-4S structure (Supporting Figure S8; PDB ID: 8RE6) with one from the improved resolution wt AspH:2OG:Mn:hFX-EGFD1$_{86-124}$-4S structure (wt AspH: brown, Mn: pink, carbon-backbone of 2OG: orange, carbon-backbone of hFX-EGFD1$_{86-124}$-4S: green; PDB ID: 8RE9; Supporting Figure S10) reveals similar: (**a**) AspH conformations (Cα RMSD ~ 0.30 Å) and (**b**) hFX-EGFD1$_{86-124}$-4S conformations (Cα RMSD ~ 0.24 Å), in particular of the residues forming the disulfide bridged (C101$_{hFX}$ and C110$_{hFX}$) ten-membered non-canonical EGFD macrocycle. Notably, the D103$_{hFX}$ side-chain carboxylate of hFX-EGFD1$_{86-124}$-4S was modelled with one conformation in the R735Q AspH:2OG:Mn:hFX-EGFD1$_{86-124}$-4S structure (PDB ID: 8RE6), but with two conformations in the improved resolution wt AspH:2OG:Mn:hFX-EGFD1$_{86-124}$-4S structure (PDB ID: 8RE9).

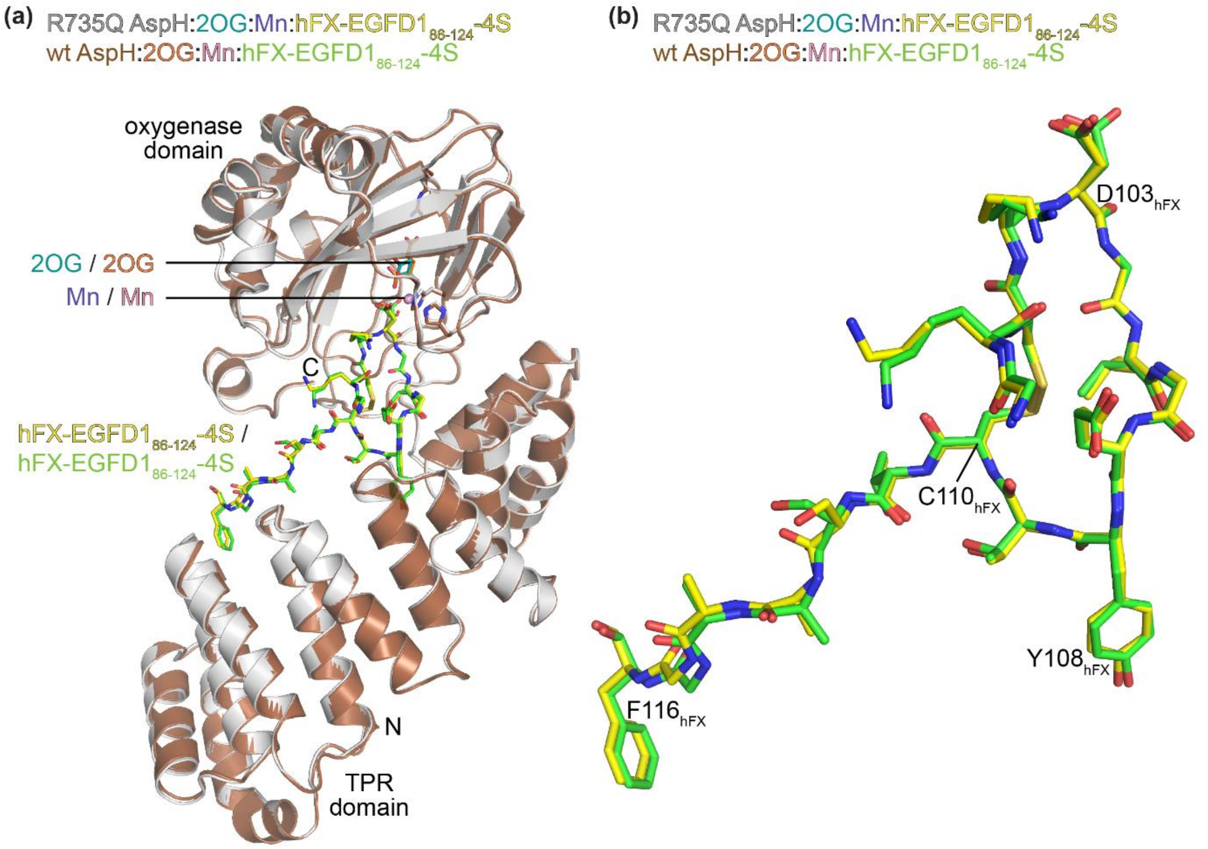

Supporting Figure S13. R735W AspH and wt AspH adopt similar folds in complex with Mn, 2OG, and a substrate peptide. Colors: grey: R735W His$_6$-AspH$_{315-758}$; teal: carbon-backbone of 2OG; lavendar: Mn; yellow: carbon-backbone of hFX-EGFD1$_{86-124}$-4S (Supporting Figure S1c); red: oxygen; blue: nitrogen; gold: sulfur.
(**a** and **b**) Superimposition of a view from the R735W AspH:2OG:Mn:hFX-EGFD1$_{86-124}$-4S structure (Supporting Figure S9; PDB ID: 8RE7) with one from the improved resolution wt AspH:2OG:Mn:hFX-EGFD1$_{86-124}$-4S structure (wt AspH: brown, Mn: pink, carbon-backbone of 2OG: orange, carbon-backbone of hFX-EGFD1$_{86-124}$-4S: green; PDB ID: 8RE9; Supporting Figure S10) reveals similar: (**a**) AspH conformations (Cα RMSD ~ 0.30 Å) and (**b**) hFX-EGFD1$_{86-124}$-4S conformations (Cα RMSD ~ 0.20 Å), in particular of the residues forming the disulfide bridged (C101$_{hFX}$ and C110$_{hFX}$) ten-membered non-canonical EGFD macrocycle. Notably, the D103$_{hFX}$ side-chain carboxylate of hFX-EGFD1$_{86-124}$-4S was modelled with one conformation in the R735W AspH:2OG:Mn:hFX-EGFD1$_{86-124}$-4S structure (PDB ID: 8RE7) but with two conformations in the improved resolution wt AspH:2OG:Mn:hFX-EGFD1$_{86-124}$-4S structure (PDB ID: 8RE9).

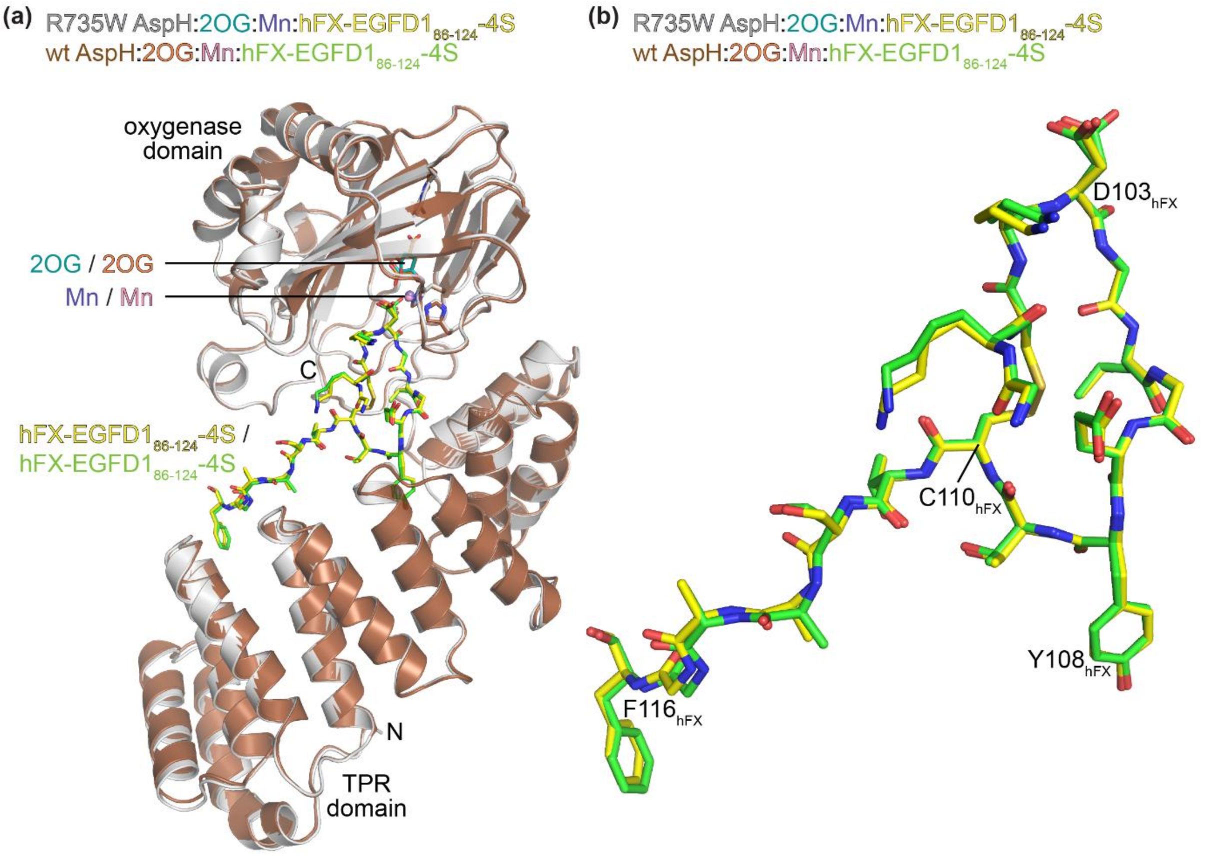

Supporting Figure S14. R735W and R735Q AspH adopt similar folds in complex with Mn, 2OG, and a substrate peptide. Colors: grey: R735W His$_6$-AspH$_{315-758}$; teal: carbon-backbone of 2OG; lavendar: Mn; yellow: carbon-backbone of hFX-EGFD1$_{86-124}$-4S (Supporting Figure S1c); red: oxygen; blue: nitrogen; gold: sulfur.
(**a** and **b**) Superimposition of a view from the R735W AspH:2OG:Mn:hFX-EGFD1$_{86-124}$-4S structure (Supporting Figure S9; PDB ID: 8RE7) with one from the R735Q AspH:2OG:Mn:hFX-EGFD1$_{86-124}$-4S structure (R735Q AspH: ochre, Mn: pink, carbon-backbone of 2OG: orange, carbon-backbone of hFX-EGFD1$_{86-124}$-4S: cyan; PDB ID: 8RE6; Supporting Figure S8) reveals similar: (**a**) AspH conformations (Cα RMSD ~ 0.29 Å) and (**b**) hFX-EGFD1$_{86-124}$-4S conformations (Cα RMSD ~ 0.14 Å), in particular of the residues forming the disulfide bridged (C101$_{hFX}$ and C110$_{hFX}$) ten-membered non-canonical EGFD substrate macrocycle.

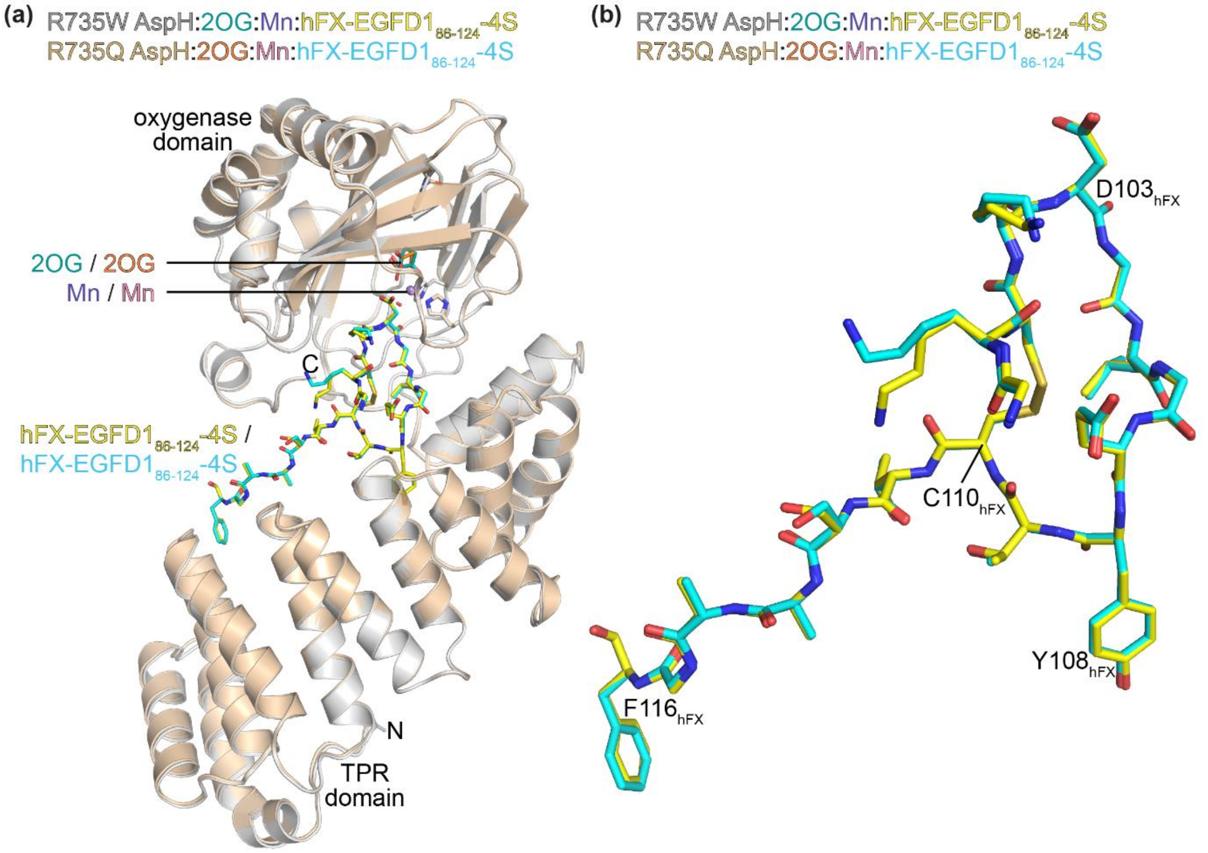

Supporting Figure S15. Rates and kinetic parameters for the reaction of wt AspH with 2OG derivatives (continues on the following page). Maximum velocities (v_{max}^{app}) and apparent Michaelis constants (K_{m}^{app}) of isolated recombinant wt AspH were determined for 3-methyl-2OG (**1**), (1*R*)-3-(carboxycarbonyl)cyclopentane-1-carboxylic acid (**5**), 2-bromo-4-carboxyphenylglyoxylic acid (**8**), and 2-oxoadipic acid (2OA, **10**), monitoring the wt AspH-catalyzed hydroxylation of hFX-EGFD1$_{86\text{-}124}$-4S (Supporting Figure S1c) by SPE-MS as described (2, 9). Conditions: wt AspH$_{315\text{-}758}$ (0.1 µM), hFX-EGFD1$_{86\text{-}124}$-4S (4.0 µM), L-ascorbic acid (100 µM), $(NH_4)_2Fe(SO_4)_2{\cdot}6H_2O$ (20 µM), and the shown 2OG derivative concentrations in buffer (25 mM HEPES, pH 7.5, 50 mM NaCl, 20 °C). Measurement times were normalized to the first sample injection analyzed after the addition of wt AspH to the Substrate Mixture (t = 0 s), by which time low levels of substrate oxidation were manifest. Data are means of independent triplicates (n = 3; mean ± SD).

(**a**) Time course of the wt AspH-catalyzed oxidation of hFX-EGFD1$_{86\text{-}124}$-4S (Supporting Figure S1c) for the shown concentrations of 3-methyl-2OG (**1**); (**b**) oxidation rates used to determine kinetic parameters of wt AspH for **1**; (**c**) determination of the wt AspH v_{max}^{app} and K_{m}^{app} values for **1** (*i.e.*, 12.7 ± 0.1 nM·s^{-1} and 1.6 ± 0.4 µM, respectively).

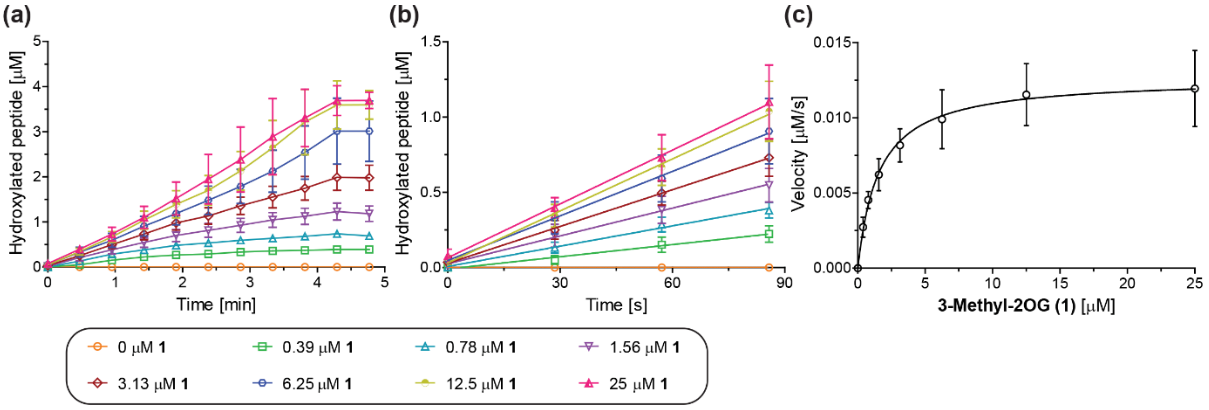

(**d**) Time course of the wt AspH-catalyzed oxidation of hFX-EGFD1$_{86\text{-}124}$-4S (Supporting Figure S1c) for the shown concentrations of (1*R*)-3-(carboxycarbonyl)cyclopentane-1-carboxylic acid (**5**); (**e**) oxidation rates used to determine kinetic parameters of wt AspH for **5**; (**f**) determination of the wt AspH v_{max}^{app} and K_{m}^{app} values for **5** (*i.e.*, 7.3 ± 0.2 nM·s^{-1} and 8.2 ± 0.6 µM, respectively).

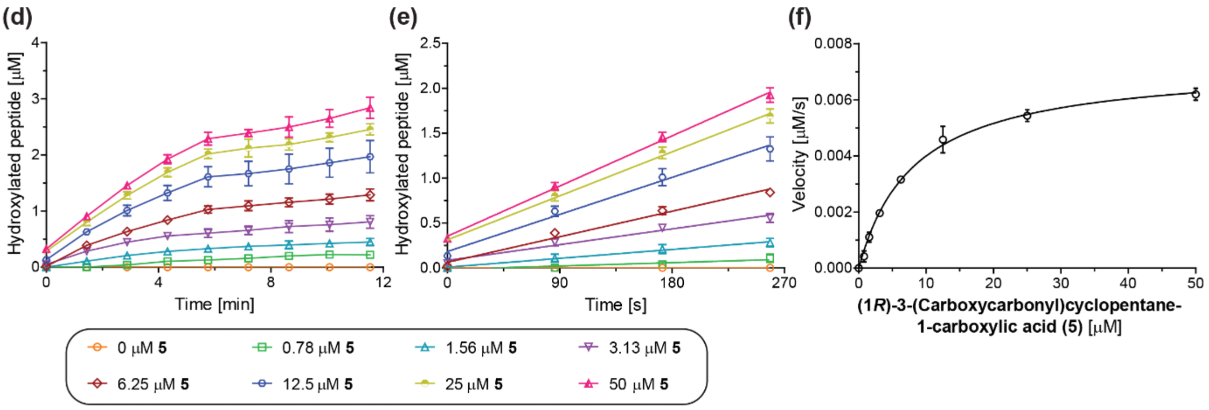

(**g**) Time course of the wt AspH-catalyzed oxidation of hFX-EGFD1$_{86\text{-}124}$-4S (Supporting Figure S1c) for the shown concentrations of 2-bromo-4-carboxyphenylglyoxylic acid (**8**); (**h**) oxidation rates used to determine kinetic parameters of wt AspH for **8**; (**i**) determination of the wt AspH v_{max}^{app} and K_m^{app} values for **8** (*i.e.*, 8.9 ± 0.1 nM·s^{-1} and 120 ± 17 μM, respectively).

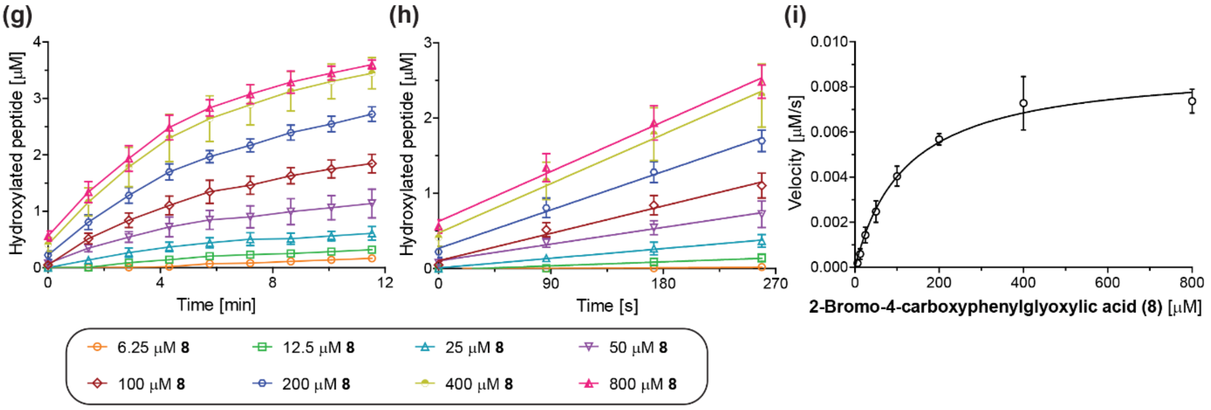

(**j**) Time course of the wt AspH-catalyzed oxidation of hFX-EGFD1$_{86\text{-}124}$-4S (Supporting Figure S1c) for the shown concentrations of 2-oxoadipic acid (2OA, **10**); (**k**) oxidation rates used to determine kinetic parameters of wt AspH for **10**; (**l**) determination of the wt AspH v_{max}^{app} and K_m^{app} values for **10** (*i.e.*, 11.2 ± 0.5 nM·s^{-1} and 4.7 ± 0.7 μM, respectively).

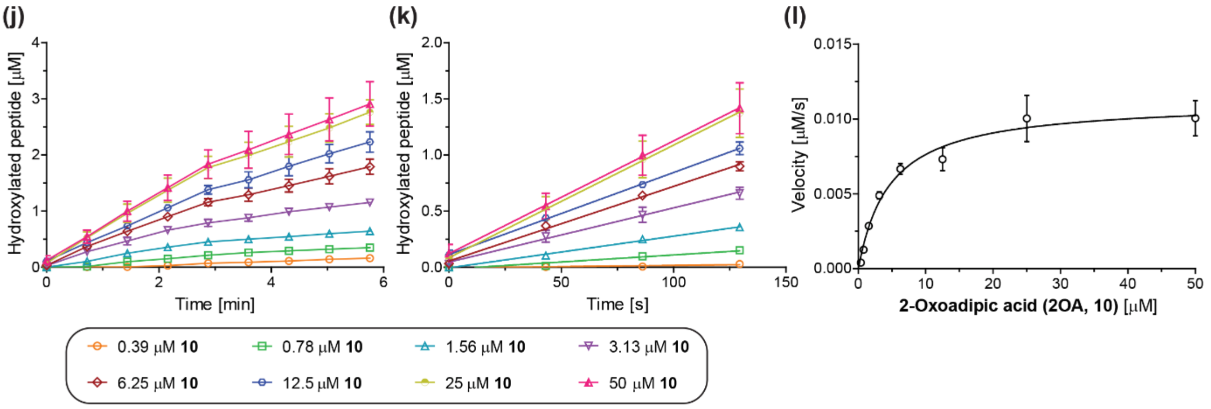

Supporting Figure S16. Rates and kinetic parameters for the reaction of G434V AspH with 3-methyl-2OG. Maximum velocities (v_{max}^{app}) and apparent Michaelis constants (K_m^{app}) of isolated recombinant G434V AspH were determined for 3-methyl-2OG (**1**), monitoring the G434V AspH-catalyzed hydroxylation of hFX-EGFD1$_{86\text{-}124}$-4S (Supporting Figure S1c) by SPE-MS as described (2, 9). Conditions: G434V His$_6$-AspH$_{315\text{-}758}$ (0.1 µM), hFX-EGFD1$_{86\text{-}124}$-4S (4.0 µM), L-ascorbic acid (100 µM), $(NH_4)_2Fe(SO_4)_2{\cdot}6H_2O$ (20 µM), and the shown concentrations of **1** in buffer (25 mM HEPES, pH 7.5, 50 mM NaCl, 20 °C). Measurement times were normalized to the first sample injection analyzed after the addition of G434V AspH to the Substrate Mixture (t = 0 s), by which time low levels of substrate oxidation were manifest. Data are means of independent triplicates (n = 3; mean ± SD).

(**a**) Time course of the G434V AspH-catalyzed oxidation of hFX-EGFD1$_{86\text{-}124}$-4S (Supporting Figure S1c) for the shown concentrations of 3-methyl-2OG (**1**); (**b**) oxidation rates used to determine kinetic parameters of G434V AspH for **1**; (**c**) determination of the G434V AspH v_{max}^{app} and K_m^{app} values for **1** (*i.e.*, 19.5 ± 0.5 nM·s^{-1} and 2.3 ± 0.3 µM, respectively).

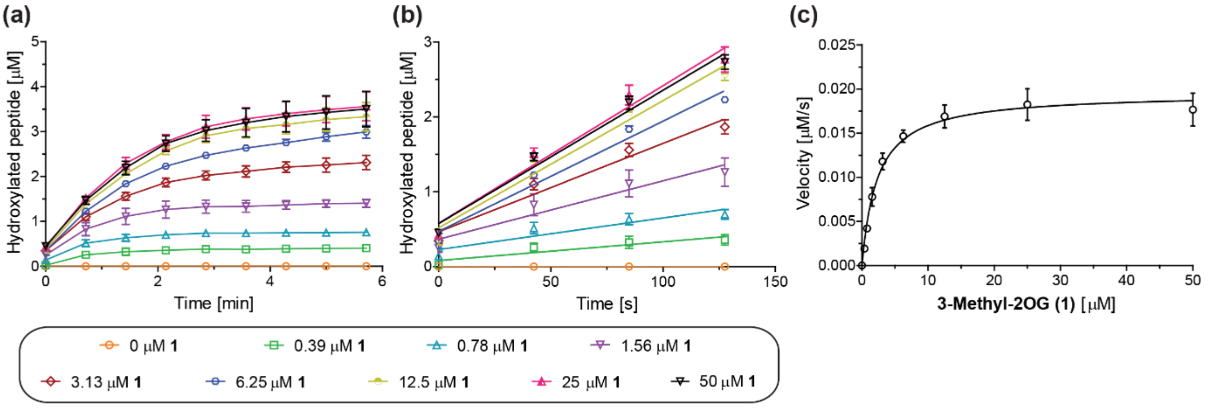

Supporting Figure S17. Rates and kinetic parameters for the reaction of R688Q AspH with 3-methyl-2OG. Maximum velocities (v_{max}^{app}) and apparent Michaelis constants (K_m^{app}) of isolated recombinant R688Q AspH were determined for 3-methyl-2OG (**1**), monitoring the R688Q AspH-catalyzed hydroxylation of hFX-EGFD1$_{86\text{-}124}$-4S (Supporting Figure S1c) by SPE-MS as described (2, 9). Conditions: R688Q His$_6$-AspH$_{315\text{-}758}$ (0.2 µM), hFX-EGFD1$_{86\text{-}124}$-4S (4.0 µM), *L*-ascorbic acid (400 µM), $(NH_4)_2Fe(SO_4)_2{\cdot}6H_2O$ (100 µM), and the shown concentrations of **1** in buffer (25 mM MES, pH 6.0, 20 °C). Measurement times were normalized to the first sample injection analyzed after the addition of R688Q AspH to the Substrate Mixture (t = 0 s), by which time low levels of substrate oxidation were manifest. Data are means of independent triplicates (n = 3; mean ± SD).

(**a**) Time course of the R688Q AspH-catalyzed oxidation of hFX-EGFD1$_{86\text{-}124}$-4S (Supporting Figure S1c) for the shown concentrations of 3-methyl-2OG (**1**); (**b**) oxidation rates used to determine kinetic parameters of R688Q AspH for **1**; (**c**) determination of the R688Q AspH v_{max}^{app} and K_m^{app} values for **1** (*i.e.*, 5.6 ± 0.2 nM·s^{-1} and 2.5 ± 0.4 µM, respectively).

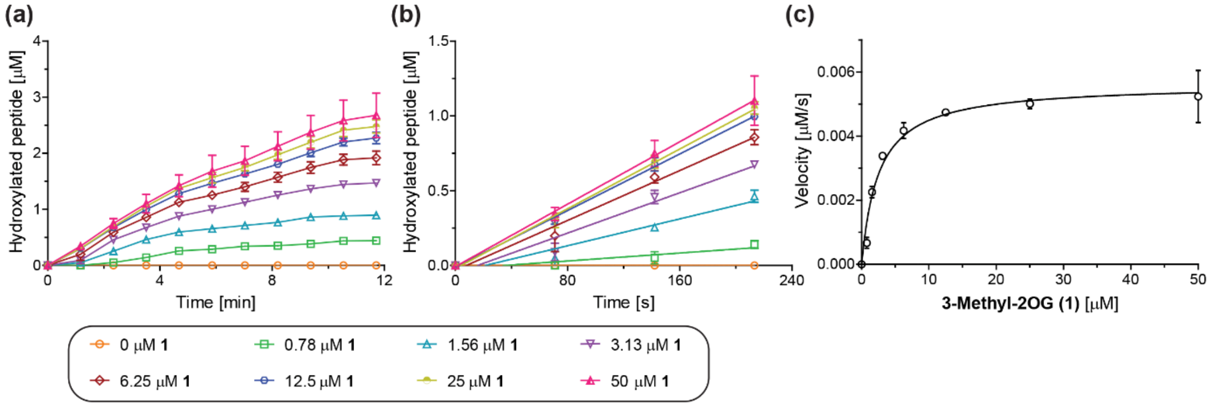

Supporting Figure S18. Rates and kinetic parameters for the reaction of R735Q AspH with 2OG derivatives (continues on the 2 following pages). Maximum velocities (v_{max}^{app}) and apparent Michaelis constants (K_m^{app}) of isolated recombinant R735Q AspH were determined for (1*R*)-3-(carboxycarbonyl)cyclopentane-1-carboxylic acid (**5**), 4-(carboxycarbonyl)bicyclo[2.2.2]octane-1-carboxylic acid (**6**), *trans*-4-(carboxycarbonyl)cyclohexane-1-carboxylic acid (**42**), 2-oxoadipic acid (2OA, **10**), 2-oxopimelic acid (2OP, **11**), and 2-oxosuberic acid (2OS, **12**), monitoring the R735Q AspH-catalyzed hydroxylation of hFX-EGFD1$_{86\text{-}124}$-4S (Supporting Figure S1c) by SPE-MS as described (2, 9). Conditions: R735Q His$_6$-AspH$_{315\text{-}758}$ (0.1 μM), hFX-EGFD1$_{86\text{-}124}$-4S (4.0 μM), L-ascorbic acid (400 μM), $(NH_4)_2Fe(SO_4)_2 \cdot 6H_2O$ (200 μM), and the shown 2OG derivative concentrations in buffer (25 mM MES, pH 6.0, 20 °C). Measurement times were normalized to the first sample injection analyzed after the addition of R735Q AspH to the Substrate Mixture (t = 0 s), by which time low levels of substrate oxidation were manifest. Data are means of independent triplicates (n = 3; mean ± SD).

(**a**) Time course of the R735Q AspH-catalyzed oxidation of hFX-EGFD1$_{86\text{-}124}$-4S (Supporting Figure S1c) for the shown concentrations of (1*R*)-3-(carboxycarbonyl)cyclopentane-1-carboxylic acid (**5**); (**b**) oxidation rates used to determine kinetic parameters of R735Q AspH for **5**; (**c**) determination of the R735Q AspH v_{max}^{app} and K_m^{app} values for **5** (*i.e.*, 0.8 ± 0.1 nM·s^{-1} and 170 ± 35 μM, respectively). Note that application of a substrate inhibition model did not improve the fit or alter kinetic parameters substantially.

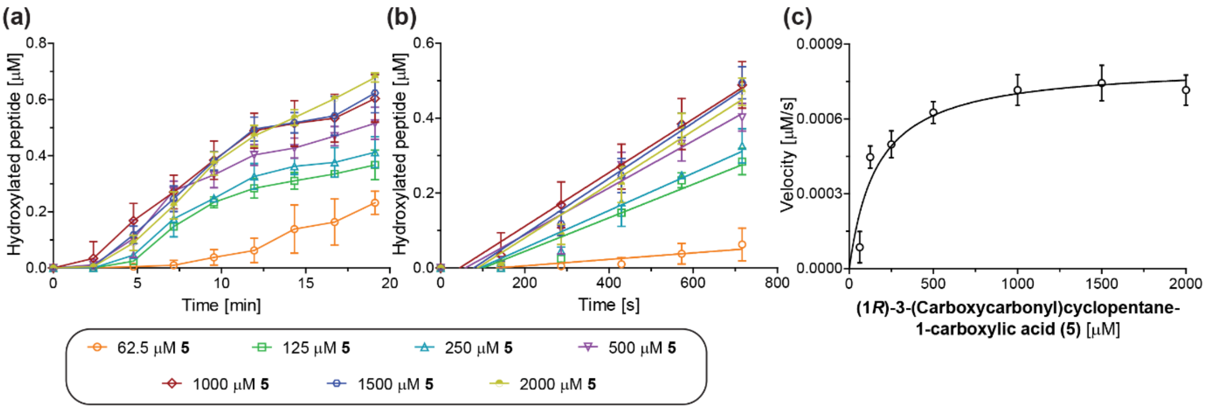

(**d**) Time course of the R735Q AspH-catalyzed oxidation of hFX-EGFD1$_{86\text{-}124}$-4S (Supporting Figure S1c) for the shown concentrations of 4-(carboxycarbonyl)bicyclo[2.2.2]octane-1-carboxylic acid (**6**); (**e**) oxidation rates used to determine kinetic parameters of R735Q AspH for **6**; (**f**) determination of the R735Q AspH v_{max}^{app} and K_m^{app} values for **6** (*i.e.*, 3.8 ± 0.4 nM·s^{-1} and 3000 ± 600 μM, respectively).

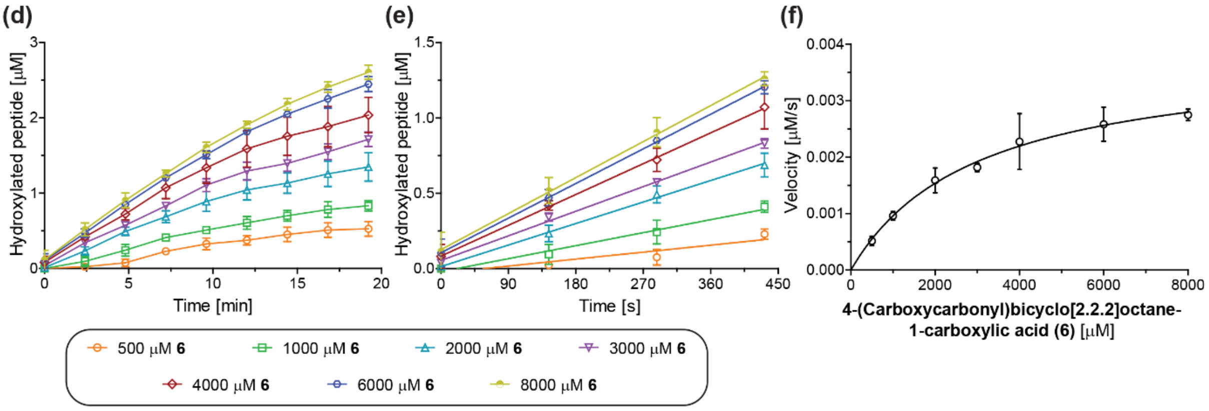

(**g**) Time course of the R735Q AspH-catalyzed oxidation of hFX-EGFD1$_{86-124}$-4S (Supporting Figure S1c) for the shown concentrations of *trans*-4-(carboxycarbonyl)cyclohexane-1-carboxylic acid (**42**); (**h**) oxidation rates used to determine kinetic parameters of R735Q AspH for **42**; (**i**) determination of the R735Q AspH v_{max}^{app} and K_{m}^{app} values for **42** (*i.e.*, 1.8 ± 0.1 nM·s^{-1} and 1100 ± 150 µM, respectively).

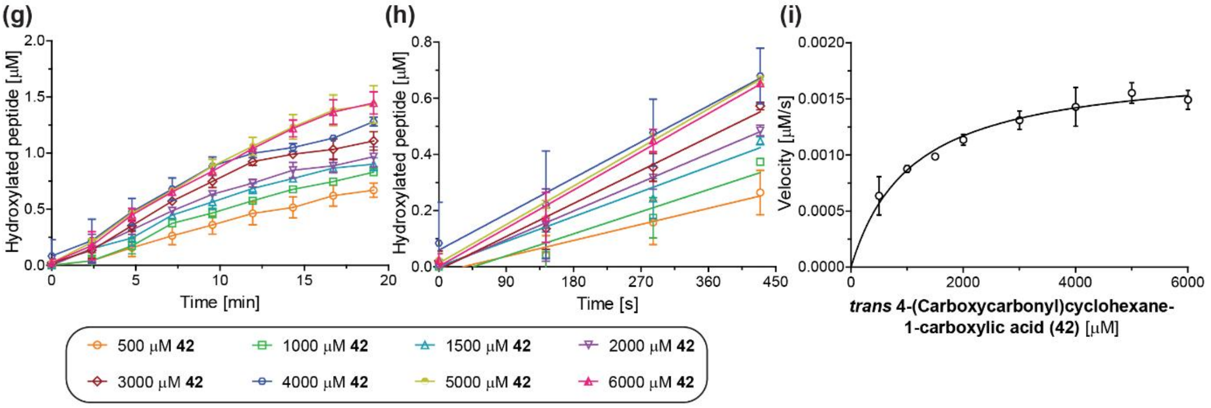

(**j**) Time course of the R735Q AspH-catalyzed oxidation of hFX-EGFD1$_{86-124}$-4S (Supporting Figure S1c) for the shown concentrations of 2-oxoadipic acid (2OA, **10**); (**k**) oxidation rates used to determine kinetic parameters of R735Q AspH for **10**; (**l**) determination of the R735Q AspH v_{max}^{app} and K_{m}^{app} values for **10** (*i.e.*, 2.9 ± 0.4 nM·s^{-1} and 85 ± 23 µM, respectively); note that the data were fitted using non-linear regression to an equation which accounts for substrate inhibition (Y = v_{max}^{app}•X/(K_{m}^{app}+X•(1+X/K_{i}))), as increased concentrations of **10** appear to impair R735Q AspH catalysis.

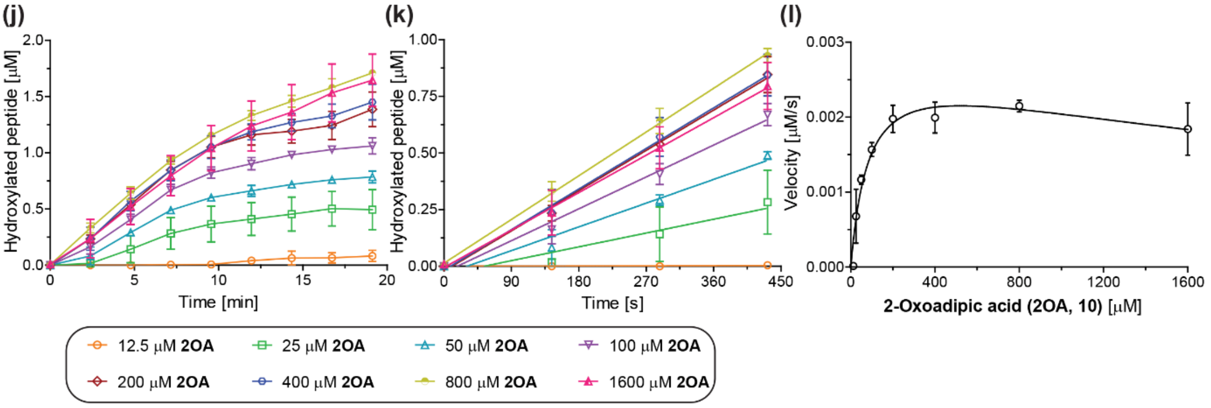

(**m**) Time course of the R735Q AspH-catalyzed oxidation of hFX-EGFD1$_{86\text{-}124}$-4S (Supporting Figure S1c) for the shown concentrations of 2-oxopimelic acid (2OP, **11**); (**n**) oxidation rates used to determine kinetic parameters of R735Q AspH for **11**; (**o**) determination of the R735Q AspH v_{max}^{app} and K_{m}^{app} values for **11** (*i.e.*, 37.5 ± 2.4 nM·s^{-1} and 550 ± 97 μM, respectively).

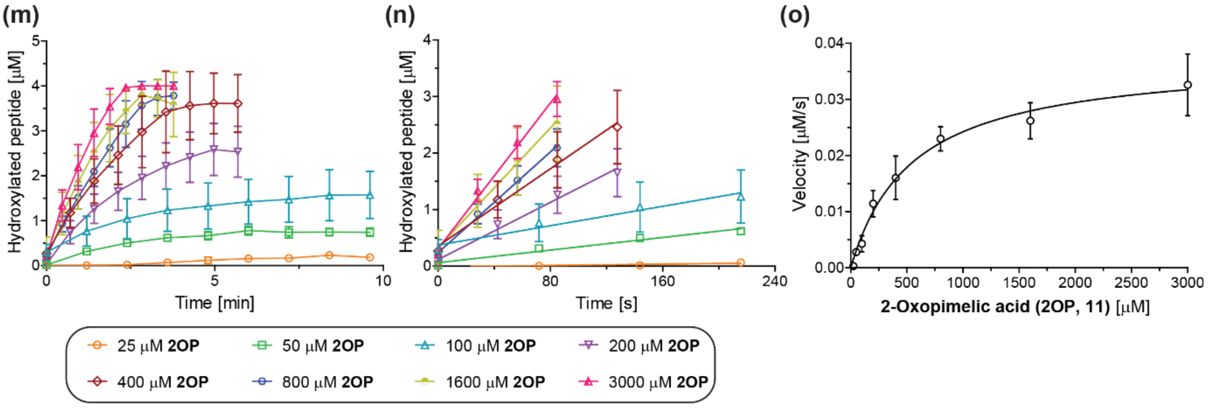

(**p**) Time course of the R735Q AspH-catalyzed oxidation of hFX-EGFD1$_{86\text{-}124}$-4S (Supporting Figure S1c) for the shown concentrations of 2-oxosuberic acid (2OS, **12**); (**q**) oxidation rates used to determine kinetic parameters of R735Q AspH for **12**; (**r**) determination of the R735Q AspH v_{max}^{app} and K_{m}^{app} values for **12** (*i.e.*, 72 ± 6.7 nM·s^{-1} and 640 ± 160 μM, respectively). Note that application of a substrate inhibition model did not improve the fit or alter kinetic parameters substantially.

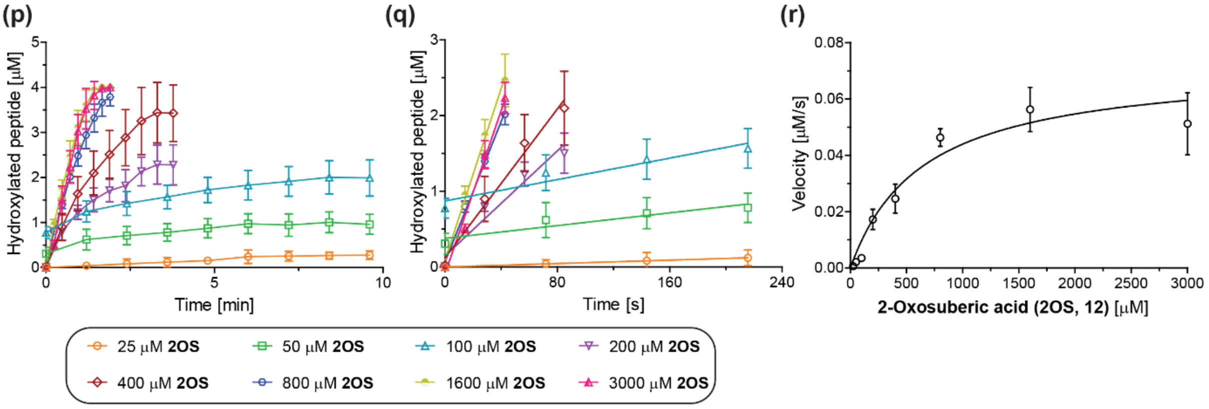

Supporting Figure S19. Rates and kinetic parameters for the reaction of R735Q AspH with hydrophobic 2-oxoacids (continues on the 3 following pages). Maximum velocities (v_{max}^{app}) and apparent Michaelis constants (K_{m}^{app}) of isolated recombinant R735Q AspH were determined for 3-methyl-2-oxobutanoic acid (**13**), (3*S*)-3-methyl-2-oxopentanoic acid (**15**), 4-methyl-2-oxopentanoic acid (**16**), 3-cyclohexyl-2-oxopropanoic acid (**18**), 2-oxo-4-phenylbutanoic acid (**20**), 2-cyclohexyl-2-oxoacetic acid (**53**), and 2-oxopentanoic acid (**54**), monitoring the R735Q AspH-catalyzed hydroxylation of hFX-EGFD1$_{86\text{-}124}$-4S (Supporting Figure S1c) by SPE-MS as described (2, 9). Conditions: R735Q His$_6$-AspH$_{315\text{-}758}$ (0.1 µM), hFX-EGFD1$_{86\text{-}124}$-4S (4.0 µM), *L*-ascorbic acid (400 µM), $(NH_4)_2Fe(SO_4)_2\cdot 6H_2O$ (200 µM), and the shown 2-oxoacid concentrations in buffer (25 mM MES, pH 6.0, 20 °C). Measurement times were normalized to the first sample injection analyzed after the addition of R735Q AspH to the Substrate Mixture (t = 0 s), by which time low levels of substrate oxidation were manifest. Data are means of independent triplicates (n = 3; mean ± SD).

(**a**) Time course of the R735Q AspH-catalyzed oxidation of hFX-EGFD1$_{86\text{-}124}$-4S (Supporting Figure S1c) for the shown concentrations of 3-methyl-2-oxobutanoic acid (**13**); (**b**) oxidation rates used to determine kinetic parameters of R735Q AspH for **13**; (**c**) determination of the R735Q AspH v_{max}^{app} and K_{m}^{app} values for **13** (*i.e.*, 21 ± 2.0 nM·s^{-1} and 300 ± 79 µM, respectively).

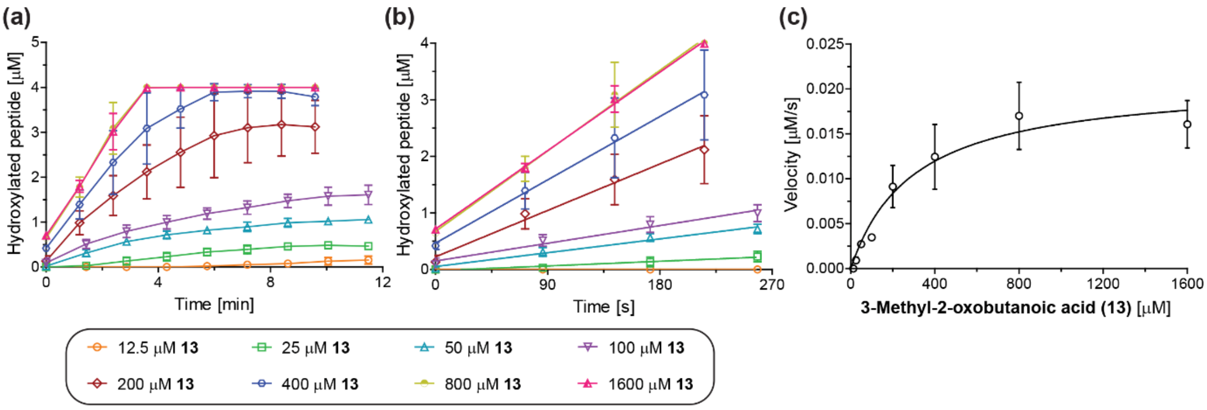

(**d**) Time course of the R735Q AspH-catalyzed oxidation of hFX-EGFD1$_{86\text{-}124}$-4S (Supporting Figure S1c) for the shown concentrations of (3*S*)-3-methyl-2-oxopentanoic acid (**15**); (**e**) oxidation rates used to determine kinetic parameters of R735Q AspH for **15**; (**f**) determination of the R735Q AspH v_{max}^{app} and K_{m}^{app} values for **15** (*i.e.*, 17 ± 3.3 nM·s^{-1} and 370 ± 110 µM, respectively); note that the data were fitted using non-linear regression to an equation which accounts for substrate inhibition ($Y = v_{max}^{app} \cdot X/(K_{m}^{app} + X \cdot (1+X/K_i))$), as increased concentrations of **15** appear to impair R735Q AspH catalysis.

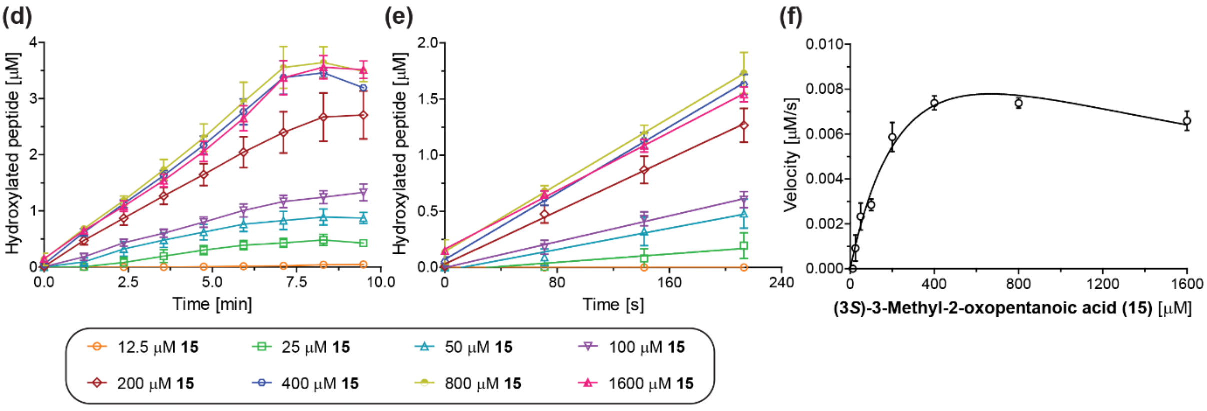

(**g**) Time course of the R735Q AspH-catalyzed oxidation of hFX-EGFD1$_{86\text{-}124}$-4S (Supporting Figure S1c) for the shown concentrations of 4-methyl-2-oxopentanoic acid (**16**); (**h**) oxidation rates used to determine kinetic parameters of R735Q AspH for **16**; (**i**) determination of the R735Q AspH v_{max}^{app} and K_m^{app} values for **16** (*i.e.*, 5.3 ± 1.5 nM·s^{-1} and 150 ± 71 μM, respectively); note that the data were fitted using non-linear regression to an equation which accounts for substrate inhibition (Y = v_{max}^{app}•X/(K_m^{app}+X•(1+X/K_i))), as increased concentrations of **16** appear to impair R735Q AspH catalysis.

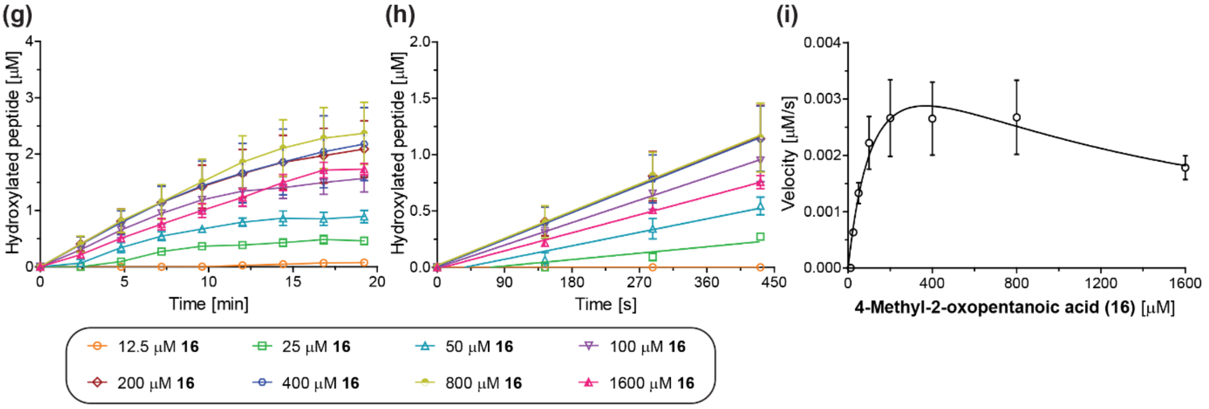

(**j**) Time course of the R735Q AspH-catalyzed oxidation of hFX-EGFD1$_{86\text{-}124}$-4S (Supporting Figure S1c) for the shown concentrations of 3-cyclohexyl-2-oxopropanoic acid (**18**); (**k**) oxidation rates used to determine kinetic parameters of R735Q AspH for **18**; (**l**) determination of the R735Q AspH v_{max}^{app} and K_m^{app} values for **18** (*i.e.*, 2.5 ± 0.8 nM·s^{-1} and 270 ± 150 μM, respectively); note that the data were fitted using non-linear regression to an equation which accounts for substrate inhibition (Y = v_{max}^{app}•X/(K_m^{app}+X•(1+X/K_i))), as increased concentrations of **18** appear to impair R735Q AspH catalysis.

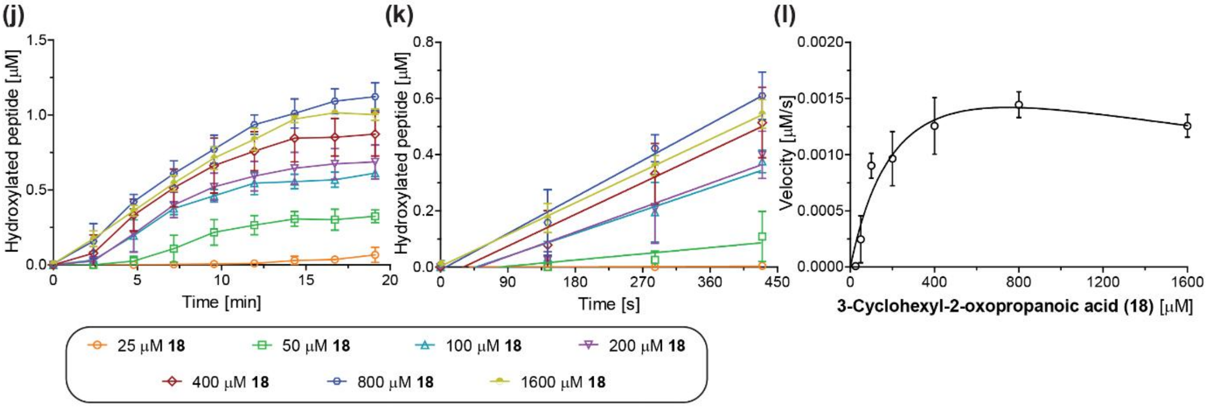

(**m**) Time course of the R735Q AspH-catalyzed oxidation of hFX-EGFD1$_{86\text{-}124}$-4S (Supporting Figure S1c) for the shown concentrations of 2-oxo-4-phenylbutanoic acid (**20**); (**n**) oxidation rates used to determine kinetic parameters of R735Q AspH for **20**; (**o**) determination of the R735Q AspH v_{max}^{app} and K_m^{app} values for **20** (*i.e.*, 6.7 ± 0.4 nM·s^{-1} and 87 ± 15 μM, respectively).

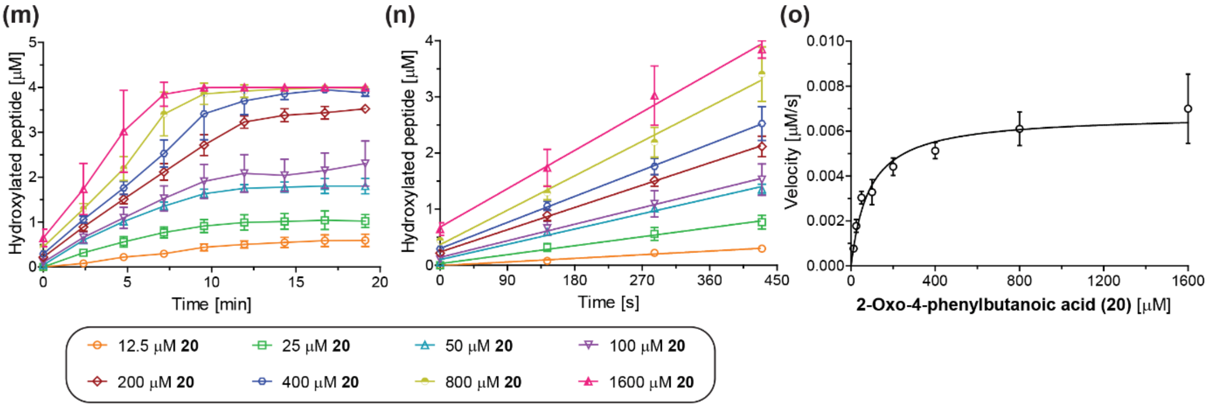

(**p**) Time course of the R735Q AspH-catalyzed oxidation of hFX-EGFD1$_{86\text{-}124}$-4S (Supporting Figure S1c) for the shown concentrations of 2-cyclohexyl-2-oxoacetic acid (**53**); (**q**) oxidation rates used to determine kinetic parameters of R735Q AspH for **53**; (**r**) determination of the R735Q AspH v_{max}^{app} and K_m^{app} values for **53** (*i.e.*, 8.9 ± 4.5 nM·s^{-1} and 700 ± 470 μM, respectively); note that the data were fitted using non-linear regression to an equation which accounts for substrate inhibition ($Y = v_{max}^{app} \cdot X/(K_m^{app} + X \cdot (1+X/K_i))$), as increased concentrations of **53** appear to impair R735Q AspH catalysis.

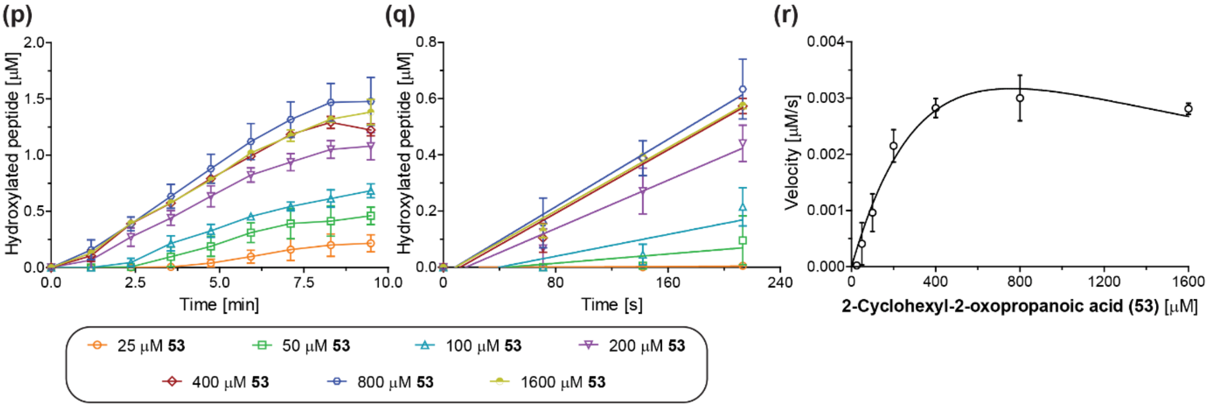

(**s**) Time course of the R735Q AspH-catalyzed oxidation of hFX-EGFD1$_{86\text{-}124}$-4S (Supporting Figure S1c) for the shown concentrations of 2-oxopentanoic acid (**54**); (**t**) oxidation rates used to determine kinetic parameters of R735Q AspH for **54**; (**u**) determination of the R735Q AspH $v_{\text{max}}^{\text{app}}$ and $K_{\text{m}}^{\text{app}}$ values for **54** (*i.e.*, 7.0 ± 0.1 nM·s^{-1} and 76 ± 8 μM, respectively).

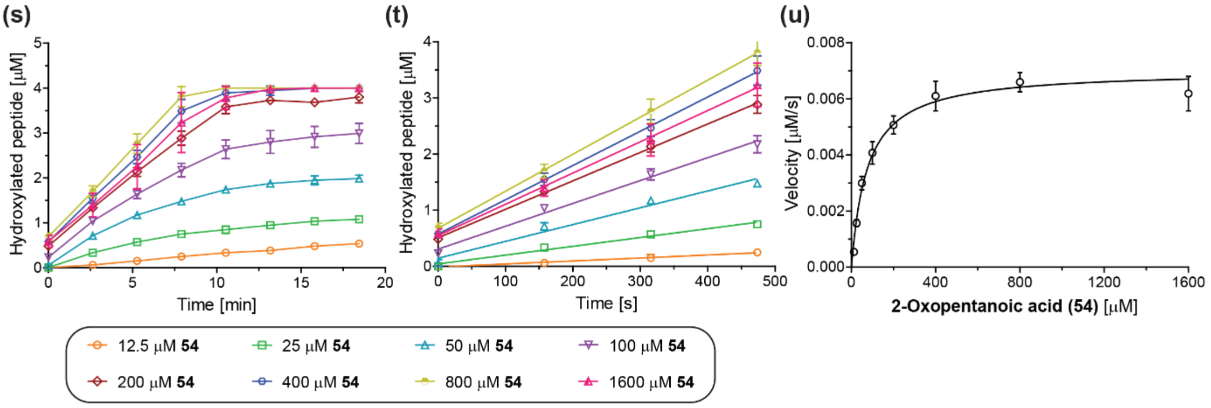

Supporting Figure S20. Rates and kinetic parameters for the reaction of R735W AspH with hydrophobic 2-oxoacids (continues on the 3 following pages). Maximum velocities (v_{max}^{app}) and apparent Michaelis constants (K_m^{app}) of isolated recombinant R735W AspH were determined for 3-methyl-2-oxobutanoic acid (**13**), (3*S*)-3-methyl-2-oxopentanoic acid (**15**), 4-methyl-2-oxopentanoic acid (**16**), 3-cyclohexyl-2-oxopropanoic acid (**18**), 2-oxo-4-phenylbutanoic acid (**20**), 2-cyclohexyl-2-oxoacetic acid (**53**), and 2-oxopentanoic acid (**54**), monitoring the R735W AspH-catalyzed hydroxylation of hFX-EGFD1$_{86\text{-}124}$-4S (Supporting Figure S1c) by SPE-MS as described (2, 9). Conditions: R735W His$_6$-AspH$_{315\text{-}758}$ (0.1 μM), hFX-EGFD1$_{86\text{-}124}$-4S (4.0 μM), L-ascorbic acid (400 μM), $(NH_4)_2Fe(SO_4)_2 \cdot 6H_2O$ (200 μM), and the shown 2-oxoacid concentrations in buffer (25 mM MES, pH 6.0, 20 °C). Measurement times were normalized to the first sample injection analyzed after the addition of R735W AspH to the Substrate Mixture (t = 0 s), by which time low levels of substrate oxidation were manifest. Note, the differences in y-intercept values for different conditions reflect differences in the extent of reaction during the delay between initiation of reaction by AspH addition and the first MS analysis. Data are means of independent triplicates (n = 3; mean ± SD).

(**a**) Time course of the R735W AspH-catalyzed oxidation of hFX-EGFD1$_{86\text{-}124}$-4S (Supporting Figure S1c) for the shown concentrations of 3-methyl-2-oxobutanoic acid (**13**); (**b**) oxidation rates used to determine kinetic parameters of R735W AspH for **13**; (**c**) determination of the R735W AspH v_{max}^{app} and K_m^{app} values for **13** (*i.e.*, 10.9 ± 0.7 nM·s^{-1} and 240 ± 42 μM, respectively).

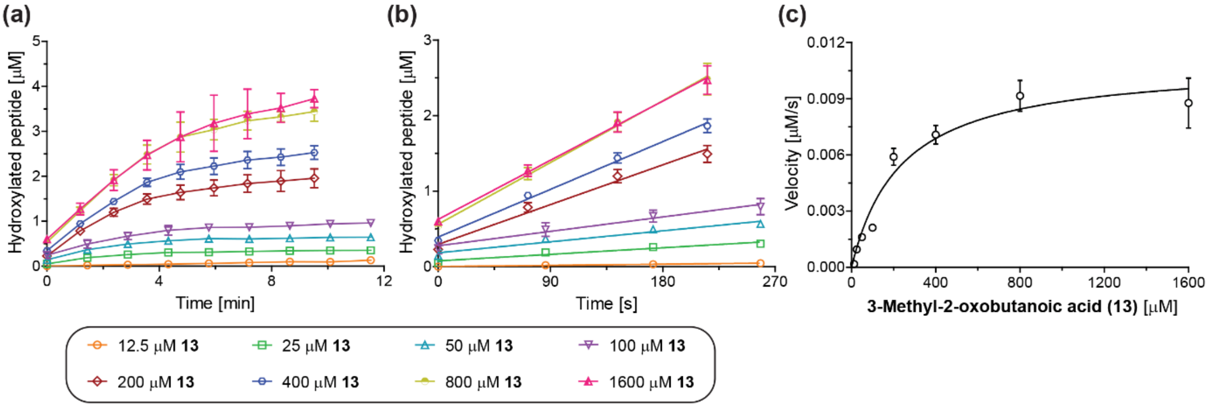

(**d**) Time course of the R735W AspH-catalyzed oxidation of hFX-EGFD1$_{86\text{-}124}$-4S (Supporting Figure S1c) for the shown concentrations of (3*S*)-3-methyl-2-oxopentanoic acid (**15**); (**e**) oxidation rates used to determine kinetic parameters of R735W AspH for **15**; (**f**) determination of the R735W AspH v_{max}^{app} and K_m^{app} values for **15** (*i.e.*, 10.4 ± 0.5 nM·s^{-1} and 250 ± 37 μM, respectively).

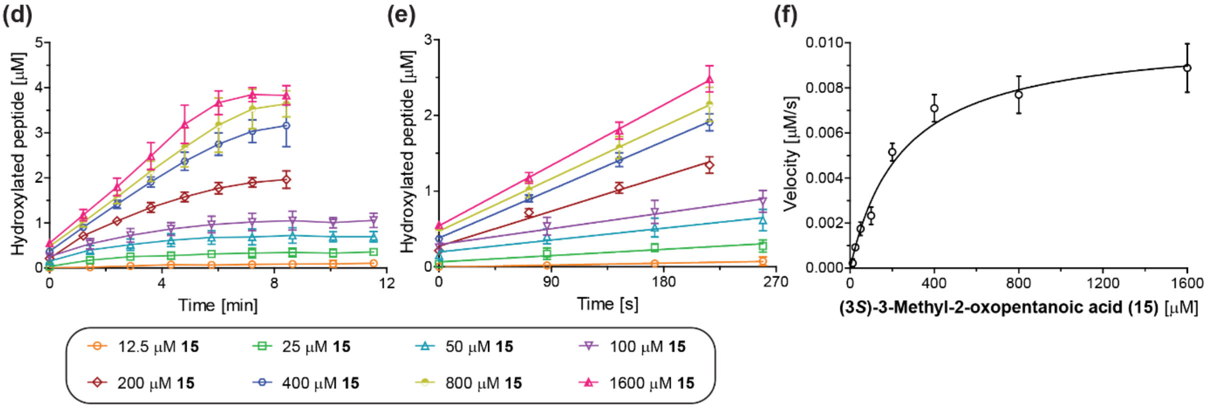

(**g**) Time course of the R735W AspH-catalyzed oxidation of hFX-EGFD1$_{86\text{-}124}$-4S (Supporting Figure S1c) for the shown concentrations of 4-methyl-2-oxopentanoic acid (**16**); (**h**) oxidation rates used to determine kinetic parameters of R735W AspH for **16**; (**i**) determination of the R735W AspH v_{max}^{app} and K_{m}^{app} values for **16** (*i.e.*, 8.5 ± 2.1 nM·s^{-1} and 240 ± 96 μM, respectively); note that the data were fitted using non-linear regression to an equation which accounts for substrate inhibition (Y = v_{max}^{app}•X/(K_{m}^{app}+X•(1+X/K_i))), as increased concentrations of **16** appear to impair R735W AspH catalysis.

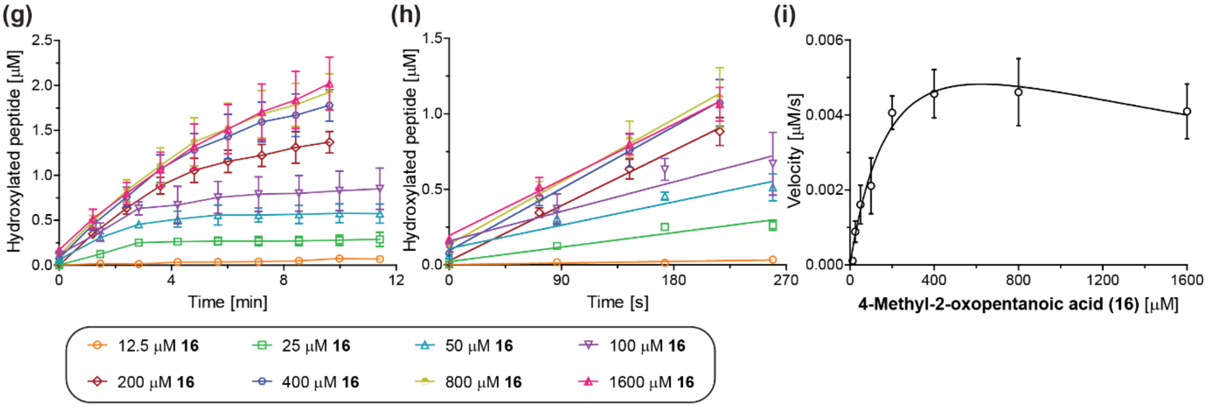

(**j**) Time course of the R735W AspH-catalyzed oxidation of hFX-EGFD1$_{86\text{-}124}$-4S (Supporting Figure S1c) for the shown concentrations of 3-cyclohexyl-2-oxopropanoic acid (**18**); (**k**) oxidation rates used to determine kinetic parameters of R735W AspH for **18**; (**l**) determination of the R735W AspH v_{max}^{app} and K_{m}^{app} values for **18** (*i.e.*, 2.0 ± 0.2 nM·s^{-1} and 210 ± 46 μM, respectively).

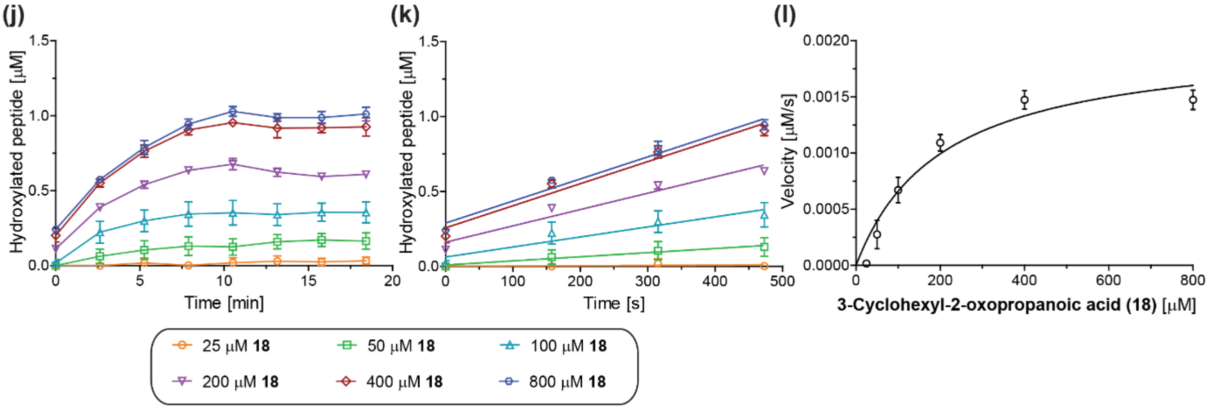

(**m**) Time course of the R735W AspH-catalyzed oxidation of hFX-EGFD1$_{86\text{-}124}$-4S (Supporting Figure S1c) for the shown concentrations of 2-oxo-4-phenylbutanoic acid (**20**); (**n**) oxidation rates used to determine kinetic parameters of R735W AspH for **20**; (**o**) determination of the R735W AspH v_{max}^{app} and K_{m}^{app} values for **20** (*i.e.*, 4.8 ± 0.6 nM·s^{-1} and 390 ± 120 µM, respectively).

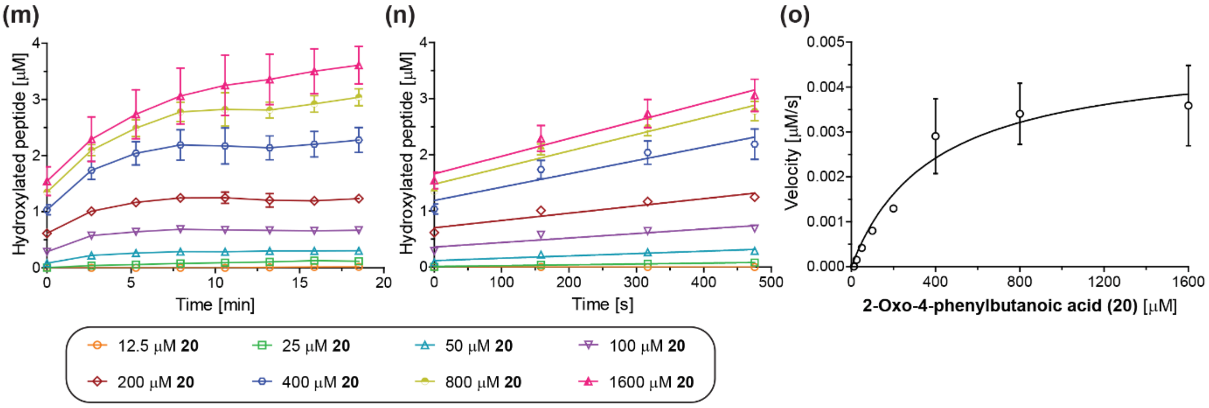

(**p**) Time course of the R735W AspH-catalyzed oxidation of hFX-EGFD1$_{86\text{-}124}$-4S (Supporting Figure S1c) for the shown concentrations of 2-cyclohexyl-2-oxoacetic acid (**53**); (**q**) oxidation rates used to determine kinetic parameters of R735W AspH for **53**; (**r**) determination of the R735W AspH v_{max}^{app} and K_{m}^{app} values for **53** (*i.e.*, 8.9 ± 0.5 nM·s^{-1} and 150 ± 27 µM, respectively).

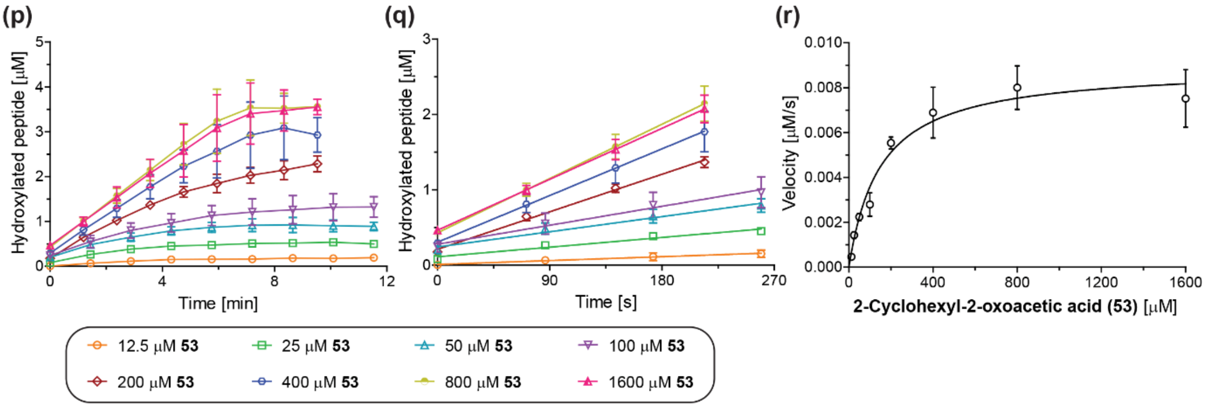

(**s**) Time course of the R735W AspH-catalyzed oxidation of hFX-EGFD1$_{86\text{-}124}$-4S (Supporting Figure S1c) for the shown concentrations of 2-oxopentanoic acid (**54**); (**t**) oxidation rates used to determine kinetic parameters of R735W AspH for **54**; (**u**) determination of the R735W AspH v_{max}^{app} and K_m^{app} values for **54** (*i.e.*, 3.6 ± 0.1 nM·s^{-1} and 82 ± 9 μM, respectively).

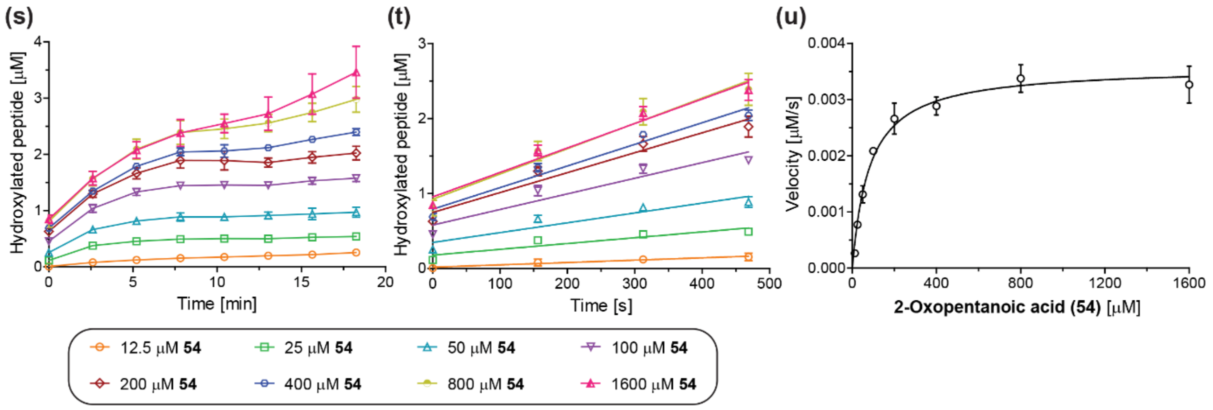

Supporting Figure S21. Views from a crystal structure of R688Q AspH complexed with 3-methyl-2OG (1), Mn, and a synthetic EGFD substrate peptide (R688Q AspH:1:Mn:hFX-EGFD1$_{86\text{-}124}$-4S; PDB ID: 8RE8). Colors: grey: R688Q His$_6$-AspH$_{315\text{-}758}$; salmon: carbon-backbone of 3-methyl-2OG (**1**); lavendar: Mn; yellow: carbon-backbone of hFX-EGFD1$_{86\text{-}124}$-4S (Supporting Figure S1c); red: oxygen; blue: nitrogen; gold: sulfur. (**a**) Overview of the R688Q AspH:**1**:Mn:hFX-EGFD1$_{86\text{-}124}$-4S crystal structure. (**b**) Representative Polder omit electron density map contoured to 3σ around the hFX-EGFD1$_{86\text{-}124}$-4S peptide from the R688Q AspH:**1**:Mn:hFX-EGFD1$_{86\text{-}124}$-4S structure reveals electron density for hFX-EGFD1$_{86\text{-}124}$-4S residues K100$_{hFX}$ to S112$_{hFX}$, including for the disulfide bridged (C101$_{hFX}$ and C110$_{hFX}$) ten-membered non-canonical EGFD macrocycle. The electron density observed for substrate residues L113$_{hFX}$ and E114$_{hFX}$ is relatively weak, and their modelled conformations should therefore be regarded as tentative.

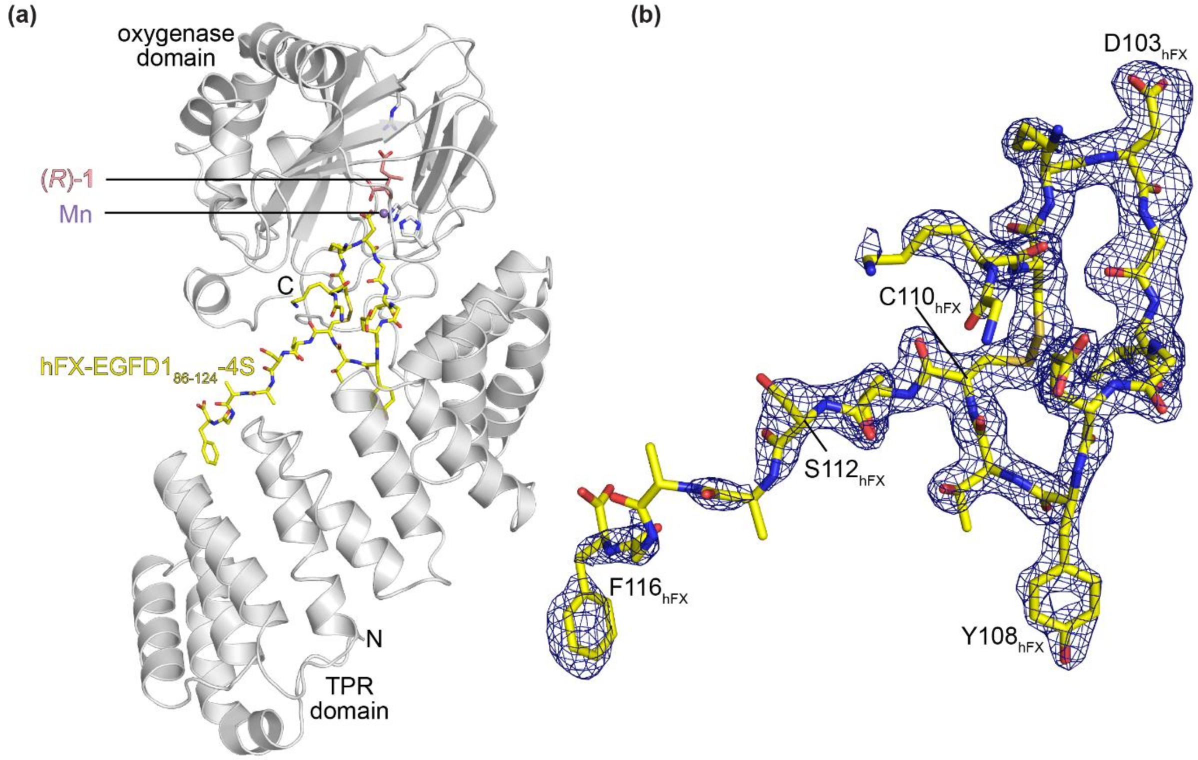

Supporting Figure S22. R688Q AspH and wt AspH adopt similar folds in complex with Mn, 3-methyl-2OG, and the hFX-EGFD1$_{86\text{-}124}$-4S substrate. Colors: grey: R688Q His$_6$-AspH$_{315\text{-}758}$; salmon: carbon-backbone of 3-methyl-2OG (**1**); lavendar: Mn; yellow: carbon-backbone of hFX-EGFD1$_{86\text{-}124}$-4S (Supporting Figure S1c); red: oxygen; blue: nitrogen; gold: sulfur.

(**a** and **b**) Superimposition of a view from the R688Q AspH:**1**:Mn:hFX-EGFD1$_{86\text{-}124}$-4S structure (Supporting Figure S21; PDB ID: 8RE8) with one from the reported wt AspH:**1**:Mn:hFX-EGFD1$_{86\text{-}124}$-4S structure (wt AspH: light pink, Mn: purple, carbon-backbone of 2OG: olive, carbon-backbone of hFX-EGFD1$_{86\text{-}124}$-4S: blue; PDB ID: 6YYX (10)) reveals similar: (**a**) AspH conformations (Cα RMSD ~ 0.18 Å) and (**b**) hFX-EGFD1$_{86\text{-}124}$-4S conformations (Cα RMSD ~ 0.20 Å), in particular of the residues forming the disulfide bridged (C101$_{hFX}$ and C110$_{hFX}$) ten-membered non-canonical EGFD macrocycle. As in the reported wt AspH:**1**:Mn:hFX-EGFD1$_{86\text{-}124}$-4S complex structure (PDB ID: 6YYX (10)), the D103$_{hFX}$ side chain carboxylate of hFX-EGFD1$_{86\text{-}124}$-4S adopts a single conformation in which it directly binds to Mn.

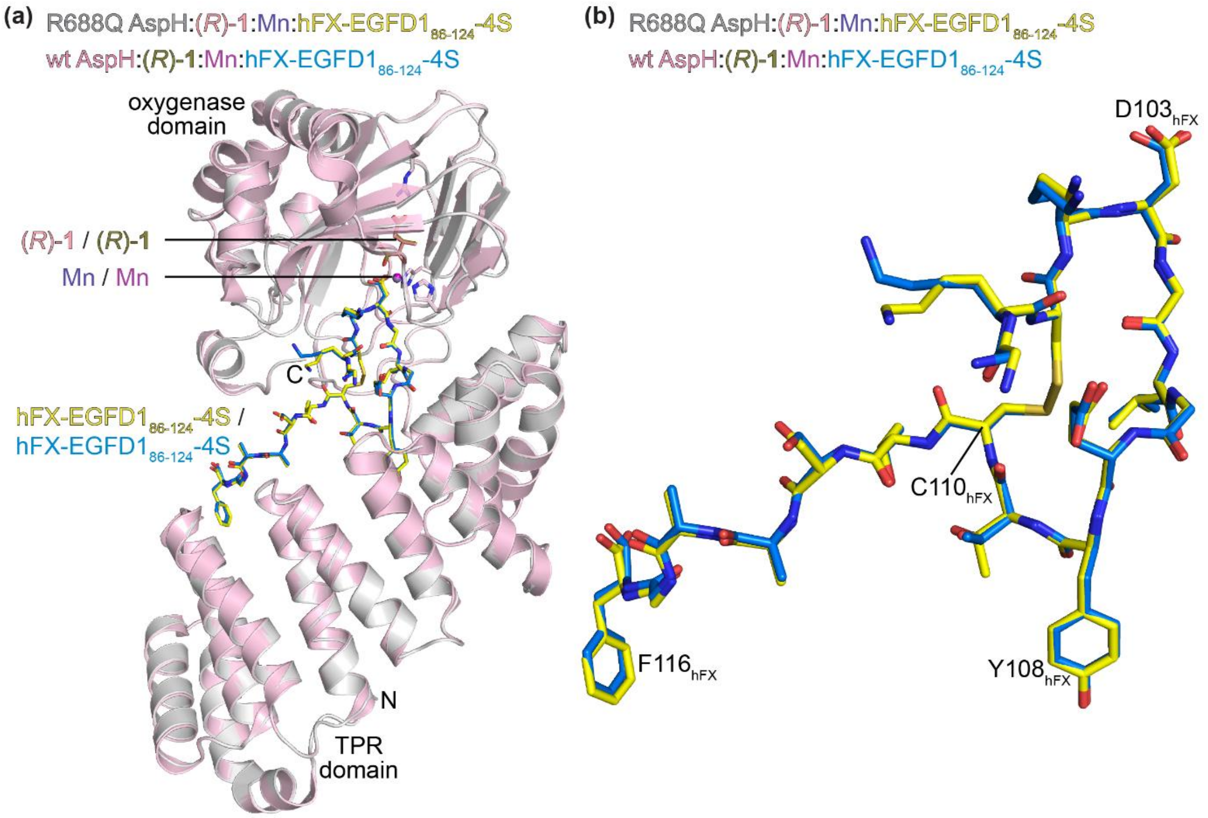

Supporting Figure S23. R688Q AspH in complex with Mn, 3-methyl-2OG, and the hFX-EGFD1$_{86\text{-}124}$-4S substrate adopts a similar fold as wt AspH in complex with Mn, 2OG, and the hFX-EGFD1$_{86\text{-}124}$-4S substrate. Colors: grey: R688Q His$_6$-AspH$_{315\text{-}758}$; salmon: carbon-backbone of 3-methyl-2OG (**1**); lavendar: Mn; yellow: carbon-backbone of hFX-EGFD1$_{86\text{-}124}$-4S (Supporting Figure S1c); red: oxygen; blue: nitrogen; gold: sulfur.

(**a** and **b**) Superimposition of a view from the R688Q AspH:**1**:Mn:hFX-EGFD1$_{86\text{-}124}$-4S structure (Supporting Figure S21; PDB ID: 8RE8) with one from the improved resolution wt AspH:2OG:Mn:hFX-EGFD1$_{86\text{-}124}$-4S structure (wt AspH: brown, Mn: pink, carbon-backbone of 2OG: orange, carbon-backbone of hFX-EGFD1$_{86\text{-}124}$-4S: green; PDB ID: 8RE9; Supporting Figure S10) reveals similar: (**a**) AspH conformations (Cα RMSD ~ 0.18 Å) and (**b**) hFX-EGFD1$_{86\text{-}124}$-4S conformations (Cα RMSD ~ 0.16 Å), in particular of the residues forming the disulfide bridged (C101$_{hFX}$ and C110$_{hFX}$) ten-membered non-canonical EGFD macrocycle. Notably, the D103$_{hFX}$ side-chain carboxylate of hFX-EGFD1$_{86\text{-}124}$-4S was modelled with one conformation in the R688Q AspH:**1**:Mn:hFX-EGFD1$_{86\text{-}124}$-4S structure, but with two conformations in the improved resolution wt AspH:2OG:Mn:hFX-EGFD1$_{86\text{-}124}$-4S structure.

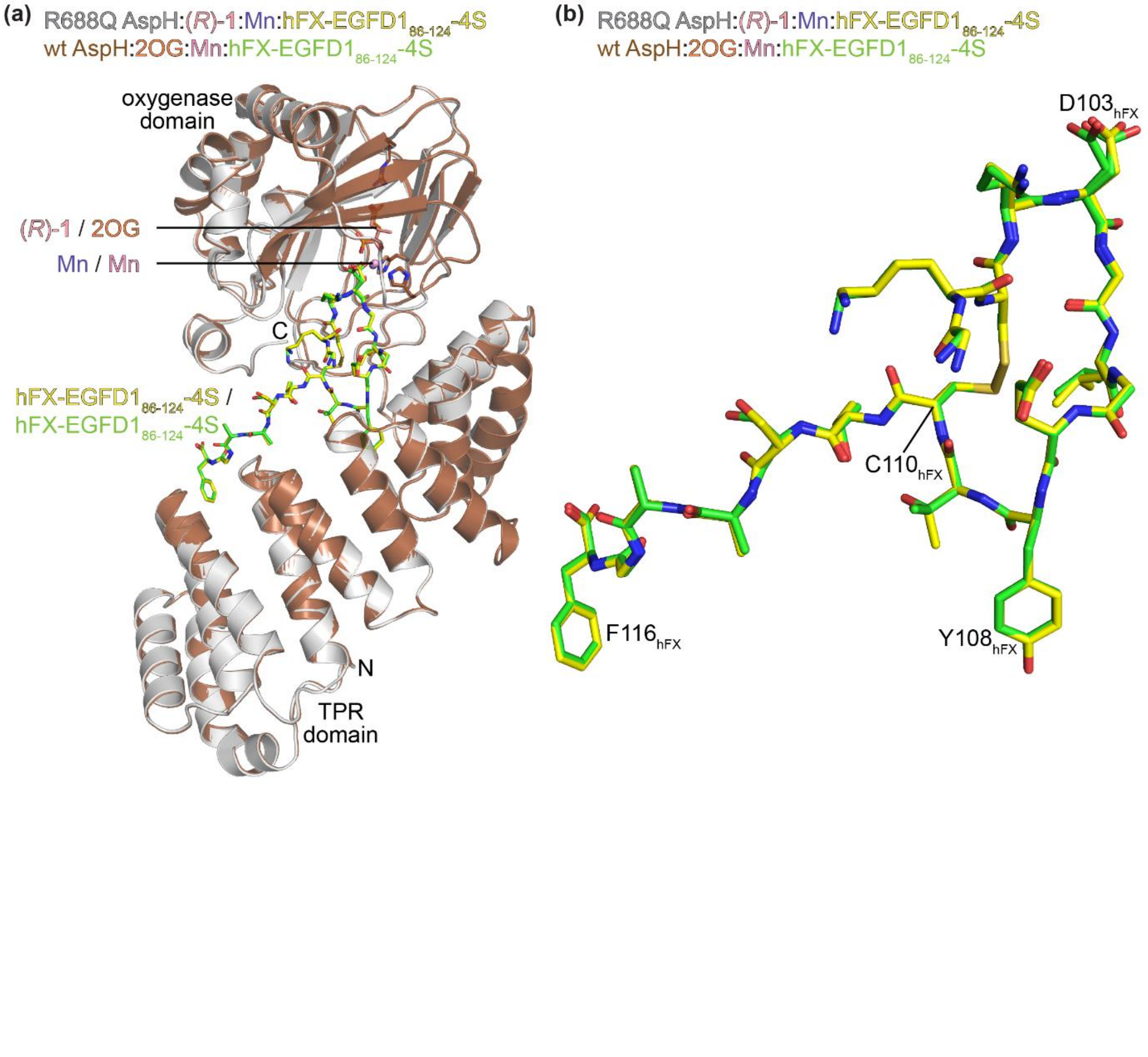

Supporting Figure S24. Analysis of electron density maps supports the predominant presence of the (*R*)-enantiomer of 3-methyl-2OG (1) in the R688Q AspH:1:Mn:hFX-EGFD1$_{86\text{-}124}$-4S complex structure (PDB ID: 8RE8). Colors: grey: R688Q His$_6$-AspH$_{315\text{-}758}$; salmon: carbon-backbone of 3-methyl-2OG (**1**); lavendar: Mn; yellow: carbon-backbone of hFX-EGFD1$_{86\text{-}124}$-4S (Supporting Figure S1c); red: oxygen; blue: nitrogen; gold: sulfur. w: water.

(**a**) 3-Methyl-2OG (**1**) adopts a conformation in the R688Q AspH:**1**:Mn:hFX-EGFD1$_{86\text{-}124}$-4S structure which is apparently identical to that observed for **1** in the reported wt AspH:**1**:Mn:hFX-EGFD1$_{86\text{-}124}$-4S (PDB ID: 6YYX (10)) structure and to that observed for 2OG in the improved resolution wt AspH:2OG:Mn:hFX-EGFD1$_{86\text{-}124}$-4S (PDB ID: 8RE9; Supporting Figure S10) structure (Figure 5a-c). The C-1 carboxylate of **1** coordinates Mn *trans* to H679 (2.3 Å) and is positioned to interact with H690 (2.8 Å) and Q688 (2.6 and 2.7 Å), and the side chain carboxylate of D103hFX (3.0 Å) of hFX-EGFD1$_{86\text{-}124}$-4S. The C-2 ketone oxygen atom of **1** coordinates Mn (2.2 Å) *trans* to the complexed water molecule (2.2 Å). The C-5 carboxylate of **1** is positioned to form a salt bridge with the guanidinium group of R735 (2.7 Å) and to interact with the side chain of S668 (2.4 Å). The methyl group of **1** adopts a conformation in which it can engage in hydrophobic interactions with the proximate hydrophobic side chains of W625, M670, V676, and V727. The imidazole rings of both H679 and H725 coordinate to Mn (2.3 and 2.2 Å, respectively); the side chain carboxylate of the D103$_{hFX}$ substrate residue coordinates to Mn *trans* to H725 (3.0 Å). (**b** and **c**) 2mF$_o$-DF$_c$ (blue, 1σ contour level) and mF$_o$-DF$_c$ (green, +3σ contour level and red -3σ contour level) electron density map for: (**b**) (*R*)-**1** and (**c**) (*S*)-**1** modelled in the R688Q AspH:**1**:Mn:hFX-EGFD1$_{86\text{-}124}$-4S complex structure. The results reveal that modelling of **1** as the (*R*)-enantiomer results in a conformation of **1** which fits the electron density observed around the C-2 to C-5 carbon atoms; analysis of difference maps does not show positive or negative densities except for the C-1 carboxylate group (**b**). By contrast, modelling of **1** as the (*S*)-enantiomer results in a conformation which does not fit the observed electron density as well as the (*R*)-enantiomer: Negative densities are observed around the C-3 carbon atoms, as well as around the C-1 and C-5 carboxylate groups of the (*S*)-enantiomer in the difference maps, while positive densities are observed around the C-4 carbon atom. Thus, the combined electron density analyses indicate that the (*R*)-enantiomer of **1** is predominantly (at least) present in the R688Q AspH:**1**:Mn:hFX-EGFD1$_{86\text{-}124}$-4S complex structure.

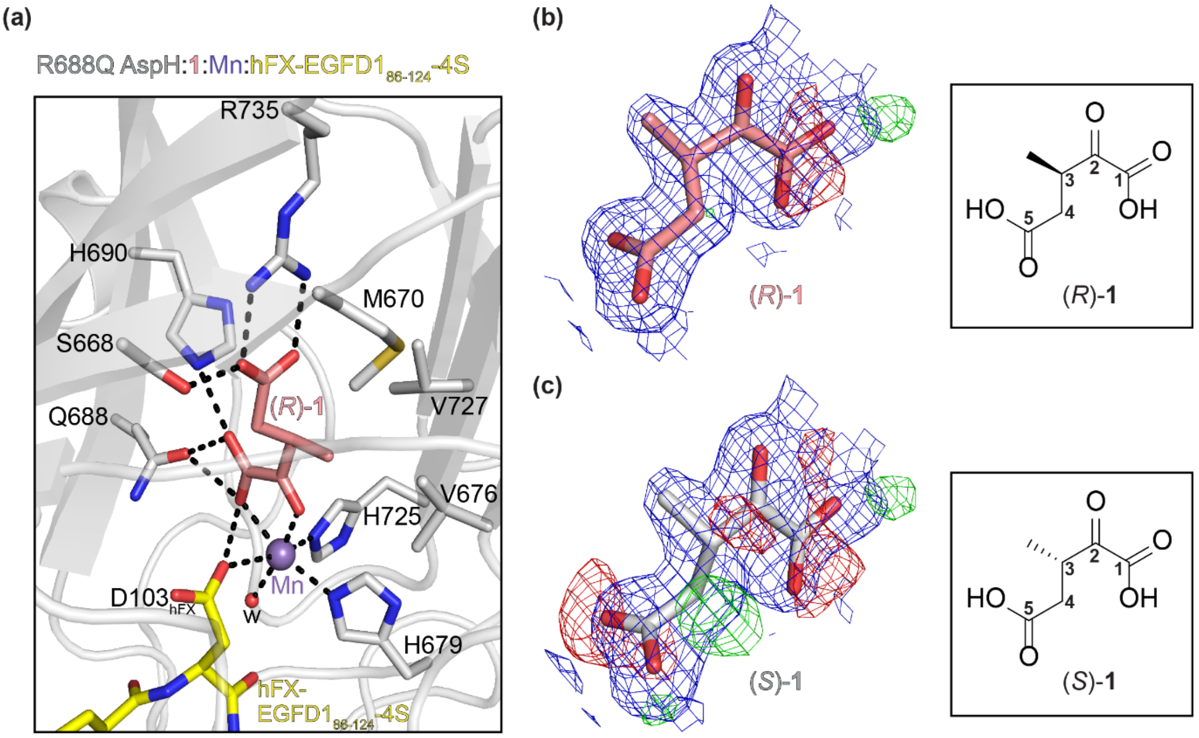

Supporting Figure S25. Views from a crystal structure of R735Q AspH complexed with 2OS (12), Mn, and a synthetic EGFD substrate peptide (R735Q AspH:12:Mn:hFX-EGFD1$_{86\text{-}124}$-4S; PDB ID: 8RE5). Colors: grey: R735Q His$_6$-AspH$_{315\text{-}758}$; lemon/rosy brown: carbon-backbone of 2-oxosuberate (2OS; **12**); lavendar: Mn; yellow: carbon-backbone of hFX-EGFD1$_{86\text{-}124}$-4S (Supporting Figure S1c); red: oxygen; blue: nitrogen; gold: sulfur.

(**a**) Overview of the R735Q AspH:**12**:Mn:hFX-EGFD1$_{86\text{-}124}$-4S crystal structure. Note that a second 2OS molecule is bound to the surface of the AspH oxygenase domain. (**b**) Representative Polder omit electron density map contoured to 3σ around hFX-EGFD1$_{86\text{-}124}$-4S from the R735Q AspH:**12**:Mn:hFX-EGFD1$_{86\text{-}124}$-4S structure reveals electron density for hFX-EGFD1$_{86\text{-}124}$-4S residues G99$_{hFX}$ to F116$_{hFX}$, including for the disulfide bridged (C101$_{hFX}$ and C110$_{hFX}$) ten-membered non-canonical EGFD macrocycle. (**c**) Representative Polder omit electron density map contoured to 3σ around 2OS bound to the surface of R735Q AspH. 2OS is positioned to interact with H671 via its C-8 carboxylate group (2.8 Å) and binds to a hydrophobic pocket formed by the side chains of *e.g.*, M605, F612, and I669. The relevance of this alternative 2OS coordination mode for AspH catalysis is unclear; it could be a crystallographic artefact considering the use of relatively high 2OS concentrations during crystallization or, at least in principle, it could be involved in shuttling the cosubstrate to the active site.

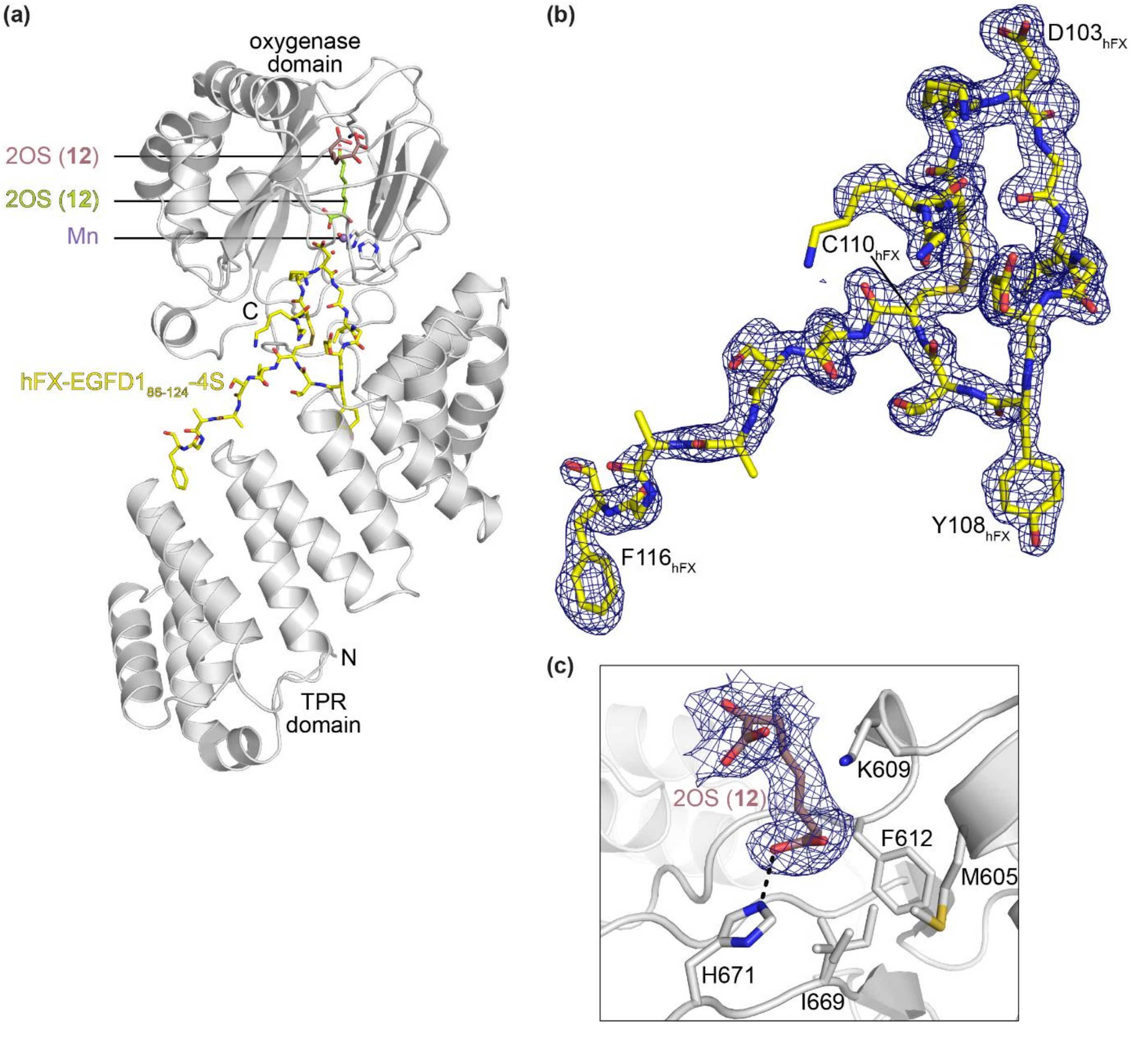

Supporting Figure S26. The R735Q AspH variant adopts a similar fold in complex with 2OS and 2OG. Colors: grey: R735Q His_6-$AspH_{315-758}$; lemon/rosy brown: carbon-backbone of 2-oxosuberate (2OS; **12**); lavendar: Mn; yellow: carbon-backbone of hFX-$EGFD1_{86-124}$-4S (Supporting Figure S1c); red: oxygen; blue: nitrogen; gold: sulfur.

Superimposition of a view from the R735Q AspH:**12**:Mn:hFX-$EGFD1_{86-124}$-4S structure (Supporting Figure S25; PDB ID: 8RE5) with one from the R735Q AspH:2OG:Mn:hFX-$EGFD1_{86-124}$-4S structure (wt AspH: light pink, Mn: purple, carbon-backbone of 2OG: teal, carbon-backbone of hFX-$EGFD1_{86-124}$-4S: blue; PDB ID: 8RE6; Supporting Figure S8) reveals similar: (**a**) AspH conformations (Cα RMSD ~ 0.27 Å) and (**b**) hFX-$EGFD1_{86-124}$-4S conformations (Cα RMSD ~ 0.37 Å), in particular of the residues forming the disulfide bridged ($C101_{hFX}$ and $C110_{hFX}$) ten-membered non-canonical EGFD macrocycle. Notably, the $D103_{hFX}$ side chain carboxylate of hFX-$EGFD1_{86-124}$-4S adopts a single conformation in which it directly binds to Mn, as also observed in the R735Q AspH:2OG:Mn:hFX-$EGFD1_{86-124}$-4S structure.

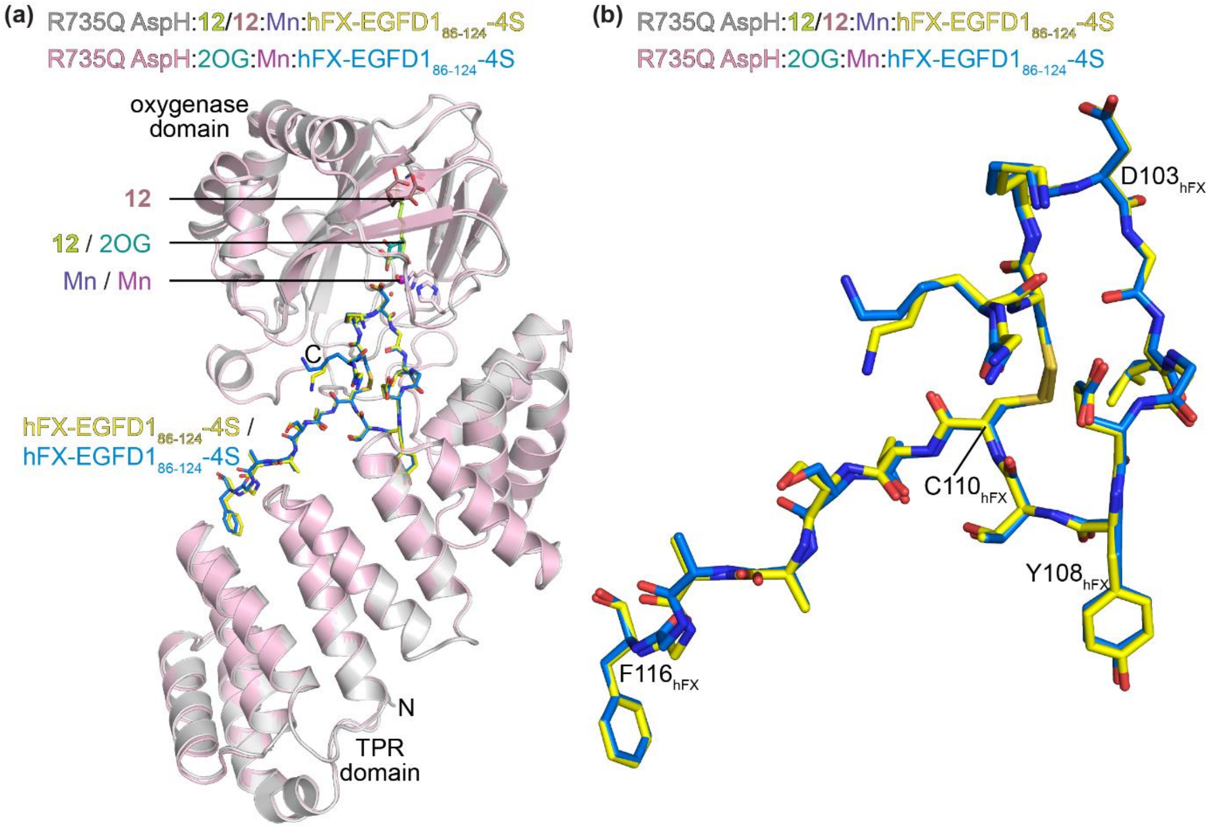

Supporting Figure S27. The R735Q AspH variant in complex with Mn, 2OS, and a substrate peptide adopts a similar fold as wt AspH in complex with Mn, 2OG, and a substrate peptide. Colors: grey: R735Q His$_6$-AspH$_{315-758}$; lemon/rosy brown: carbon-backbone of 2-oxosuberate (2OS; **12**); lavendar: Mn; yellow: carbon-backbone of hFX-EGFD1$_{86-124}$-4S (Supporting Figure S1c); red: oxygen; blue: nitrogen; gold: sulfur. Superimposition of a view from the R735Q AspH:**12**:Mn:hFX-EGFD1$_{86-124}$-4S structure (Supporting Figure S25; PDB ID: 8RE5) with one from the improved resolution wt AspH:2OG:Mn:hFX-EGFD1$_{86-124}$-4S structure (wt AspH: brown, Mn: pink, carbon-backbone of 2OG: orange, carbon-backbone of hFX-EGFD1$_{86-124}$-4S: green; PDB ID: 8RE9; Supporting Figure S10) reveals similar: (**a**) AspH conformations (Cα RMSD ~ 0.17 Å) and (**b**) hFX-EGFD1$_{86-124}$-4S conformations (Cα RMSD ~ 0.13 Å), in particular of the residues forming the disulfide bridged (C101$_{hFX}$ and C110$_{hFX}$) ten-membered non-canonical EGFD macrocycle. Notably, the D103$_{hFX}$ side-chain carboxylate of hFX-EGFD1$_{86-124}$-4S adopts one conformation in the R735Q AspH:**12**:Mn:hFX-EGFD1$_{86-124}$-4S structure, but two conformations in the improved resolution wt AspH:2OG:Mn:hFX-EGFD1$_{86-124}$-4S structure.

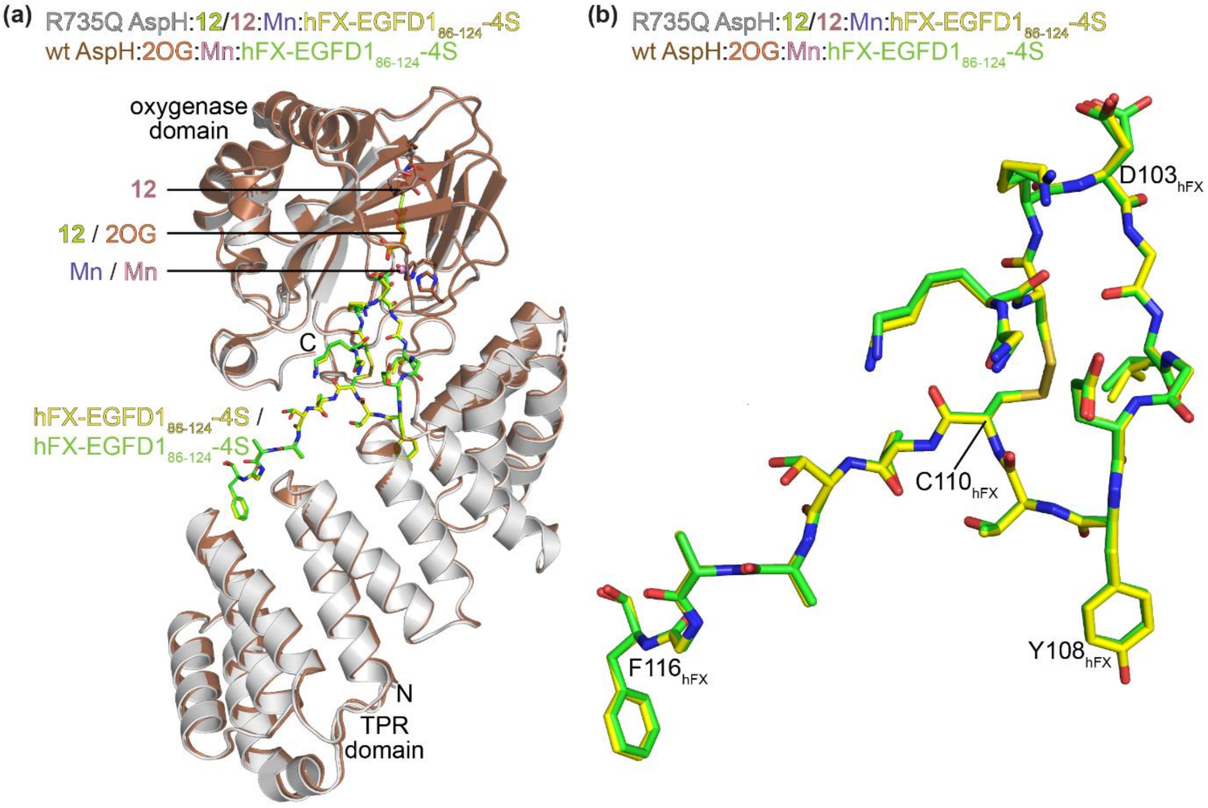

2. Supporting tables

Supporting Table S1. Data collection and refinement statistics for the wt, R735Q, and R735W AspH:Mn:2OG:hFX-EGFD1$_{86-124}$-4S complexes.

**Datasets**	**wt AspH:Mn:2OG:hFX-EGFD1$_{86-124}$-4S** (PDB ID: 8RE9)	**R735Q AspH:Mn:2OG:hFX-EGFD1$_{86-124}$-4S** (PDB ID: 8RE6)	**R735W AspH:Mn:2OG:hFX-EGFD1$_{86-124}$-4S** (PDB ID: 8RE7)
**Data Collection (T in K)**	MX (100)	MX (100)	MX (100)
Beamline (Wavelength, Å)	DLS I03 (0.9763)	DLS I03 (0.9763)	DLS I03 (0.9763)
Detector	EIGER2 XE 16M	EIGER2 XE 16M	EIGER2 XE 16M
Data Processing	Xia2, DIALS	AIMLESS	Xia2, DIALS
Space group	$P\,2_1\,2_1\,2_1$	$P\,2_1\,2_1\,2_1$	$P\,2_1\,2_1\,2_1$
Cell dimensions:			
a,b,c (Å)	49.7, 86.2, 123.4	50.1, 90.9, 123.4	50.1, 91.0, 124.9
α, β, γ (°)	90, 90, 90	90, 90, 90	90, 90, 90
No. of molecules/ASU	1	1	1
No. reflections	46657 (2216)*	43909 (2916)*	42632 (2070)*
Resolution (Å)	49.68-1.84 (1.87-1.84)*	61.68-1.92 (1.97-1.92)*	51.50-1.95 (1.98-1.95)*
R_{merge}	0.092 (1.383)*	0.106 (1.659)*	0.069 (0.779)*
I/σI	19.5 (1.80)*	12.7 (1.30)*	21.6 (1.60)*
CC-half	1.000 (0.898)*	0.983 (0.761)*	0.917 (0.895)*
Completeness (%)	99.94 (99.61)*	100 (100)*	100 (98.1)*
Multiplicity	13.4 (12.9)*	13.5 (13.90)*	13.4 (12.12)*
Wilson B value (Å^2)	26.35	34.72	35.1
**Refinement**	PHENIX	PHENIX	PHENIX
R_{work}/R_{free+}	0.1623/0.1956	0.1940 /0.2157	0.2043/0.2419
No. atoms	4002	3888	3812
- Enzyme	3581	3544	3523
- ligand	53	11	11
- Water	368	333	278
Average B-factors	33.10	44.03	47.85
- Enzyme	32.11	43.91	47.74
- ligand	48.35	35.69	30.32
- Water	40.57	45.58	50.01
R.m.s deviations			
- Bond lengths (Å)	0.009	0.002	0.007
- Bond angles (°)	0.90	0.52	0.85

*Highest resolution shell in parentheses. DLS: Diamond Light Source; ASU: asymmetric unit.

Supporting Table S2. Effects of 2OG derivatives on catalysis by isolated recombinant human wt AspH and Traboulsi Syndrome-associated AspH variants (continues on the following 4 pages). SPE-MS AspH assays were performed as reported (2, 9), using: wt $AspH_{315\text{-}758}$ (0.1 µM) or G434V His_6-$AspH_{315\text{-}758}$ (0.1 µM), incubated with hFX-$EGFD1_{86\text{-}124}$-4S (1, 2) (4.0 µM), L-ascorbic acid (100 µM), $(NH_4)_2Fe(SO_4)_2{\cdot}6H_2O$ (20 µM), and a 2OG derivative (500 µM) in buffer (25 mM HEPES, pH 7.5, 50 mM NaCl) for 15 min at 20 ºC; or: R688Q His_6-$AspH_{315\text{-}758}$ (0.2 µM), incubated with hFX-$EGFD1_{86\text{-}124}$-4S (4.0 µM), L-ascorbic acid (400 µM), $(NH_4)_2Fe(SO_4)_2{\cdot}6H_2O$ (100 µM), and a 2OG derivative (50 µM) in buffer (25 mM MES, pH 6.0) for 30 min at 20 ºC; or: R735Q His_6-$AspH_{315\text{-}758}$ (0.2 µM) or R735W His_6-$AspH_{315\text{-}758}$ (0.2 µM), incubated with hFX-$EGFD1_{86\text{-}124}$-4S (4.0 µM), L-ascorbic acid (400 µM), $(NH_4)_2Fe(SO_4)_2{\cdot}6H_2O$ (100 µM), and a 2OG derivative (2000 µM) in buffer (25 mM MES, pH 6.0) for 3 h at 20 ºC. Note that the activity of R735W AspH with 2OG derivatives was also tested in another buffer (25 mM HEPES, pH 7.5, 50 mM NaCl) under otherwise identical conditions, because R735W AspH did not show activity with 2OG in MES buffer. All the tested 2OG derivatives did not efficiently sustain catalysis of R735W AspH in HEPES buffer, whereas at least 2OG derivative **8** sustained high levels of cosubstrate activity with R735W AspH under acidic conditions in MES buffer.
Note that the reactivity of wt AspH with some of the shown 2OG derivatives has been reported using a hFX-derived substrate other than hFX-$EGFD1_{86\text{-}124}$-4S (10).

[a]2OG derivative	wt AspH	G434V AspH	R688Q AspH	R735Q AspH	R735W AspH
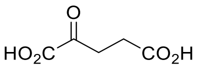 **2OG**	**>95%**	**>95%**	35% ± 3%	**62% ± 10%**	<1% (MES)<br><1% (HEPES)
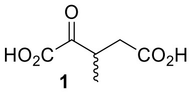 **1**	**94% ± 6%**	**90% ± 6%**	**73% ± 3%**	7% ± 2%	<1% (MES)<br><1% (HEPES)
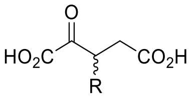 **2**: R = $CH_2CH_2CH_3$	15% ± 3%	<1%	<1%	<1%	<1% (MES)<br><1% (HEPES)
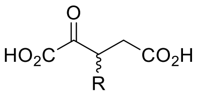 **21**: R = $CH_2CH_2CH_2CH_3$	<5%	<1%	<1%	<1%	<1% (MES)<br><1% (HEPES)
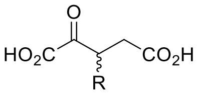 **22**: R = $CH_2CH_2C(CH_3)_3$	<1%	<1%	<1%	<1%	<1% (MES)<br><1% (HEPES)
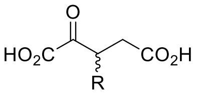 **23**: R = $CH_2CH_2CH_2Ph$	<1%	<1%	<1%	<1%	<1% (MES)<br><1% (HEPES)
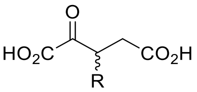 **24**: R = CH_2Ph	6% ± 2%	<1%	7% ± 1%	<1%	<1% (MES)<br><1% (HEPES)

[a]2OG derivative	wt AspH	G434V AspH	R688Q AspH	R735Q AspH	R735W AspH
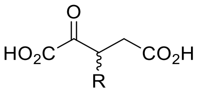 **25**: R = $CH_2(4\text{-}FC_6H_4)$	<1%	<1%	<1%	<1%	<1% (MES)<br><1% (HEPES)
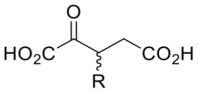 **26**: R = $CH_2(4\text{-}F_3COC_6H_4)$	<5%	<1%	<1%	<1%	<1% (MES)<br><1% (HEPES)
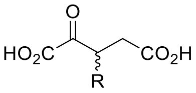 **27**: R = $CH_2(4\text{-}MeOC_6H_4)$	26% ± 4%	14% ± 1%	16% ± 4%	9% ± 2%	<1% (MES)<br><1% (HEPES)
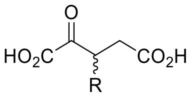 **28**: R = $CH_2(3,5\text{-}Me_2C_6H_4)$	<1%	<1%	<1%	<1%	<1% (MES)<br><1% (HEPES)
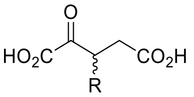 **29**: R = CH_2(9,9-dimethyl-9*H*-fluoren-2-yl)	<1%	<1%	<1%	<1%	<1% (MES)<br><1% (HEPES)
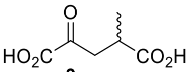 **3**	**44% ± 13%**	5% ± 5%	<1%	<1%	<1% (MES)<br><1% (HEPES)
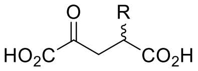 **30**: R = CH_2CH_3	<1%	<1%	<1%	<1%	<1% (MES)<br><1% (HEPES)
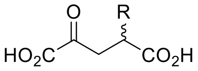 **4**: R = $CH_2CH_2CH_3$	<1%	<1%	<1%	<1%	<1% (MES)<br><1% (HEPES)
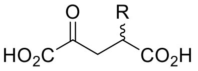 **31**: R = $CH_2CH_2CH_2CH_3$	<1%	<1%	<1%	<1%	<1% (MES)<br><1% (HEPES)
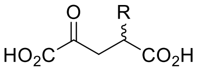 **32**: R = $CH_2CH(CH_3)_2$	<1%	<1%	<1%	<1%	<1% (MES)<br><1% (HEPES)
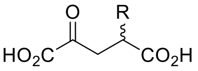 **33**: R = $CH_2CH_2C(CH_3)_3$	<1%	<1%	<1%	<1%	<1% (MES)<br><1% (HEPES)
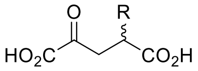 **34**: R = $CH_2CH_2CH_2Ph$	<1%	<1%	<1%	<1%	<1% (MES)<br><1% (HEPES)

[a]2OG derivative	wt AspH	G434V AspH	R688Q AspH	R735Q AspH	R735W AspH
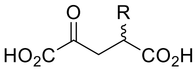 **35**: R = CH_2Ph	<1%	<1%	<1%	<1%	<1% (MES) <1% (HEPES)
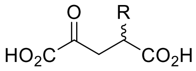 **36**: R = CH_2(2-naphthyl)	<1%	<1%	<1%	<1%	<1% (MES) <1% (HEPES)
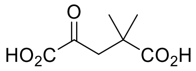 **37**	<1%	<1%	<1%	<1%	<1% (MES) <1% (HEPES)
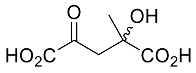 **38**	<1%	<1%	<1%	<1%	<1% (MES) <1% (HEPES)
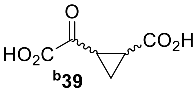 [b]**39**	**56% ± 13%**	**40% ± 8%**	<1%	<1%	<1% (MES) <1% (HEPES)
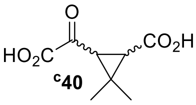 [c]**40**	<1%	<1%	<1%	<1%	<1% (MES) <1% (HEPES)
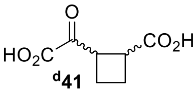 [d]**41**	**82% ± 6%**	14% ± 5%	<1%	<1%	<1% (MES) <1% (HEPES)
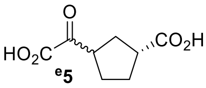 [e]**5**	**49% ± 6%**	9% ± 6%	<1%	**48% ± 3%**	<1% (MES) <1% (HEPES)
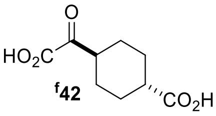 [f]**42**	<5%	7% ± 2%	<1%	**70% ± 8%**	<1% (MES) <1% (HEPES)
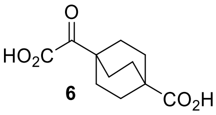 **6**	<1%	<1%	<1%	**49% ± 5%**	<1% (MES) <1% (HEPES)
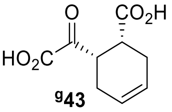 [g]**43**	12% ± 1%	<1%	<1%	<1%	<1% (MES) <1% (HEPES)
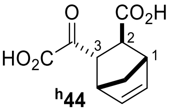 [h]**44**	<1%	<1%	<1%	<1%	<1% (MES) <1% (HEPES)

[a]2OG derivative	wt AspH	G434V AspH	R688Q AspH	R735Q AspH	R735W AspH
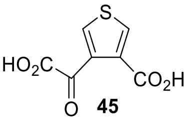 45	<1%	<1%	<1%	<1%	<1% (MES)<br><1% (HEPES)
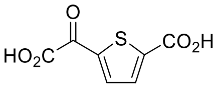 46	<1%	<1%	<1%	<1%	<1% (MES)<br><1% (HEPES)
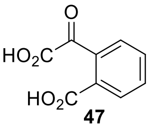 47	11% ± 10%	14% ± 10%	<1%	<1%	<1% (MES)<br><1% (HEPES)
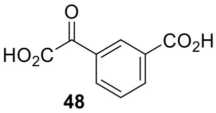 48	<5%	<1%	<1%	**39% ± 4%**	13% ± 3% (MES)<br><1% (HEPES)
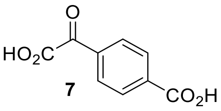 7	31% ± 8%	8% ± 5%	<1%	**90% ± 13%**	<1% (MES)<br><1% (HEPES)
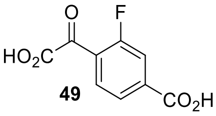 49	26% ± 5%	25% ± 10%	<1%	**>95%**	8% ± 4% (MES)<br><1% (HEPES)
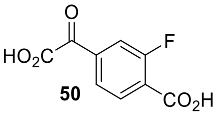 50	11% ± 2%	<5%	<1%	8% ± 2%	<1% (MES)<br><1% (HEPES)
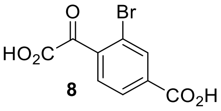 8	**61% ± 13%**	16% ± 6%	<1%	**>95%**	**83% ± 20%** (MES)<br><5% (HEPES)
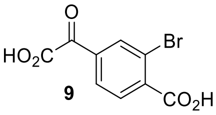 9	<1%	<1%	<1%	<1%	<1% (MES)<br><1% (HEPES)
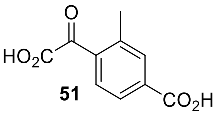 51	23% ± 6%	9% ± 3%	<1%	**>95%**	<5% (MES)<br><1% (HEPES)
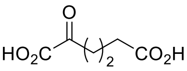 2OA (10)	**73% ± 16%**	14% ± 6%	<1%	**>95%**	<1% (MES)<br><1% (HEPES)
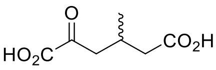 52	**48% ± 4%**	<5%	<1%	<1%	<1% (MES)<br><1% (HEPES)

a)2OG derivative	wt AspH	G434V AspH	R688Q AspH	R735Q AspH	R735W AspH
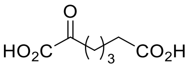 **2OP (11)**	22% ± 2%	<5%	<1%	**>95%**	<5% (MES)<br><1% (HEPES)
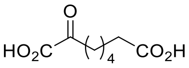 **2OS (12)**	<1%	<1%	<1%	**>95%**	8% ± 5% (MES)<br><1% (HEPES)

a) Chiral 2OG derivatives were prepared as racemic mixtures unless noted otherwise (10); b) mixture of racemic diastereomers, dr = 2.5:1; c) mixture of racemic diastereomers, dr = 2.5:1; d) mixture of racemic diastereomers, dr = 8:1; e) mixture of diastereomers, dr = 1:1; f) mixture of racemic diastereomers, dr = 5:1; g) mixture of diastereomers, dr = 8:1; h) (±)-(*2-exo,3-endo*)-diastereomer. Results are means of independent triplicates (n = 3; mean ± SD); 2OG derivatives showing ≳40% cosubstrate activity are in red.

Supporting Table S3. Effects of hydrophobic 2-oxoacids without a 2OG C5 carboxylate-equivalent group on catalysis by isolated recombinant human wt AspH and Traboulsi Syndrome-associated AspH variants (continues on the following page). SPE-MS AspH assays were performed as reported (2, 9), using: wt AspH$_{315-758}$ (0.1 μM) or G434V His$_6$-AspH$_{315-758}$ (0.1 μM), incubated with hFX-EGFD1$_{86-124}$-4S (1, 2) (4.0 μM), L-ascorbic acid (100 μM), $(NH_4)_2Fe(SO_4)_2{\cdot}6H_2O$ (20 μM), and a 2-oxoacid (500 μM) in buffer (25 mM HEPES, pH 7.5, 50 mM NaCl) for 15 min at 20 ºC; or: R688Q His$_6$-AspH$_{315-758}$ (0.2 μM), incubated with hFX-EGFD1$_{86-124}$-4S (4.0 μM), L-ascorbic acid (400 μM), $(NH_4)_2Fe(SO_4)_2{\cdot}6H_2O$ (100 μM), and a 2-oxoacid (50 μM) in buffer (25 mM MES, pH 6.0) for 30 min at 20 ºC; or: R735Q His$_6$-AspH$_{315-758}$ (0.2 μM) or R735W His$_6$-AspH$_{315-758}$ (0.2 μM), incubated with hFX-EGFD1$_{86-124}$-4S (4.0 μM), L-ascorbic acid (400 μM), $(NH_4)_2Fe(SO_4)_2{\cdot}6H_2O$ (100 μM), and a 2-oxoacid (2000 μM) in buffer (25 mM MES, pH 6.0) for 3 h at 20 ºC. Note that the activity of R735W AspH with 2-oxoacids was also tested in another buffer (25 mM HEPES, pH 7.5, 50 mM NaCl) under otherwise identical conditions, because R735W AspH did not show activity with 2OG in MES buffer.

Notably, the results reveal that the reactivity of 2-oxoacids with AspH variants can differ with the reaction conditions and the type of substitution. For example, the methionine transamination product **14** sustains catalysis by R735Q AspH, but not R735W AspH. By contrast, 2-oxoacid **19** sustains catalysis by R735W AspH more efficiently than by R735Q AspH, however, only in MES buffer but not in HEPES buffer.

[a]2-oxoacid	wt AspH	G434V AspH	R688Q AspH	R735Q AspH	R735W AspH
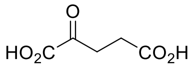 **2OG**	**>95%**	**>95%**	35% ± 3%	**62% ± 10%**	<1% (MES)<br><1% (HEPES)
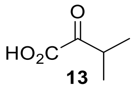 **13**	<1%	<1%	<1%	**>95%**	**>95%** (MES)<br>**94% ± 4%** (HEPES)
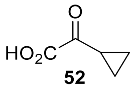 **52**	<1%	<1%	<1%	**54% ± 2%**	27% ± 5% (MES)<br>26% ± 3% (HEPES)
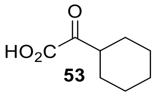 **53**	<1%	<1%	<1%	**>95%**	**>95%** (MES)<br>**>95%** (HEPES)
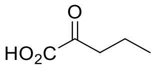 **54**	<1%	<1%	<1%	**>95%**	**>95%** (MES)<br>**>95%** (HEPES)
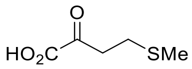 **14**	<1%	<1%	<1%	**86% ± 24%**	<5% (MES)<br><5% (HEPES)
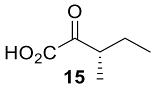 **15**	<1%	<1%	<1%	**>95%**	**>95%** (MES)<br>**95% ± 3%** (HEPES)
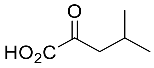 **16**	<1%	<1%	<1%	**>95%**	**>95%** (MES)<br>**63% ± 8%** (HEPES)

[a]2-oxoacid	wt AspH	G434V AspH	R688Q AspH	R735Q AspH	R735W AspH
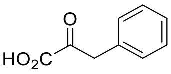 **17**	<1%	<1%	<1%	<1%	<1% (MES)<br><1% (HEPES)
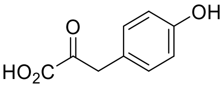 **55**	<1%	<1%	<1%	<1%	<1% (MES)<br><1% (HEPES)
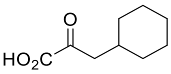 **18**	<1%	<1%	<1%	**>95%**	**64% ± 15%** (MES)<br>5% ± 3% (HEPES)
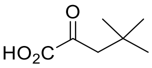 **19**	<1%	<1%	<1%	21% ± 2%	**40% ± 5%** (MES)<br>10% ± 5% (HEPES)
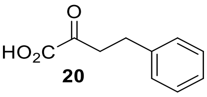 **20**	<1%	<1%	<1%	**>95%**	**>95%** (MES)<br>**52% ± 9%** (HEPES)

a) Results are means of independent triplicates (n = 3; mean ± SD); 2-oxoacids showing >40% cosubstrate activity are in red.

Supporting Table S4. Kinetic parameters of isolated recombinant human wt AspH and Traboulsi Syndrome-associated AspH variants for selected 2-oxoacids (continues on the following page). SPE-MS AspH assays were performed as reported (2, 9), using: wt AspH$_{315\text{-}758}$ (0.1 µM) or G434V His$_6$-AspH$_{315\text{-}758}$ (0.1 µM), hFX-EGFD1$_{86\text{-}124}$-4S (1, 2) (4.0 µM), L-ascorbic acid (100 µM), and Fe(II) $(NH_4)_2Fe(SO_4)_2 \cdot 6H_2O$ (20 µM) in buffer (25 mM HEPES, pH 7.5, 50 mM NaCl) at 20 ºC; or: R688Q His$_6$-AspH$_{315\text{-}758}$ (0.2 µM), hFX-EGFD1$_{86\text{-}124}$-4S (4.0 µM), L-ascorbic acid (400 µM), and $(NH_4)_2Fe(SO_4)_2 \cdot 6H_2O$ (100 µM) in buffer (25 mM MES, pH 6.0) at 20 ºC; or: R735Q His$_6$-AspH$_{315\text{-}758}$ (0.1 µM) or R735W His$_6$-AspH$_{315\text{-}758}$ (0.1 µM), hFX-EGFD1$_{86\text{-}124}$-4S (4.0 µM), L-ascorbic acid (400 µM), and $(NH_4)_2Fe(SO_4)_2 \cdot 6H_2O$ (200 µM) in buffer (25 mM MES, pH 6.0) at 20 ºC; experiments were performed using the 2-oxoacid concentrations given in Supporting Figures S15-S20.

[a]cosubstrate	AspH variant	k_{cat}^{app} [s^{-1}]	K_m^{app} [µM]	k_{cat}^{app}/K_m^{app} [mM^{-1}·s^{-1}]
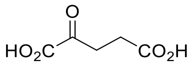 2OG	**wt AspH**	0.31 ± 0.03	1.1 ± 0.4	280 ± 110
	**G434V AspH**	0.69 ± 0.03	0.89 ± 0.13	770 ± 110
	**R688Q AspH**	0.015 ± 0.001	0.81 ± 0.15	18 ± 4
	**R735Q AspH**	0.01 ± 0.01	70 ± 20	0.09 ± 0.03
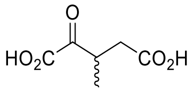 1	**wt AspH**	0.13 ± 0.01	1.6 ± 0.4	81 ± 17
	**G434V AspH**	0.20 ± 0.01	2.3 ± 0.3	82 ± 9
	**R688Q AspH**	0.03 ± 0.01	2.5 ± 0.4	11 ± 2
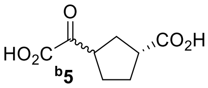 [b]5	**wt AspH**	0.07 ± 0.01	8.2 ± 0.6	8.9 ± 0.7
	**R735Q AspH**	0.01 ± 0.01	170 ± 35	0.05 ± 0.02
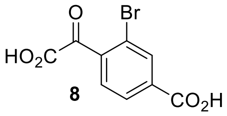 8	**wt AspH**	0.09 ± 0.01	120 ± 17	0.73 ± 0.11
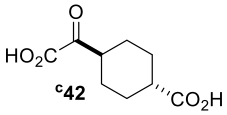 [c]42	**R735Q AspH**	0.02 ± 0.01	1100 ± 150	0.02 ± 0.01
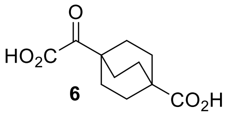 6	**R735Q AspH**	0.04 ± 0.01	3000 ± 600	0.01 ± 0.01
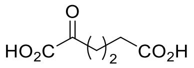 2OA (10)	**wt AspH**	0.11 ± 0.01	4.7 ± 0.7	24 ± 4
	**R735Q AspH**	0.03 ± 0.01	85 ± 23	0.34 ± 0.10
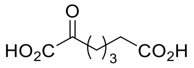 2OP (11)	**R735Q AspH**	0.38 ± 0.03	550 ± 97	0.68 ± 0.13
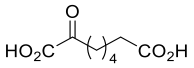 2OS (12)	**R735Q AspH**	0.72 ± 0.07	640 ± 160	1.1 ± 0.3
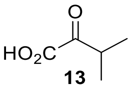 13	**R735Q AspH**	0.21 ± 0.02	300 ± 79	0.70 ± 0.20
	**R735W AspH**	0.11 ± 0.01	240 ± 42	0.46 ± 0.09

[a]cosubstrate	AspH variant	k_{cat}^{app} [s^{-1}]	K_m^{app} [µM]	k_{cat}^{app}/K_m^{app} [$mM^{-1}\cdot s^{-1}$]
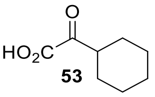 **53**	**R735Q AspH**	0.09 ± 0.05	700 ± 470	0.13 ± 0.11
	**R735W AspH**	0.09 ± 0.01	150 ± 27	0.60 ± 0.11
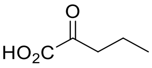 **54**	**R735Q AspH**	0.07 ± 0.01	76 ± 8	0.93 ± 0.11
	**R735W AspH**	0.04 ± 0.01	82 ± 9	0.44 ± 0.05
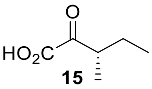 **15**	**R735Q AspH**	0.16 ± 0.04	370 ± 110	0.44 ± 0.16
	**R735W AspH**	0.10 ± 0.01	250 ± 37	0.42 ± 0.07
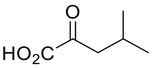 **16**	**R735Q AspH**	0.05 ± 0.02	150 ± 71	0.35 ± 0.19
	**R735W AspH**	0.08 ± 0.03	240 ± 96	0.36 ± 0.17
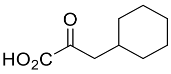 **18**	**R735Q AspH**	0.03 ± 0.01	270 ± 150	0.09 ± 0.06
	**R735W AspH**	0.02 ± 0.01	210 ± 46	0.10 ± 0.03
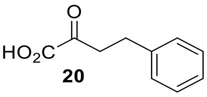 **20**	**R735Q AspH**	0.07 ± 0.01	87 ± 15	0.78 ± 0.14
	**R735W AspH**	0.05 ± 0.01	390 ± 120	0.12 ± 0.04

a) SPE-MS analyses and Michaelis Menten curves used to determine steady state kinetic parameters are shown in Supporting Figures S15-S20; b) mixture of diastereomers, dr = 1:1; c) mixture of racemic diastereomers, dr = 5:1. Results are means of independent triplicates (n = 3; mean ± SD).

Supporting Table S5. Data collection and refinement statistics for the R688Q AspH:Mn:(3*R*)-3-methyl-2OG (1):hFX-EGFD1$_{86-124}$-4S and R735Q AspH:Mn:2-oxosuberate (2OS, 12):hFX-EGFD1$_{86-124}$-4S complexes.

**Datasets**	**R688Q AspH:Mn:(3*R*)-3-methyl-2OG (1):hFX-EGFD1$_{86-124}$-4S** (PDB ID: 8RE8)	**R735Q AspH:Mn:2-oxosuberate (2OS,12):hFX-EGFD1$_{86-124}$-4S** (PDB ID: 8RE5)
**Data Collection (T in K)**	MX (100)	MX (100)
Beamline (Wavelength, Å)	DLS I03 (0.9763)	DLS I03 (0.9763)
Detector	EIGER2 XE 16M	EIGER2 XE 16M
Data Processing	Xia2, DIALS	Xia2, DIALS
Space group	$P\,2_1\,2_1\,2_1$	$P\,2_1\,2_1\,2_1$
Cell dimensions:		
a,b,c (Å)	50.6, 86.0, 124.0	50.4, 86.3, 123.8
α, β, γ (°)	90, 90, 90	90, 90, 90
No. of molecules/ASU	1	1
No. reflections	47230 (2317)*	60246 (2967)*
Resolution (Å)	46.85-1.85 (1.88-1.85)*	61.9-1.70 (1.73-1.70)*
R_{merge}	0.130 (1.954)*	0.073 (1.139)*
I/σI	13.4 (1.2)*	17.70 (1.70)*
CC-half	0.998 (0.652)*	0.999 (0.713)*
Completeness (%)	100 (99.4)*	100 (99.9)*
Multiplicity	13.30 (13.00)*	13.30 (10.00)*
Wilson B value (Å^2)	27.84	25.21
**Refinement**	PHENIX	PHENIX
R_{work}/R_{free+}	0.1772/0.2082	0.1756/0.2072
No. atoms	3941	4064
- Enzyme	3584	3586
- ligand	41	28
- Water	316	450
Average B-factors	39.39	29.77
- Enzyme	38.82	26.08
- ligand	45.83	34.97
- Water	45.04	39.05
R.m.s deviations		
- Bond lengths (Å)	0.004	0.015
- Bond angles (°)	0.61	1.32

*Highest resolution shell in parentheses. DLS: Diamond Light Source; ASU: asymmetric unit.

3. Supporting references

1. Pfeffer, I., Brewitz, L., Krojer, T., Jensen, S. A., Kochan, G. T., Kershaw, N. J., *et al.* (2019) Aspartate/asparagine-β-hydroxylase crystal structures reveal an unexpected epidermal growth factor-like domain substrate disulfide pattern. *Nat. Commun.* **10**, 4910
2. Brewitz, L., Tumber, A., and Schofield, C. J. (2020) Kinetic parameters of human aspartate/asparagine-β-hydroxylase suggest that it has a possible function in oxygen sensing. *J. Biol. Chem.* **295**, 7826-7838
3. Brewitz, L., Onisko, B. C., and Schofield, C. J. (2022) Combined proteomic and biochemical analyses redefine the consensus sequence requirement for epidermal growth factor-like domain hydroxylation. *J. Biol. Chem.* **298**, 102129
4. Fernlund, P., and Stenflo, J. (1983) β-Hydroxyaspartic acid in vitamin K-dependent proteins. *J. Biol. Chem.* **258**, 12509-12512
5. McMullen, B. A., Fujikawa, K., Kisiel, W., Sasagawa, T., Howald, W. N., Kwa, E. Y., *et al.* (1983) Complete amino acid sequence of the light chain of human blood coagulation factor X: evidence for identification of residue 63 as β-hydroxyaspartic acid. *Biochemistry* **22**, 2875-2884
6. Geoghegan, K. F., Dixon, H. B. F., Rosner, P. J., Hoth, L. R., Lanzetti, A. J., Borzilleri, K. A., *et al.* (1999) Spontaneous α-N-6-Phosphogluconoylation of a “His Tag” in Escherichia coli: The Cause of Extra Mass of 258 or 178 Da in Fusion Proteins. *Anal. Chem.* **267**, 169-184
7. Yan, Z., Caldwell, G. W., and McDonell, P. A. (1999) Identification of a Gluconic Acid Derivative Attached to the N-Terminus of Histidine-Tagged Proteins Expressed in Bacteria. *Biochem. Biophys. Res. Commun.* **262**, 793-800
8. Brewitz, L., Brasnett, A., Schnaubelt, L. I., Rabe, P., Tumber, A., and Schofield, C. J. (2024) Methods for production and assaying catalysis of isolated recombinant human aspartate/asparagine-β-hydroxylase In: Methods Enzymol., Academic Press, **704**, 313-344
9. Brasnett, A., Pfeffer, I., Brewitz, L., Chowdhury, R., Nakashima, Y., Tumber, A., *et al.* (2021) Human oxygenase variants employing a single protein Fe^{II} ligand are catalytically active. *Angew. Chem. Int. Ed.* **60**, 14657-14663
10. Brewitz, L., Nakashima, Y., and Schofield, C. J. (2021) Synthesis of 2-oxoglutarate derivatives and their evaluation as cosubstrates and inhibitors of human aspartate/asparagine-β-hydroxylase. *Chem. Sci.* **12**, 1327-1342
